# Plastome variations reveal the distinct evolutionary scenarios of plastomes in the subfamily Cereoideae (Cactaceae)

**DOI:** 10.1186/s12870-023-04148-4

**Published:** 2023-03-08

**Authors:** Jie Yu, Jingling Li, Youwei Zuo, Qiulin Qin, Siyuan Zeng, Heinz Rennenberg, Hongping Deng

**Affiliations:** 1 Key Laboratory of Horticulture Science for Southern Mountainous Regions, Ministry of Education, College of Horticulture and Landscape Architecture, Southwest University, Chongqing, 400716 China; 2 Center for Biodiversity Conservation and Utilization, School of Life Sciences, Southwest University, Chongqing, 400715 China; 3 Center of Molecular Ecophysiology, College of Resources and Environment, Southwest University, Chongqing, 400715 China; 4 No. 2 Tiansheng Road, Beibei District, Chongqing, 400716 China

**Keywords:** Plastome, Inversions, Rearrangements, Cereoideae, Phylogenomics

## Abstract

**Background:**

The cactus family (Cactaceae) has been reported to have evolved a minimal photosynthetic plastome size, with the loss of inverted-repeat (IR) regions and NDH gene suites. However, there are very limited genomic data on the family, especially Cereoideae, the largest subfamily of cacti.

**Results:**

In the present study, we assembled and annotated 35 plastomes, 33 of which were representatives of Cereoideae, alongside 2 previously published plastomes. We analyzed the organelle genomes of 35 genera in the subfamily. These plastomes have variations rarely observed in those of other angiosperms, including size differences (with ~ 30 kb between the shortest and longest), dramatic dynamic changes in IR boundaries, frequent plastome inversions, and rearrangements. These results suggested that cacti have the most complex plastome evolution among angiosperms.

**Conclusion:**

These results provide unique insight into the dynamic evolutionary history of Cereoideae plastomes and refine current knowledge of the relationships within the subfamily.

**Supplementary Information:**

The online version contains supplementary material available at 10.1186/s12870-023-04148-4.

## Background

The cactus family (Cactaceae) contains charismatic ornamental horticultural plants, which belong to the order Caryophyllales, with approximately 174 genera and nearly 2,000 species [1, 2]. Cactaceae are mostly native to tropical or subtropical deserts or arid areas of America except for epiphytic species, such as *Rhipsalis baccifera* (J.S.Muell.) Stearn, which is also found naturally in East Africa, Madagascar, and Sri Lanka [3, 4]. They exhibit a range of life forms from geophytes and cushion plants to dwarf shrubs, shrubs, or small trees [5]. To adapt to the unique climatic environment, cacti have evolved morphological and anatomical characteristics that are different from those of most angiosperms, such as highly specialized leaves, succulent tissues, and even aerial roots [6–8], which are of general interest to plant biologists. Physiologically, crassulacean acid metabolism (CAM) in cacti is a feature uncommon to most angiosperms and considered an adaptation to allow survival in arid and water-deficient environments [9, 10]. In recent studies, significant expansion of stress adaptation-related genes and more restrictive gene duplication events have been reported in harsh environment-adapted lineages of Caryophyllales, including cacti [11]. The chromosome-level genomes of *Selenicereus undatus* (Haw.) D. R. Hunt and *Carnegiea gigantea* (Engelm.) Britton & Rose confirmed the presence of whole-genome duplication (WGD) events in cacti [12, 13], which are typically considered to be associated with shifts in climatic niches.

As the site of photosynthesis, the chloroplast is an essential organelle of all autotrophic plants. It contains a semiautonomous genetic system, which is called the chloroplast genome (cpDNA) or plastid genome/plastome. In cacti, as a group of plants with the CAM pathway, plastome-level changes can be expected. Although the plastomes of most terrestrial angiosperms are thought to be extremely conserved, several exceptions have been reported in some clades. In addition to large plastome losses in some nonphotosynthetic plants, such as *Cuscuta* [14] and *Gastrodia* [15], independent losses of the inverted repeat region have been identified across disparate clades, such as Fabaceae [16], Geraniaceae [17], Orobanchaceae [18], and Cactaceae [19]. Most plastid genomes of Caryophyllales range in size from 151 kb to 155 kb [20]. Structurally, two single-copy regions (SC) are separated by a pair of inverted repeats (IRb and IRa, ~ 25 kb), forming a typical tetrad structure. However, as a special case of Caryophyllales, large losses of the IR regions have been observed in all reported cactus plastomes to date [4, 19]. In addition, a conserved inversion of ~ 6 kb in the large single-copy unit comprising the *trn*M-*rbc*L genes has been reported in some Caryophyllales plants [21]. In addition, the loss of *ndh* genes in Cactaceae was described for the first time in *Carnegiea gigantea*[19]. Thus, the plastomes of the cactus family seem to be different from those of other Caryophyllales. A recent report on the plastomes of Opuntioideae addresses the phylogenetic relationships of all genera in this subfamily [22]. However, the plastome assembly contained many degenerate bases. Therefore, a broad sampling of data is needed, especially for Cereoideae lineages, to provide new insights into plastome evolution across the (sub)family.

Taxonomic studies of Cactaceae have identified five major lineages based on morphological characteristics combined with molecular phylogenies, including two widespread and species-rich subfamilies: Cactoideae and Opuntioideae [23]; the monogeneric subfamily Maihuenioideae [24]; and the traditional “Pereskioideae”, which has been divided into two leafy lineages, Leuenbergerioideae [25] and Pereskioideae. The two leafy lineages have been recognized as subsequent sisters to all the other lineages of Cactaceae. Recently, the relationships of all genera in Opuntioideae have been determined [22]. Cactoideae is the largest subfamily of Cactaceae, with more than 100 generally accepted genera [26]. The monospecific genus *Blossfeldia* (*Blossfeldia liliputana* Werderm. ) was considered the basal clade of Cactoideae and later recognized as a separate tribe (Blossfeldieae) [27]. Tribe Cacteae is the earliest-diverging clade in Cactoideae except for Blossfeldieae and has traditionally been recognized as a monophyletic group [28]. Furthermore, multiple tribes previously reported, i.e., Pachycereeae, Hylocereeae, Browningieae, Trichocereeae, Cereeae, Rhipsalideae, and Notocacteae, were found to be para- or polyphyletic [29, 30]. With the aid of molecular phylogenies, several clades were successively identified as monophyletic groups and recognized as monophyletic tribes [26, 31]. The positions of several genera, such as *Calymmanthium* and *Frailea*, which were weakly supported and unresolved within Cactoideae, are uncertain [26, 28]. Although recent studies have greatly improved knowledge of the phylogenetic relationships of Cactoideae, information on weakly supported clades still needs to be strengthened. Plastid genomes, with their conserved structure and abundant phylogenetic information sites, have been proven to be efficient in the study of phylogenomics [20] and have previously played an important role in solving the phylogenetic relationships of the subfamily Opuntioideae [22]. However, they have not been widely used in Cactoideae, and only a few Cactoideae plastomes are available in the GenBank database. This lack of genomic data limits our understanding of biodiversity in the largest subfamily of Cactaceae.

We previously assembled the complete plastomes of four *Selenicereus* species [32] and found the unusual boundary of inverted repeats. We hypothesized that the plastomes of Cactoideae species display unusual variations. To further study this less-reported group of plants, we newly assembled 35 cactus plastomes, including those of 33 species from the subfamily Cactoideae that we are interested in. Our collections covered almost all tribes and disputed clades, representing the most extensive sampling to date. Two additional species were used as outgroups: *Pereskia aculeata* Mill. from Pereskioideae and *Opuntia microdasys* (Lehm.) Pfeiff. from Opuntioideae. This enables us to fully analyze the evolution of Cereoideae plastomes and to understand the phylogenetic relationships of the main tribe.

## Results

### Structural characteristics of the cactus plastomes

The deciphered Cactaceae plastome sizes ranged from 110,388 bp to 143,783 bp (Table 1), and the GC content ranged from 35.80 to 37.53%. We found that not all these plastomes have a typical tetrad structure. Some of them contained a pair of typical inverted repeat (IRs) regions that mediate two single-copy (SC) regions, while others exhibited significant losses of IRs. The IR regions were found to have a wide range of lengths, with the shortest at 358 bp (*Thelocactus setispinus* (Engelm.) E.F. Anderson) and the longest at 37,186 bp (*Acanthocereus Tetragonus* (L.) Hummelinck), indicating that the IR regions had undergone large-scale expansion/contraction. It is important to note that for the two previously reported species (*Ca. gigantea* and *Lo. schottii*), no IR region was found, and we could not determine whether there was a problem with the previous assembly, as it might have been short enough to be overlooked. Furthermore, the large single-copy (LSC) region and the small single-copy (SSC) region differed greatly in length, ranging from 48,479 bp ~ 88,758 bp and 10,467 bp ~ 34,726 bp, respectively. The length variation of the single-copy region was closely related to the dynamic changes in IR regions.

**Table 1:** Characteristics of the 37 Cactaceae plastomes

Species	NCBI Accession No.	Genome Size (bp)	LSC (bp)	SSC (bp)	IR (bp)	Total GC (%)	No. total Genes	No. PCGs	No.	No.
									tRNAs	rRNAs
*Cereus jamacaru*	/	143,493	53,377	23,778	33,169	36.64	95 (25)	64 (12)	27 (13)	4
*Acanthocereus tetragonus*	MW553073	142,663	53,494	23,777	32,696	36.64	95 (25)	64 (12)	27 (13)	4
*Praecereus euchlorus*	/	135,580	51,279	10,467	36,917	36.67	95 (28)	64 (14)	27 (14)	4
*Pilosocereus pachycladus*	MW553065	133,372	52,759	19,329	30,642	36.56	94 (25)	63 (12)	27 (13)	4
*Coleocephalocereus fluminensis*	/	128,523	52,445	21,314	27,382	36.31	95 (25)	64 (12)	27 (13)	4
*Gymnocalycium saglionis*	MW553054	124,390	50,666	16,326	28,699	37.09	92 (25)	61 (12)	27 (13)	4
*Espostoa lanata*	MW553052	127,175	50,979	19,440	28,378	37.01	96 (25)	64 (12)	28 (13)	4
*Matucana haynei*	MW553051	126,870	51,077	19,219	28,287	37.08	96 (25)	64 (12)	28 (13)	4
*Cleistocactus winteri*	MW553046	128,809	50,944	18,855	29,505	37.02	96 (25)	63 (12)	29 (13)	4
*Echinopsis mirabilis*	/	126,622	52,505	18,915	27,601	37.18	92 (25)	61 (12)	27 (13)	4
*Rebutia pygmaea*	/	124,473	53,272	18,843	26,179	37.53	94 (21)	62 (11)	28 (10)	4
*Parodia scopa*	MW553045	126,253	48,479	18,092	29,841	36.84	96 (26)	64 (13)	28 (13)	4
*Yavia cryptocarpa*	/	143,783	53,677	15,734	37,186	36.35	96 (26)	64 (15)	28 (11)	4
*Rhipsalis cereuscula*	MW553066	123,009	79,737	22,890	10,191	36.59	99 (4)	66 (2)	29 (2)	4
*Schlumbergera truncata*	MW553067	121,718	79,780	21,978	9,980	36.33	99 (4)	67 (2)	28 (2)	4
*Frailea castanea var. nitens*	MW553053	110,388	81,002	28,512	437	36.31	94 (2)	62 (0)	28 (2)	4
*Cephalocereus senilis*	/	120,812	81,561	21,267	8,992	36.59	95 (4)	63 (2)	28 (2)	4
*Neobuxbaumia polylopha*	MW553061	128,889	80,969	13,942	16,989	36.39	94 (7)	62 (3)	28 (4)	4
*Carnegiea gigantea*	NC_027618.1	113,064	/	/	No IR	36.68	96 (0)	64 (0)	28 (0)	4
*Lophocereus schottii*	NC_041727.1	113,204	/	/	No IR	36.5	96 (0)	64 (0)	28 (0)	4
*Deamia testudo*	/	121,008	81,371	21,909	8,864	36.59	95 (2)	63 (0)	28 (2)	4
*Myrtillocactus geometrizans*	MW553060	133,639	81,748	15,835	18,028	35.97	97 (8)	65 (5)	28 (3)	4
*Echinocereus pentalophus*	MW553049	128,011	81,452	18,873	13,843	36.28	97 (6)	65 (2)	28 (4)	4
*Selenicereus undatus*	MW553056	133,407	68,516	21,773	21,559	36.38	98 (16)	65 (8)	29 (8)	4
*Epiphyllum oxypetalum*	MW553050	120,360	81,611	21,043	8,853	36.56	96 (3)	64 (1)	28 (2)	4
*Copiapoa hypogaea*	MW553047	132,285	82,845	20,600	14,420	35.8	100 (6)	67 (4)	29 (2)	4
*Calymmanthium substerile*	/	120,777	81,982	24,061	7,367	36.59	100 (3)	67 (1)	29 (2)	4
*Leuchtenbergia principis*	MW553057	116,863	81,621	32,158	1,542	36.18	96 (2)	63 (1)	29 (1)	4
*Thelocactus setispinus*	MW553071	110,524	78,924	30,884	358	36.17	94 (0)	61 (0)	29 (0)	4
*Ferocactus latispinus*	MW553072	116,692	82,072	31,682	1,469	36.25	96 (2)	63 (1)	29 (1)	4
*Echinocactus grusonii*	MW553048	119,201	82,732	32,771	1,849	36.13	100 (2)	67 (1)	29 (1)	4
*Mammillaria gracilis*	MW553059	110,855	77,223	30,246	1,693	36.46	95 (2)	63 (1)	28 (1)	4
*Obregonia denegrii*	MW553062	118,438	74,449	31,769	6,110	36.67	94 (6)	62 (3)	28 (3)	4
*Ariocarpus retusus*	MW553043	117,264	81,642	32,170	1,726	36.12	98 (2)	66 (1)	28 (1)	4
*Astrophytum myriostigma*	MW553044	119,769	83,710	32,131	1,964	35.92	102 (4)	69 (3)	29 (1)	4
*Opuntia microdasys*	MW553063	123,169	88,758	31,493	1,459	36.09	111 (3)	77 (0)	30 (3)	4
*Pereskia aculeata*	MW553064	141,752	88,214	34,726	9,406	35.8	113 (5)	79 (3)	30 (2)	4

Note. The number of genes with two copies was given in parentheses

### Gene annotation

**Fig. 1:**
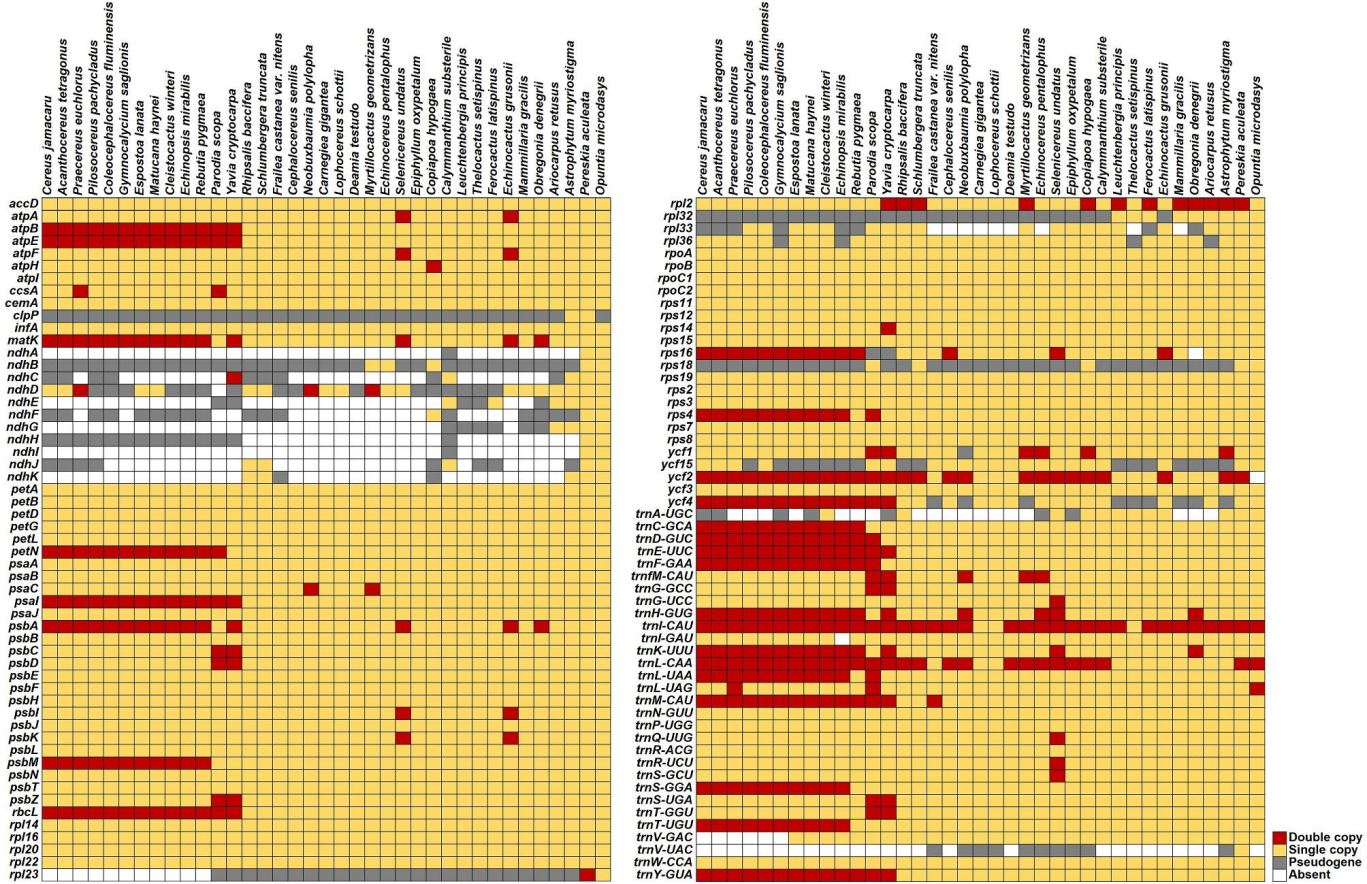
Gene comparisons among the 37 Cactaceae plastomes. Each red square indicates two copies of the gene, the yellow square indicates that the gene has one copy, the gray square indicates a gene fragment or pseudogene, and the white indicates that the gene was absent

The results of gene annotation showed that the total number of unique protein-coding genes (PCGs) in our assembled plastomes differed greatly. To analyze differences in the number of PCGs, the plastome of a closely related species, *Portulaca oleracea* L., was compared with the 37 plastomes of the present study. In purslane (*Po. oleracea*), the number of unique PCGs was 80, closest to that in *Pe. aculeata* (79), which is a kind of cactus with leaves from the subfamily Pereskioideae, followed by *Op*. *microdasys* from the subfamily Opuntioideae with 77 annotations (Fig. 1; Table 1). For the other 35 Cereoideae plastomes, the number of unique PCGs ranged from 61 to 69, indicating that gene gain/loss events occurred in the subfamily Cereoideae. Figure S1 shows the circular genome map of these 35 plastomes.

We further used the Basic Local Alignment Search Tool (BLAST) to identify the homologous sequences of PCGs, and several gene gains/losses and pseudogenization events were identified. For the PCGs, first, the widespread loss/pseudogenization of the *ndh* gene suite in subfamily Cereoideae, as described earlier in *Ca. gigantea* [19], was most striking. Only a few *ndh* genes were retained in a few plastomes (Fig. 1). Furthermore, several genes (*rpl23*, *rpl33*, *rps16*, and *ycf2*) were also observed to be lost in some plastomes. For example, the *ycf2* gene, as a large open reading frame of the plastome, was absent in *Op*. *microdasys*. Moreover, some genes were detected in plastomes as only a remnant fragment, including *accD*, *clpP*, *rpl23*, *rpl32*, *rpl33*, *rpl36*, *rps18*, and *ycf15* (Fig. 1). Another type of gene pseudogenization showed premature termination codons, such as *ycf1* and *ycf4*. The *clpP* genes exist as pseudogenes in most species, mainly due to the loss of the first exon.

All species had the same four unique ribosome RNA (rRNA) genes, namely, 4.5 S rRNA, 5 S rRNA, 16 S rRNA, and 23 S rRNA, which were present as single copies in all 37 plastomes. However, the number of unique tRNA genes was different and ranged from 27 to 30 (Table 1). The *trnA-UGC* and *trnV-GAC* genes were completely or partially lost in some plastomes. Furthermore, the *trnV-UAC* gene was completely or partially lost in all 35 Cereoideae plastomes (Fig. 1). The *trnI-GAU* gene was lost only in *Echinopsis mirabilis* Speg.

As reported by Yao [20], intron loss events were unique to some lineages of Caryophyllales. For example, *rpl2* intron losses were a common feature of the order Caryophyllales, and *rpoC1* intron losses were unique to the lineage of Cereoideae. These findings were further confirmed in the present study.

### Repeat elements

We analyzed the repeat elements of the 35 newly assembled plastomes. ROUSFinder was used to identify the nonredundant repeat units. The results showed that the number of these repeating elements varied greatly in different plastomes. The plastome with the largest number was that of *Myrtillocactus geometrizans* (Mart. ex Pfeiff.) Console, with 73 different repeat units, and the total number of repeat units was more than 400, while the plastome with the fewest repeat elements was that of *Matucana haynei* (Otto ex Salm-Dyck) Britton & Rose, with only 7 different repeat units, and the total number of repeat units was only 19 (Table S1). Details of each identified repeat element are shown in Table S2. We also analyzed the location of these repeats in the plastomes. Most of the repeats were located near the *accD* gene of the plastome. Among 4,931 repeat units in 35 plastomes, 1,013 appeared in the internal or upstream regions of *accD*, accounting for 20.54% of the total number of repeat units (Tables S2-S3. Next were the *ycf1*, *ycf3*, *rps19*, *rps18*, *rps15*-*psaC* and *trnT-CGU* genes. The repeat units in these regions accounted for nearly half of the total number of repeats, which were the hotspot regions of repeat elements in the cactus plastome.

**Fig. 2:**
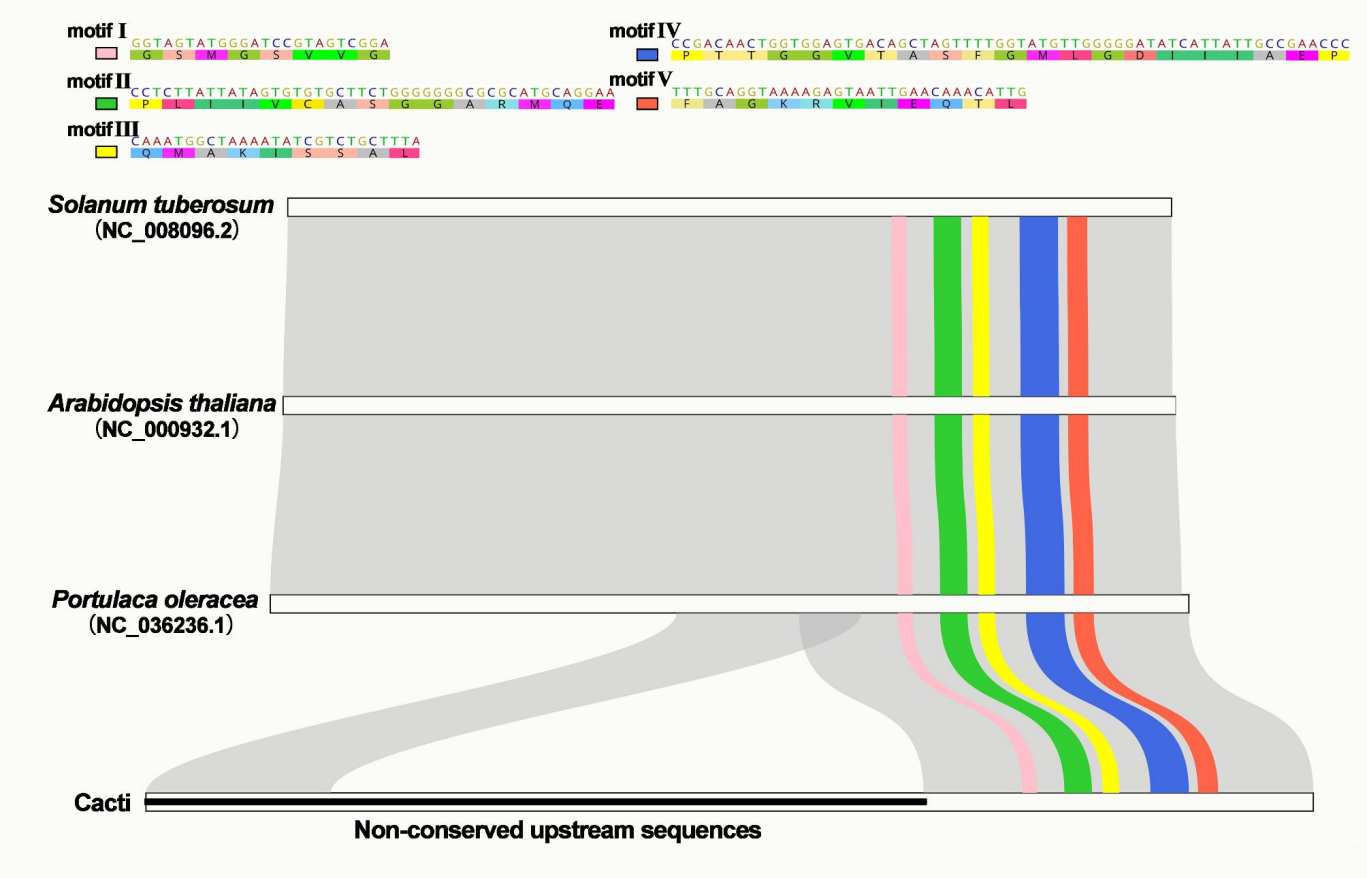
A potential new pattern of *accD* genes in cactus plastomes. We performed multiple alignments of amino acid sequences of *accD* genes from *Solanum tuberosum* (NC_008096.2), *Arabidopsis thaliana* (NC_000932.1), *Portulaca oleracea* (NC_036236.1) and cactus plastomes. The downstream region of the *accD* gene is highly conserved in these species and contains five conserved motifs, of which motifI contains the carboxyl-biotin binding sites and motifII contains the potential catalytic site. In the figure, 5 conserved motifs are shown at the top, while the multisequence alignment diagram is shown at the bottom. The gray ribbon represents homologous sequences, among which 5 conserved motifs are highlighted with different colors. The downstream domain of the *accD* gene is conserved, but in cactus species, the upstream region has more repeats and is extremely nonconserved between species

It is noteworthy that these repeat elements are closely related to the generation of a new pattern of the *accD* gene. By analyzing the sequence similarity between the *accD* genes of cactus species and those *Arabidopsis thaliana* (L.) Heynh., *Solanum tuberosum* L. and *Po. oleracea*, we found that the internal/upstream *accD* gene of the cactus family was full of short repeat sequences, and these repeats near *accD* of different cacti lacked homology with each other (data not shown). Although cactus species have lost many sequences of *accD* genes, the functional domains of the *accD* gene, including the conserved motifs, remained complete (Fig. 2). These upstream repeats of *accD* genes lacked homology in different cactus species, forming extremely nonconserved open regions.

### Phylogenomic studies

**Fig. 3:**
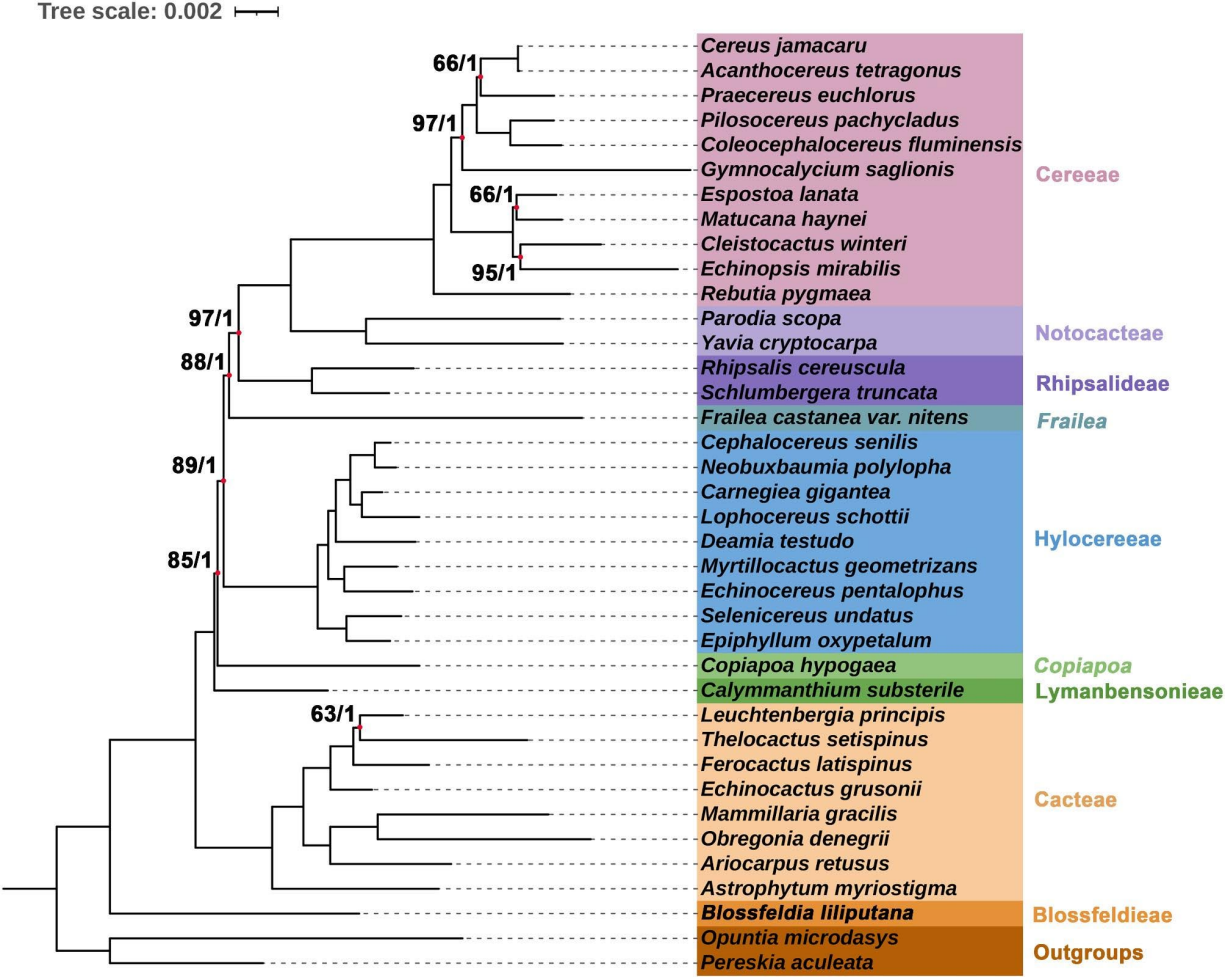
Phylogenetic tree reconstructed by maximum likelihood (ML) and Bayesian inference (BI) analysis based on the 57 shared plastid protein-coding genes of 38 cactus species. The 57 shared plastid protein-coding genes included *atpA*, *atpB*, *atpE*, *atpF*, *atpH*, *atpI*, *ccsA*, *cemA*, *infA*, *petA*, *matK*, *petB*, *petD*, *petG*, *petL*, *petN*, *psaA*, *psaB*, *psaC*, *psaI*, *psaJ*, *psbA*, *psbB*, *psbC*, *psbD*, *psbE*, *psbF*, *psbH*, *psbI*, *psbJ*, *psbK*, *psb*L, *psbM*, *psbN*, *psbT*, *psbZ*, *rbcL*, *rpl14*, *rpl16*, *rpl20*, *rpl22*, *rpl2*, *rpoA*, *rpoB*, *rpoC1*, *rpoC2*, *rps11*, *rps12*, *rps14*, *rps15*, *rps19*, *rps2*, *rps3*, *rps4*, *rps7*, *rps8* and *ycf3*. All nodes were fully resolved based on BI methods, and we highlighted the nine nodes that have not been fully resolved based on ML methods. The number at the bottom of the scale, 0.01, means that the length of the branch represents the replacement frequency of bases at each site of the genome at 0.01. We identified seven clades in the subfamily Cereoideae, and two genera (*Frailea* and *Copiapoa*) were separate taxa that do not belong to any of the above clades

Phylogenomic analyses were performed based on 57 conserved PCGs of 37 Cactaceae species. In this study, two species (*Pe. aculeata* and *Op. microdasys*) were used as outgroups to obtain a rooted phylogenetic tree. The two methods of maximum likelihood (ML) and Bayesian inference (BI) yielded trees with the same topology (Fig. 3). The accuracy of the inferred species phylogeny was strongly supported by the stability of the main clades generated and high bootstrap values.

BI and ML analyses revealed nine highly supported clades in the subfamily Cactoideae, which were also sister groups. The first clade closest to the root of the tree is the tribe Blossfeldieae, which contains only one species, namely, *B*. *liliputana*. The second clade is the tribe Cacteae, which is an early-diverging clade in Cactoideae, with high support values in the plastid gene phylogenetic tree. The third clade is the tribe Lymanbensonieae, and only one species in our sample (*Calymmanthium substerile* F. Ritter) belongs to this tribe. The fourth clade consists of one species (*Copiapoa hypogaea* F. Ritter), which is not grouped into a clade with all the other tribes. The fifth clade is the tribe Hylocereeae, containing 9 species here. The sixth clade also has only one species (*Frailea castanea var. nitens*), which does not belong to other tribes. The last three clades are tribes Rhipsalideae, Notocacteae, and Cereeae, respectively.

### Plastome inversions and rearrangements

**Fig. 4:**
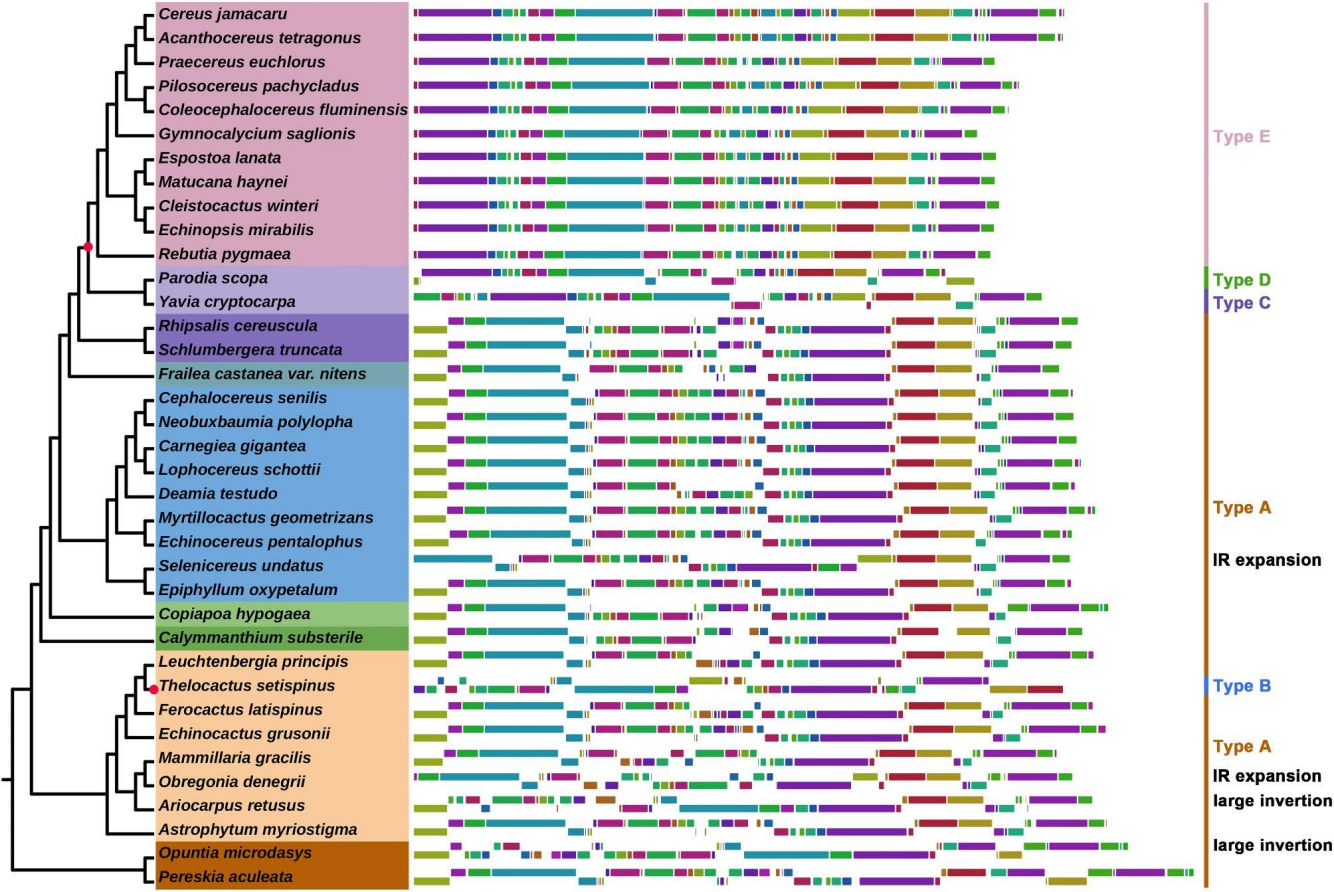
Plastomic locally collinear blocks (LCBs). We identified the LCBs of the plastid genomes based on the progressiveMauve module of Mauve software (v2.3.1) with default parameters. The last IR region was removed before calculation. Each LCB was drawn on the right side of the phylogenetic tree with different colors. According to the arrangement order of LCBs, these plastid genomes are divided into five different types (type A - type E). For the expansion of the IR region and the 60 kb inversion observed in *Opuntia microdasys* and *Ariocarpus retusus*, we mark ‘IR expansion’ and ‘large inversion’ on the far right, respectively. These results show that the cactus plastomes have undergone extensive rearrangement

**Fig. 5:**
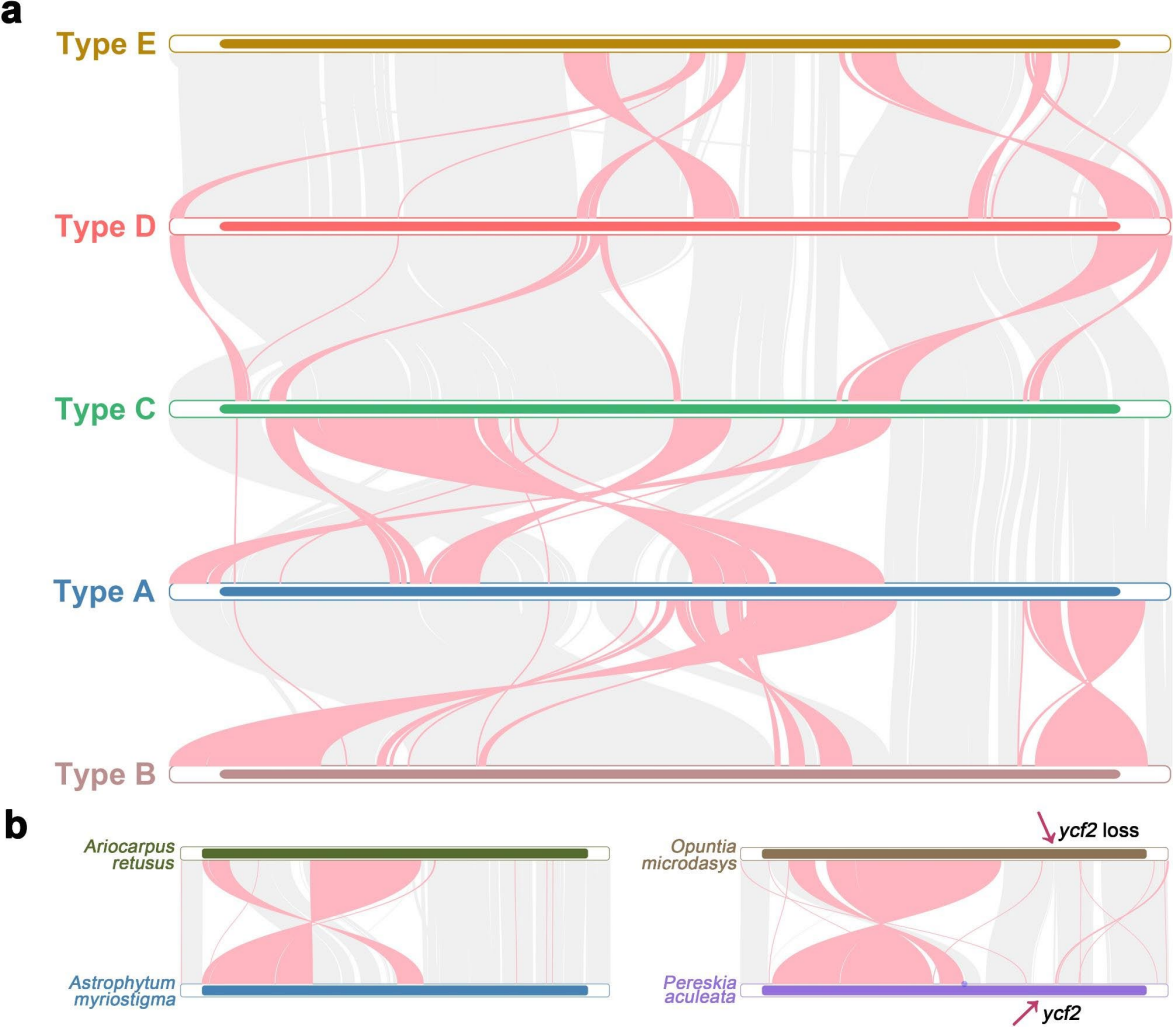
Pairwise comparisons of the plastid genomes. We use gray and red ribbons to represent highly homologous regions, where the gray ribbon represents the same direction and the red ribbon represents the opposite direction, that is, the inverted region of the genome. **(a)** Pairwise comparison of the five different plastid genome types. **(b)** A 60 kb inversion was observed in *Opuntia microdasys* and *Ariocarpus retusus* by comparison with related species. We marked the absence of *ycf2* in *Opuntia microdasys*

The Cereoideae plastomes experienced multiple genome recombination events during the evolutionary process, especially in several clades of Cereoideae (Fig. 4). Most strikingly, among the most recently evolved clades, tribe Notocacteae and Cereeae have a widely varied order of locally collinear blocks (LCBs), showing extensive plastome rearrangement. Furthermore, all 11 species in tribe Cereeae shared the same plastome recombination event, whereas the two species in tribe Notocacteae differed from each other and tribe Cereeae. Notably, one of the plastomes from tribe Cacteae, i.e., *Thelocactus setispinus* (Engelm.) E.F. Anderson, was also found to be different from that of all other plastomes in configuration. We reassembled and checked the short reads multiple times, all of which confirmed the abnormal plastome configuration. We described these differences in five different types (i.e., types A-E). Collinearity analysis showed that this recombination involved multiple independent rearrangements combined with inversions of LCBs (Fig. 5a). In addition, in some plastomes, we also found a difference in the order of LCBs due to IR expansion and a series of small inversions. They were generally local and regular, and we think of them as occurring under a basic framework configuration. Attention should be given to two relatively large 60 kb inversions (Fig. 5b). A self-dot plot for the assembled plastomes showed that this inversion was probably associated with a pair of inverted repeats. We did not find this inversion in the 14 plastomes that underwent significant recombination (i.e., those of *Thelocactus Setispinus*, tribe Cereeae, and Notocacteae). However, this type of inverted repeat was detected in 20 of the remaining 23 plastomes, showing its common presence and implying the possibility of isomeric plastomes (Table S4 and Fig. S2).

The other small rearrangements are commonly but irregularly found in cactus plants (Figs. S3-S5). The only certainty was that fragment losses in the SSC region were widespread. Using *Po. oleracea* as a reference, the SSC region of *Pe. aculeata* had a rearrangement event, and this event remained in *Op. microdasys* (Fig. S3). Subsequently, another rearrangement occurred in the subfamily Cereoideae. This rearrangement was accompanied by the deletion of multiple fragments in the SSC region, resulting in the loss of the *ndh* gene suite (Fig. S6). This deletion event was a common feature in the subfamily Cereoideae.

### Contraction and expansion analysis of IR regions

The difference in IR length suggested several expansion/contraction events. To better understand the dynamic changes in IRs, we analyzed the IR boundaries of the 37 Cactaceae plastomes. First, compared with other Caryophyllales plants, all cactus species, including the relatively primitive leafy cactus (*Pe. aculeata*), underwent a contraction event that changed copies of the four-ribosome operon (from two copies to one). This contraction event was a basic feature retained in all other Cactaceae plastomes.

**Fig. 6:**
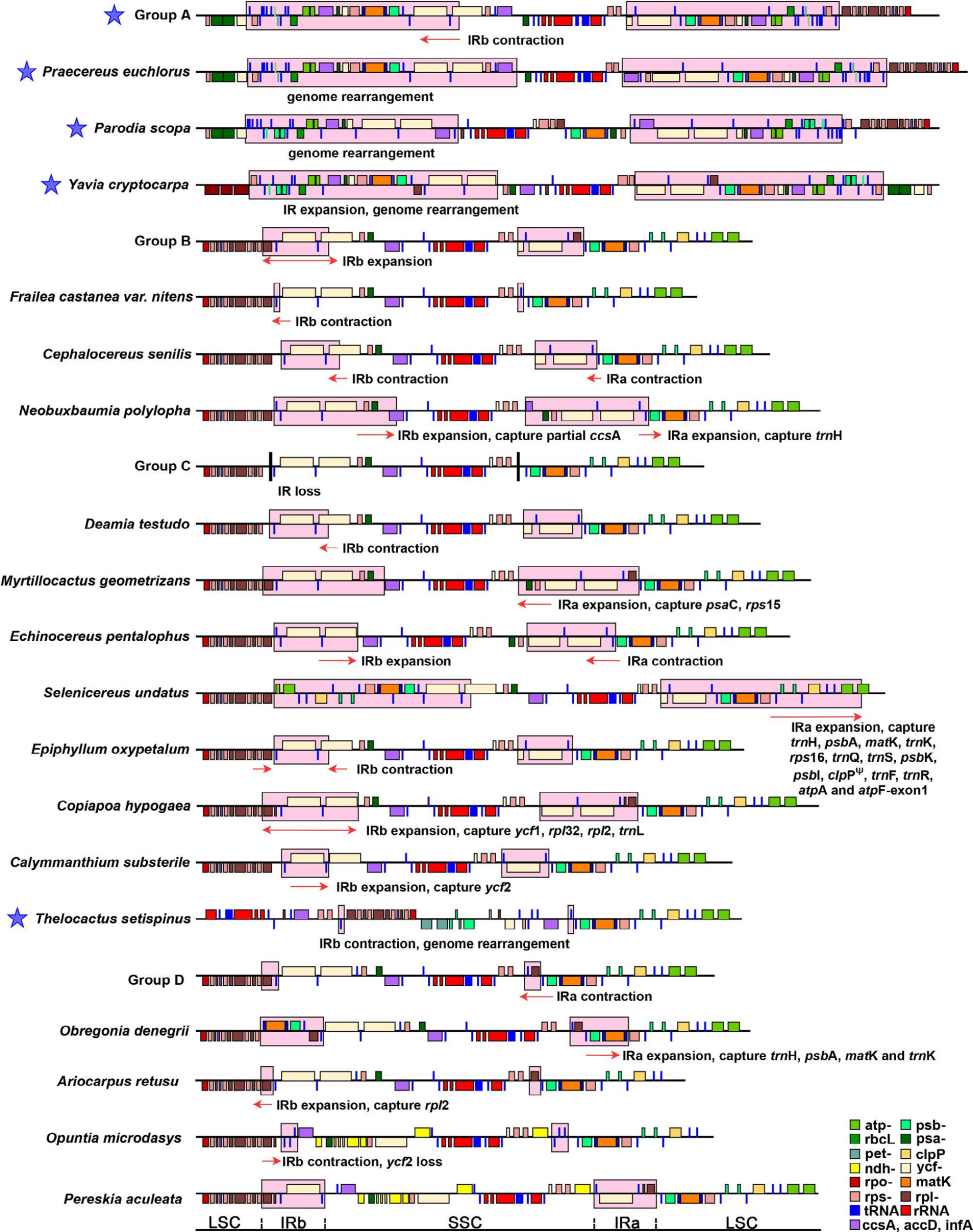
Comparison of the borders among the LSC, SSC, and IR regions of the 37 analyzed plastomes. LSC represents the large single-copy region of the plastome, and SSC represents the small single-copy region of the plastome. IRb and IRa represent the first unit and the last unit of the inverted region, respectively. We highlighted the two IR regions with a pink background color. Different functional groups of genes are drawn in different colors, and their legends are shown in the lower right corner. We use small red arrows to mark the direction of IR region expansion/contraction, and each expansion/contraction is compared from the bottom to the top. A blue five-pointed star on the left side of the Latin name marks the recombination event in the plastome

Subsequently, the IR boundaries further fluctuated in the subfamily Cereoideae. First, in tribe Cacteae, the IRb/SSC border was further contracted into the intergenic region of *rpl2*-*trnM* or *trnM*-*ycf2*. In this case, the IR region was depleted to less than 2 kb and contained only one or two gene(s) (i.e., *rpl2* and/or *trnM*). *Obregonia denegrii* Frič was an exception. After the IRb contracted to *trnM*-*ycf2*, an expansion occurred in IRa, extending to the intergenic region of *trnK*-*trnS*. Four new genes were included in the IRs, i.e., *trnH*, *psbA*, *matK*, and *trnK* (Fig. 6). On the other hand, *T*. *setispinus* was affected by plastome rearrangement, with genes showing differences at several borders. Moreover, dynamic changes in IR boundaries were observed around *ycf1*-*rps15*-*psaC*-*ccsA* in *Co*. *hypogaea*, *Ca. substerile* and tribe Hylocereeae. However, an expansion of IRa/LSC was observed in *Selenicereus*, and the IRs included the new PCGs *psbA*, *matK*, *rps16*, *psbK*, *psbI*, *clpP*, and *atpA* and the first exon of *atpF*. Surprisingly, 437 bp remained in the IR region of *Fr*. *castanea var. nitens*, containing only one gene, *trnM*, which was similar to that of tribe Cacteae. Finally, different IR expansions were observed in tribes Notocacteae and Cereeae. The IR regions were even extended to ~ 30 kb in length, and multiple PCGs were captured by IR regions. In most cases, these genes were in the LSC region, and such large IR expansion events were combined with inversions and rearrangements, resulting in the two copies of several PCGs (Fig. 1).

### Isomeric plastomes

**Fig. 7:**
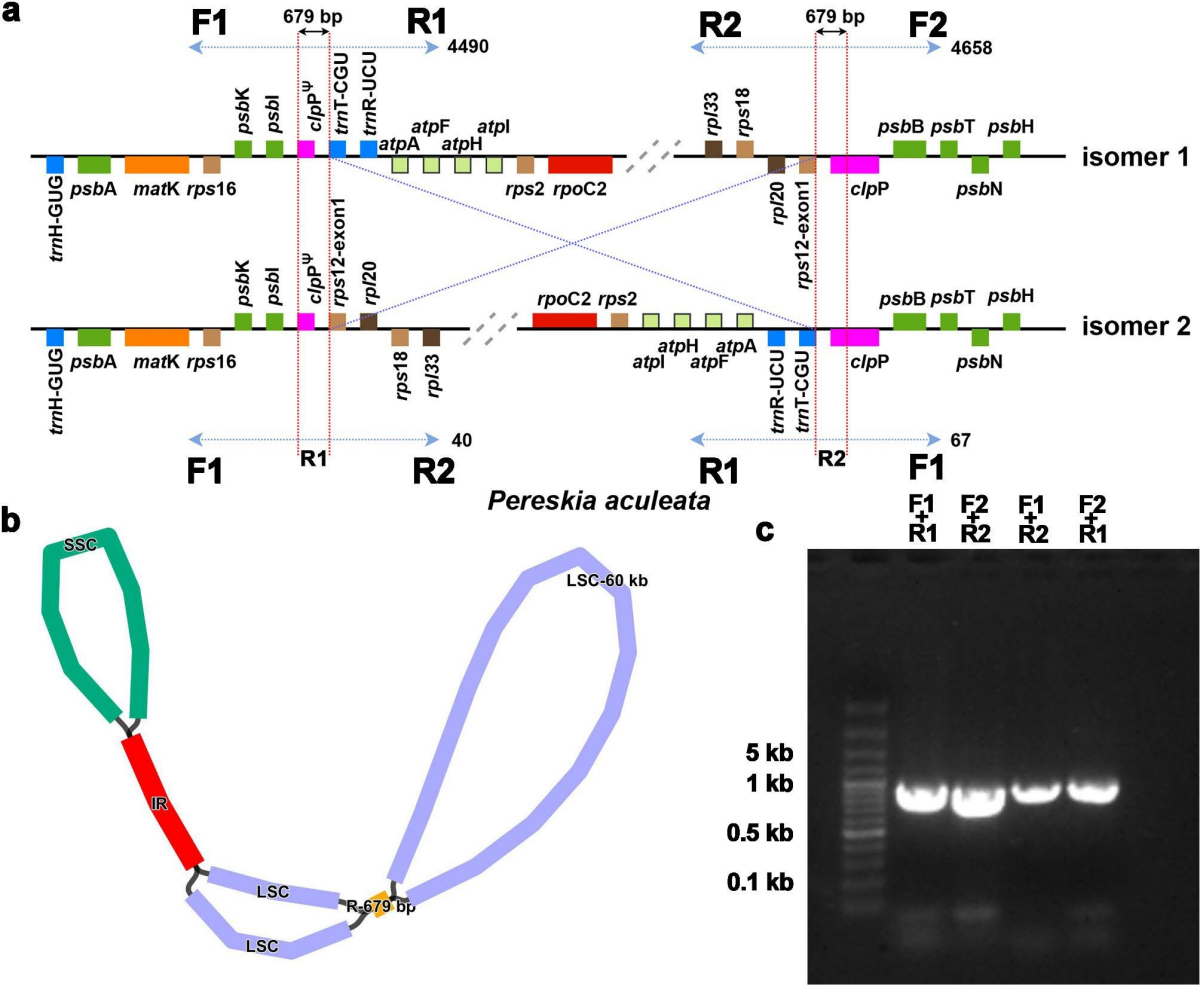
A 60 kb inversion mediated by a pair of short inverted repeats generated isomeric plastomes. **(a)** Diagram of the 60 kb inversion. Isomers 1 and 2 represent preinversion and postinversion plastomes, respectively. In the case of *Pereskia aculeata*, a pair of 679 bp repeats mediated genome inversion. We designed two pairs of primers on both sides of the repeats for F1 + R1 and F2 + R2 to perform PCR expansion experiments. **(b)** Graphic assembly of plastid genomes using GetOrganelle (v1.7.3). In addition to the classic IR region, a pair of short repeat sequences was detected during assembly, which may have mediated a 60 kb inversion of the plastome. **(c)** The two isomers were verified by PCR. We conducted experiments according to the primer pairs designed as shown in Fig. 7a and verified the alternative configuration of the plastome by exchanging primer pairs

To address the problematic isomers encountered during the assembly process, we selected one species (*Pe. aculeata*) for ONT sequencing. With long reads, we confirmed that a pair of inverted repeats with a length of 679 bp were involved in mediating the 60 kb inversion mentioned above, and the two isomers coexisted (Fig. 7a). The repeats contained partial sequences of the *clpP* gene, and this homologous repeat was found in many of our sequenced species (Table S4 and Fig. S2). We also found that the Illumina short reads supported both configurations in these species, which explains why we could assemble plastomes with different configurations (Fig. 7b). In our long-read mapping results, a total of 4,490 and 4,658 long reads supported the classical configuration (i.e., the number of long reads covering the repeat unit and its 1,000 bp flanking region), and only 40 and 67 long reads supported the inverted configuration (isomer 2), suggesting that the frequency of such isomers might be as low as 1%. We also designed primers for PCR amplification experiments. A schematic diagram of the designed primers is shown in Fig. 7a, and the gel electrophoresis diagram is shown in Fig. 7c. Sanger sequencing was performed on the PCR products, and the Sanger reads also supported the existence of the two configurations (Fig. S7). However, for two species (*Op. microdasys* and *Ariocarpus retusus* Scheidw.), we did not detect any evidence of this inverted repeat. It was probably lost after mediating this inversion, and the unusual isomer 2 in the other species was their only configuration.

## Discussion

### Changes in genomic characteristics and gene content

Previous studies have shown that the plastome length of most Caryophyllales except cacti ranges from 151 to 155 kb, and the IR length is generally ~ 25 kb, which is highly consistent with that in other angiosperms [20]. In this study, the 37 Cactaceae plastomes analyzed ranged in size from 110,388 bp to 143,783 bp, which was significantly shorter than other Caryophyllales plastomes. In addition, the IR regions showed multiple intense contraction/expansion events in different cactus lineages compared to other Caryophyllales, and these dynamic changes ultimately affected plastome size. The IR regions were almost completely lost in most lineages of cacti, such as Opuntioideae [22] and the tribe Cacteae assembled here. This phenomenon was also observed in some gymnosperm lineages, such as Taxaceae, Cupressaceae [33, 34], Fabaceae, Geraniaceae, and Orobanchaceae [16–19]. With the evolution of cactus plastomes, the IR region recovered to varying degrees in several clades except for *Fr*. *castanea* var. *nitens*, but this recovery was mainly achieved by occupying the LSC region rather than by incorporating the four ribosomal operons again. Therefore, each plastome still had only four rRNAs, but some of the PCGs and tRNA genes obtained two copies as a result.

Gene annotation indicated common losses or pseudogenization of *ndh* genes in the subfamily Cereoideae, including the *ndh* gene suite (*ndhA* to *ndhK*). Only some plastomes retained a small number of *ndh* genes. Whether *ndh* genes were lost as a whole suite or individually is controversial [35, 36]. Although remnants of genes such as *ndhD*, *ndhB*, and *ndhF* were still recognized in the plastid genome, they were probably nonfunctional genes, which need experimental validation in future studies. In contrast, *Op. microdasys* and *Pe. aculeata* both retained the 11 intact *ndh* genes, suggesting that the losses of *ndh* genes in the cactus family might be specific to Cereoideae lineages. The *ndh* genes encode the thylakoid NADH complex [37, 38], which is closely related to photosynthesis. Meanwhile, *ndh* gene losses/pseudogenization are common in angiosperms [39–42]. These genes may have been transferred horizontally since *ndh* translocations from the plastid to the nuclear genome and mitochondrial genome have been confirmed in *Picea abies* L. Karsch [36].

Compared with the mitochondrial genome, the plastid genome of most angiosperms usually has a complete tRNA transport system containing 30 different tRNA genes [43, 44]. Massive loss of tRNA genes has previously been reported in some parasitic plants [14]; however, this loss is rare in nonparasitic plants. In the cactus family, the loss of *trnV-UAC* has been reported recently in species of the subfamily Cactoideae [45]. Additionally, abnormal loss of three essential tRNA genes (*trnA-UGC*, *trnV-UAC*, and *trnV-GAC*) has previously been found in *Melocactus glaucescens* Buining & Brederoo [46]. This is consistent with the results we report here. Furthermore, we also found that the *trnI-GAU* gene was also lost in one of the 37 cactus species. Based on our richer sampling, *trnV-GAC* was completely lost only in tribe Cereeae. In contrast, the *trnV-UAC* gene is present only in *Pereskia aculeata*. Surprisingly, the loss of *trnA-UGC* was not associated with phylogenetic lineage.

Silva [45] reports that cactus species can import tRNA from the cytosol. Despite the lack of further experimental evidence, this could explain why these cactus species could allow the loss of these essential tRNA genes. This hypothesis has been confirmed in the plant mitochondrial genome [47], where the import of (nuclear-encoded) tRNA genes from the cytosol may gradually replace the tRNA of the organelle itself. According to this hypothesis, the tRNA system of cactus plastomes may still be actively evolving. For example, in the subfamily Cactoideae, the *trnV-UAC* gene is likely entirely dependent on cytosol import, while cytosol import of *trnV-GAC* is specific to the Cereeae tribe. For *trnA-UGC*, the replacement process may still be underway. These results reveal a rapidly evolving tRNA system in the cactus family, which is rare in the rest of the plant kingdom.

The encoded product of the plastidial *accD* gene is a component of the acetyl coenzyme A carboxylase complex [48]. Its product contains a functional carboxyltransferase domain and is highly conserved in most plants, animals and even *E. coli*. Lee [48] reported the existence of five conserved motifs in the *accD* gene, including a potential catalytic site. Despite the loss of upstream sequences of the *accD* gene observed in the cactus plastome, the downstream conserved functional domain is still intact, including all five conserved motifs (Fig. 2). These conserved motifs contain carboxyl-biotin binding sites and putative carboxyltransferase catalytic sites. The retention of these core motifs after extensive rearrangement and sequence loss suggests that *accD* genes in cacti may still be functional. The functional loss of *accD* genes has been reported in the family Campanulaceae, such as in *Platycodon grandiflorum* (Jacq.) A. DC [49] and *Trachelium caeruleum* L. [50]. The plastid-encoded *accD* gene has recently been transferred to its nucleus. However, the nucleus-encoded *accD* transcript is considerably smaller than the plastidic version, consisting of little more than the carboxylase domain of the plastidic *accD* gene fused to a coding region encoding a plastid targeting peptide [50]. In cactus species, the plastid genome also retains a complete carboxylase domain of the *accD* gene, so we speculate that it may still be functional, similarly to the nuclear-encoded *accD* gene above. However, it is not known whether the *accD* gene can be transcribed in the absence of a partial sequence, as the upstream promoter region of the gene is likely to have been lost. In short, there are still many unanswered questions about the *accD* gene in cactus species, and further experiments will be needed to confirm whether it is still functional.

### Phylogenetic relationships in Cactoideae

*B*. *liliputana* is a poikilohydric plant that has morphological and ecological features rarely found in other cacti and was once considered a distinct genus in the tribe Notocacteae. Nyffeler [28, 51, 52] reported the intriguing early-diverging position of *Blossfeldia* within Cactoideae based on molecular phylogeny and thereby initiated a controversial discussion. Crozier established its monophyly and suggested that it should be considered the monogeneric subfamily Blossfeldioideae [53]. As previously reported, *B. liliputana* was the basal clade of Cereoideae with high support in our phylogenetic tree. Previous molecular phylogenies indicated the monophyly of Cacteae [28, 54] as an early-divergent clade in Cactoideae. This view is supported by the present results.

The classification of the genus *Frailea* is controversial. Previously, a morphology-based study placed *Frailea* in the tribe Notocacteae because anatomical studies showed that *Notocactus* and *Frailea* share common features [55]. Nyffeler [28] addressed relationships within Cereoideae using *trnK*/*matK* and *trnL*-*trnF* sequence data, but the phylogenetic position of *Frailea* was still not determined. Hernández-Hernández [26] added two other chloroplast sequences (*matK* and *rpl16*), combined *trnK*/*matK* and *trnL*-*trnF* and suggested the topology {(Blossfeldieae + (Cacteae + (*Calymmanthium* + (*Copiapoa* + (*Frailea* + (Hylocereeae + (Rhipsalideae + (Notocacteae + (Cacteae)))))))))}, which is inconsistent with the phylogenetic tree derived from the present study. The present cpDNA-based phylogenetic tree supports the topology {(Blossfeldieae + (Cacteae + (*Calymmanthium* + (*Copiapoa* + (Hylocereeae + (*Frailea* + (Rhipsalideae + (Notocacteae + (Cacteae))))))))))}. The genus *Frailea* does not appear to be closely related to *Parodia*, with the latter recognized as a core Notocacteae taxon. However, as our study is based on one sample of *Frailea*, future studies including wider sampling should be carried out across the tribe Notocacteae and the genus *Frailea* to further test the relationships reported in the present study.

### Plastomic structural changes concomitant with Cactoideae evolution

In cacti, plastome rearrangement is ubiquitous. Small-fragment rearrangements in the LSC region of the plastome have previously been observed in several genera of the order Caryophyllales, such as *Chenopodium* [56]. This rearrangement includes an ~ 6 kb inversion involving four genes, i.e., *rbcL*-*atpB*-*atpE*-*trnM*, which has been observed in most cactus species with a few exceptions. Furthermore, many unique inversions and rearrangements have been observed in cacti. For example, rearrangements of the SSC region have been observed, concomitant with fragment deletions involving the *ndh* gene suite in the subfamily Cereoideae.

However, the most striking is a large inversion of approximately 60 kb that seems to be associated with a pair of short-inverted repeats. Although this structure has not been detected in all plastomes studied, it is present in most species (20 out of 23) except those whose plastomes that have undergone major recombination. It has been confirmed to be an important mechanism [57] for mediating intramolecular recombination in *Pe. aculeata*. The mechanism that affects the generation of rearrangements is not unique to cacti. The intramolecular recombination mediated by these repeated structures has been confirmed in other lineages, e.g., Cupressoideae [58] and Asteraceae [59].

Surprisingly, as the cactus evolved, the plastomes continued to undergo rearrangement. The most complex rearrangements in Caryophyllales have been observed in tribe Notocacteae and Cereeae species, involving several large inversions and rearrangements, highly complicated by the expansion of the IR region. These genome rearrangement events involved almost the entire plastome and completely changed the gene order compared to that in the original genomes. This atypical plastome configuration has not been observed in Caryophyllales before and is also rare in other angiosperms. Several small fragments were exchanged among the plastomes and were fixed as the species diverged. These results suggest that the Cereoideae plastomes, especially for *Pa*. *scopa* and Cereeae, might have undergone more mutational events during evolution than those of other Caryophyllales plants.

Dynamic changes in the IR region have been observed in many plant lineages, including extreme IR losses, IR expansions, and even complete disappearance (e.g., the IRLC legumes) [60]. These changes had significant effects on plastome size and the number of plastid genes. We observed multiple IR expansion/contraction events in the subfamily Cereoideae, which seem to have originated independently. These include extreme losses in the tribe Cacteae and a large IRa/LSC expansion event observed in the genus *Selenicereus*, *Pa*. *scopa*, and the Cereeae tribe. The Cacteae plastomes shared the same contraction event; however, the expansions in *Selenicereus* did not share a common ancestor with those in tribe Notocacteae and Cereeae. *Fr*. *castanea* var. *nitens* had the characteristics of an IR boundary similar to that in Cacteae, including only one gene (*trnM*) captured by IRs without any IR expansion. However, phylogenetic analysis based on plastome data unambiguously suggested that they are two different taxa.

## Conclusion

In the present study, we systematically show the unique evolution of Cactaceae plastomes, including frequent plastome inversions and rearrangements, as well as other evolutionary features such as IR expansions/losses and gene losses, which greatly promoted the formation of plastome variations. These complex evolutionary phenomena might be related to adaptation to extreme environments and suggest that the Cereoideae lineages have undergone more mutation events than other cactus lineages. Although these results are limited by material sampling, our results provide new insights into the evolutionary history of the plastome in Cereoideae compared to other angiosperms.

## Materials and methods

### Plant material

To avoid resource waste caused by repeated sampling, we accessed the European Nucleotide Archive (ENA) (https://www.ncbi.nlm.nih.gov/sra) database and obtained WGS (whole-genome sequencing) data for 9 species with a complete plastome assembly. They all come from the Kew Tree of Life project [61], which is freely available for use. Furthermore, we collected fresh stem samples of 26 cactuses from flower markets in Chongqing, Guangxi, and Fujian in September 2020. All specimens were deposited in the Herbarium of Southwest University, Chongqing. Two previously reported species (*Carnegiea gigantea*, NC_027618.1; *Lophocereus schottii*, NC_041727.1) were also included in our analysis. The details of the plant samples used for plastome assembly are given in Table S5.

### DNA extraction and plastome assembly

Total genomic DNA was extracted by using the CTAB method [62]. The DNA library with an insert size of 350 bp was constructed using an NEBNext® library construction kit (supplier, city country) and sequenced by using the HiSeq XTen PE150 sequencing platform (supplier, city country). See Table S6 for detailed information on Illumina sequencing data quality. Furthermore, clean data were obtained by using Trimmomatic (v0.32) [63] as follows: we removed low-quality sequences, including sequences with a quality value of Q < 19 that accounted for more than 50% of the total bases and sequences in which more than 5% of the bases were “N”. To assemble cactus plastomes, de novo genome assembly from the clean data was accomplished utilizing GetOrganelle (v1.7.3) [64] with the default setting. For linear contigs, NOVOPlasty (v3.8.1) [65] was used for further contig extensions. The correctness of the assembly was confirmed by using Bowtie2 (v2. 0.1) [66] to manually edit and map all the raw reads to the assembled genome sequence under the default settings. Detailed assembly information is shown in Table S7. *Pereskia aculeata* was also sequenced using the Oxford Nanopore promethION platform.

We assembled the draft mitochondrial genome using Illumina reads with the ‘embplant_mt’ option in GetOrganelle (v1.7.3). Then, we visualized the raw GFA file produced by GetOrganelle (v1.7.3) in Bandage (v0.8.1) [67]. The plastid/nuclear-derived contigs were removed manually based on the coverage and BLASTn results retrieved from the NCBI database. Only the mitogenomes that consisted of a network of closed and connected contigs were thought to be complete. Although the extensive repeats could not be resolved without long reads, the draft mitogenome assembled here was considered complete, and it represents all mitochondrial DNA sequences of the species.

### Genome annotation

The plastomes were initially annotated by using GeSeq [68] with two reference genomes (*Carnegiea gigantea*, GenBank: NC_027618.1 and *Lophocereus schottii*, GenBank: NC_041727.1). Subsequently, annotations with problems were manually edited by using Apollo [69]. To further confirm the presence or absence of genes, we used the 80 unique protein-coding genes (PCGs) and the 30 unique tRNA genes annotated in *Portulaca oleracea* as query sequences to search for homologous sequences using the BLASTn program [70]. The parameters were as follows: -evalue 1e-5, -word_size 9, -gapopen 5, - gapextend 2, -reward 2, -penalty − 3, and -dust no. If only a partial sequence of the gene was identified in each genome, this gene was considered a pseudogene. However, for genes whose conserved functional domains still exist, such as *accD*, further experiments are still needed for confirmation. If a premature termination codon was encountered in the coding sequence, we also considered it to be a pseudogene, although we cannot rule out the possibility of an RNA editing event for correction.

### Repeat element analysis

We used ROUSFinder [71] to analyze the repeating elements of each plastome. The minimum repeat unit length was set to 30 bp, with the remaining parameters set to the default. ROUSFinder was originally developed for the identification of nontandem repeats in plant mitochondrial genomes. This program could also be used to eliminate redundant and overlapping repeat units in highly repetitive plastomes.

### Alignment and phylogenetic inference

In addition to our 37 plastomes, one additional related-species dataset (i.e., *Blossfeldia liliputana*, accession numbers listed in Table S8) was downloaded from the NCBI and used for the construction of phylogenetic trees. Considering the rearrangement of plastomes, we used conserved PCGs to construct phylogenetic trees. A total of 57 orthologous genes among the species analyzed were identified and extracted by using PhyloSuite (v1.2.1) [72]. The corresponding nucleotide sequences were aligned by using MAFFT (v7.450) [73] implemented in PhyloSuite. These aligned nucleotide sequences were concatenated (the alignment of consensus sequences included 43,470 nucleotide sites) and used to construct the phylogenetic trees by the maximum likelihood (ML) method implemented in IQ-TREE (v2.0) [74]. The parameters were “iqtree2 -s example.phy --alrt 1000 -B 1000”. Bootstrap analysis was performed with 1,000 replicates. Bayesian inference (BI) analysis was performed in MrBayes (v3.2.6) [75] using the Markov Chain Monte Carlo method with 200,000 generations and sampling of trees every 100 generations. The first 20% of trees were discarded as burn-in, with the remaining trees used to generate a consensus tree.

### Comparative analysis of plastomes

Whole-genome alignments were performed to examine the arrangement of locally colinear blocks (LCBs) of different cactus plastome structural types using progressiveMauve (v2.3.1) [76] with default parameters. Before this, we manually removed the last IR region of plastomes. The plastome syntenies were plotted using Mcscan (v.2) [77] implemented in TBtools (v.1.106) [78]. Specifically, we first obtained BLASTn results between the pairs of plastomes (IR removed), and the e-value was set as 1*e*-5. Then, the alignments were split into 10 bp fragments, which were forced to be used as a ‘gene’ in TBtools (v.1.106). The dot plots of plastomes were drawn using MAFFT online version [79] or Gepard (v1.40) [80]. Boundary changes in the IR regions were drawn manually.

### Identification of isomeric plastomes

To address the challenges posed by repeat sequences in Illumina-based data assembly, we selected a species with a pair of typical short repeat sequences, *Pereskia aculeata*, for ONT sequencing. Then, repeat regions were solved based on long-read sequences. To put it simply, we treated the assembled genome as the reference configuration and the inverted genome mediated by repeated sequences as potential isomers. A schematic diagram of the two different isomers is shown in Fig. 7a. For the two isomers, we extracted the repeat sequence and its 1,000 bp flanking regions (shown as a blue dashed arrow in Fig. 7a) and mapped the long reads to the extracted sequences. There were two repeating units for each isomer, and the average number of long reads was calculated. Only long reads spanning the repeats and their flanking regions of at least 1,000 bp were considered to support this isomer.

The primer sequences for PCRs were designed (Fig. 7a) and are listed in Table S9. PCRs were performed in a 25 µl mixture, including 1 µl total DNA (20 ng/µl), 0.5 µl each of the forward and reverse primers (10 µmol/l), 13 µl Tiangen 2× Taq PCR MasterMix, and 10 µl ddH2O. After an initial denaturation step at 94 °C for 2 min, PCRs were conducted for 35 cycles. Each cycle included denaturation at 94 °C for 30 s, annealing at 55 °C for 30 s, and elongation at 72 °C for 1 min.

## Electronic supplementary material

Below is the link to the electronic supplementary material.

Supplementary Material 1

Supplementary Material 2

Supplementary Material 3

Supplementary Material 4

Supplementary Material 5

## Data Availability

The raw sequencing data generated in this study and the plastid genomes were deposited in the NCBI database (https://www.ncbi.nlm.nih.gov/) with accession numbers PRJNA715621 and MW553043-MW553073. The plastome sequences can also be found in Figshare (https://figshare.com/articles/media/DataS1/21152380). All the samples were deposited at the Herbarium of Southwest University, Chongqing, China. All other data and material generated in this manuscript are available by contacting Lijingling1997@163.com.

## Abbreviations

CAM: Crassulacean acid metabolism
WGD: Whole-genome duplication
SC: single-copy
IR: inverted repeat
LSC: large single-copy
SSC: small single-copy
PCGs: Protein-coding genes
BLAST: Basic Local Alignment Search Tool
ONT: Oxford Nanopore Technologies
rRNA: ribosomal RNA
ML: Maximum Likelihood
BI: Bayesian Inference
LCB: Locally Colinear Block

## References

[1] Abouseadaa HH, Atia MAM, Younis IY, Issa MY, Ashour HA, Saleh I, Osman GH, Arif IA, Mohsen E (2020). Gene-targeted molecular phylogeny, phytochemical profiling, and antioxidant activity of nine species belonging to family Cactaceae. Saudi J Biol Sci.

[2] Novoa A, Le Roux JJ, Robertson MP, Wilson JR, Richardson DM (2014). Introduced and invasive cactus species: a global review. AoB PLANTS.

[3] James DM (2016). Biogeography and Biodiversity of Cacti. Cactus and Succulent Journal.

[4] Oulo MA, Yang J-X, Dong X, Wanga VO, Mkala EM, Munyao JN, Onjolo VO, Rono PC, Hu G-W, Wang Q-F (2020). Complete chloroplast genome of Rhipsalis baccifera, the only Cactus with natural distribution in the Old World: genome rearrangement, Intron Gain and loss, and implications for phylogenetic studies. Plants.

[5] Ritz CM, Reiker J, Charles G, Hoxey P, Hunt D, Lowry M, Stuppy W, Taylor N (2012). Molecular phylogeny and character evolution in terete-stemmed Andean opuntias (Cactaceae-Opuntioideae). Mol Phylogenet Evol.

[6] Edwards EJ, Diaz M (2006). Ecological physiology of Pereskia guamacho, a cactus with leaves. Plant Cell Environ.

[7] Nobel PS (1977). Water relations and photosynthesis of a barrel cactus, Ferocactus acanthodes, in the Colorado desert. Oecologia.

[8] North GB, Nobel PS (1996). Radial hydraulic conductivity of individual Root tissues of Opuntia ficus-indica (L.) Miller as Soil moisture varies. Ann Botany.

[9] Edwards EJ, Ogburn RM (2012). Angiosperm responses to a Low-CO2World: CAM and C4Photosynthesis as parallel evolutionary trajectories. Int J Plant Sci.

[10] Sutton BG (1981). Carbohydrate metabolism of cactus in a desert environment. Plant Physiol.

[11] Wang N, Yang Y, Moore MJ, Brockington SF, Walker JF, Brown JW, Liang B, Feng T, Edwards C, Mikenas J (2019). Evolution of Portulacineae marked by Gene Tree Conflict and Gene Family Expansion Associated with Adaptation to Harsh environments. Mol Biol Evol.

[12] Copetti D, Búrquez A, Bustamante E, Charboneau JLM, Childs KL, Eguiarte LE, Lee S, Liu TL, McMahon MM, Whiteman NK (2017). Extensive gene tree discordance and hemiplasy shaped the genomes of north american columnar cacti. Proc Natl Acad Sci USA.

[13] Zheng J, Meinhardt LW, Goenaga R, Zhang D, Yin Y (2021). The chromosome-level genome of dragon fruit reveals whole-genome duplication and chromosomal co-localization of betacyanin biosynthetic genes. Hortic Res.

[14] Braukmann T, Kuzmina M, Stefanovic S (2013). Plastid genome evolution across the genus Cuscuta (Convolvulaceae): two clades within subgenus Grammica exhibit extensive gene loss. J Exp Bot.

[15] Park J, Suh Y, Kim S (2020). A complete chloroplast genome sequence of Gastrodia elata (Orchidaceae) represents high sequence variation in the species. Mitochondrial DNA Part B Resources.

[16] Bogdanova VS, Mglinets AV, Shatskaya NV, Kosterin OE, Solovyev VI, Vasiliev GV (2018). Cryptic divergences in the genus Pisum L. (peas), as revealed by phylogenetic analysis of plastid genomes. Mol Phylogenet Evol.

[17] Chris Blazier J, Guisinger MM, Jansen RK (2011). Recent loss of plastid-encoded ndh genes within Erodium (Geraniaceae). Plant Mol Biol.

[18] Li X, Zhang TC, Qiao Q, Ren Z, Zhao J, Yonezawa T, Hasegawa M, Crabbe MJ, Li J, Zhong Y (2013). Complete chloroplast genome sequence of holoparasite Cistanche deserticola (Orobanchaceae) reveals gene loss and horizontal gene transfer from its host Haloxylon ammodendron (Chenopodiaceae). PLoS ONE.

[19] Sanderson MJ, Copetti D, Búrquez A, Bustamante E, Charboneau JL, Eguiarte LE, Kumar S, Lee HO, Lee J, McMahon M (2015). Exceptional reduction of the plastid genome of saguaro cactus (Carnegiea gigantea): loss of the ndh gene suite and inverted repeat. Am J Bot.

[20] Yao G, Jin JJ, Li HT, Yang JB, Mandala VS, Croley M, Mostow R, Douglas NA, Chase MW, Christenhusz MJM (2019). Plastid phylogenomic insights into the evolution of Caryophyllales. Mol Phylogenet Evol.

[21] Downie S, Palmer J (1994). A chloroplast DNA phylogeny of the Caryophyllales based on structural and inverted repeat restriction site variation. Syst Bot.

[22] Köhler M, Reginato M, Souza-Chies TT, Majure LC (2020). Insights into Chloroplast Genome Evolution Across Opuntioideae (Cactaceae) reveals robust yet sometimes conflicting phylogenetic topologies. Front Plant Sci.

[23] Edwards EJ, Nyffeler R, Donoghue MJ (2005). Basal cactus phylogeny: implications of Pereskia (Cactaceae) paraphyly for the transition to the cactus life form. Am J Bot.

[24] Taylor NP (2005). MAIHUENIA POEPPIGII: Cactaceae. Curtis’s Bot Magazine.

[25] Mayta L, Molinari-Novoa E (2015). L’intégration du genre Leuenbergeria Lodé dans sa propre sous-famille. Leuenbergerioideae Mayta & Mol Nov subfam nov Succulentopi.

[26] Hernández-Hernández T, Hernández HM, De-Nova JA, Puente R, Eguiarte LE, Magallón S (2011). Phylogenetic relationships and evolution of growth form in Cactaceae (Caryophyllales, Eudicotyledoneae). Am J Bot.

[27] Butterworth C (2009). Resolving “Nyffeler’s Puzzle”– the intriguing taxonomic position of Blossfeldia. Haseltonia.

[28] Nyffeler R (2002). Phylogenetic relationships in the cactus family (Cactaceae) based on evidence from trnK/ matK and trnl-trnf sequences. Am J Bot.

[29] Arias S, Terrazas T, Arreola-Nava HJ, Vázquez-Sánchez M, Cameron KM (2005). Phylogenetic relationships in Peniocereus (Cactaceae) inferred from plastid DNA sequence data. J Plant Res.

[30] Ritz CM, Martins L, Mecklenburg R, Goremykin V, Hellwig FH (2007). The molecular phylogeny of Rebutia (Cactaceae) and its allies demonstrates the influence of paleogeography on the evolution of South American mountain cacti. Am J Bot.

[31] Guerrero PC, Majure LC, Cornejo-Romero A, Hernández-Hernández T (2019). Phylogenetic Relationships and Evolutionary Trends in the Cactus Family. J heredity.

[32] Qin Q, Li J, Zeng S, Xu Y, Han F, Yu J (2022). The complete plastomes of red fleshed pitaya (Selenicereus monacanthus) and three related Selenicereus species: insights into gene losses, inverted repeat expansions and phylogenomic implications. Physiol Mol biology plants: Int J Funct plant biology.

[33] Duan H, Guo J, Xuan L, Wang Z, Li M, Yin Y, Yang Y (2020). Comparative chloroplast genomics of the genus Taxodium. BMC Genomics.

[34] Yi X, Gao L, Wang B, Su YJ, Wang T (2013). The complete chloroplast genome sequence of Cephalotaxus oliveri (Cephalotaxaceae): evolutionary comparison of cephalotaxus chloroplast DNAs and insights into the loss of inverted repeat copies in gymnosperms. Genome Biol Evol.

[35] Krause K (2008). From chloroplasts to “cryptic” plastids: evolution of plastid genomes in parasitic plants. Curr Genet.

[36] Ranade SS, García-Gil MR, Rosselló JA (2016). Non-functional plastid ndh gene fragments are present in the nuclear genome of Norway spruce (Picea abies L. Karsch): insights from in silico analysis of nuclear and organellar genomes. Mol Genet genomics: MGG.

[37] Sazanov LA, Burrows PA, Nixon PJ (1998). The plastid ndh genes code for an NADH-specific dehydrogenase: isolation of a complex I analogue from pea thylakoid membranes. Proc Natl Acad Sci USA.

[38] Strand DD, D’Andrea L, Bock R (2019). The plastid NAD(P)H dehydrogenase-like complex: structure, function and evolutionary dynamics. Biochem J.

[39] Kim HT, Kim JS, Moore MJ, Neubig KM, Williams NH, Whitten WM, Kim JH (2015). Seven new complete plastome sequences reveal Rampant Independent loss of the ndh Gene Family across Orchids and Associated instability of the inverted Repeat/Small Single-Copy Region Boundaries. PLoS ONE.

[40] Lin CS, Chen JJW, Chiu CC, Hsiao HCW, Yang CJ, Jin XH, Leebens-Mack J, de Pamphilis CW, Huang YT, Yang LH (2017). Concomitant loss of NDH complex-related genes within chloroplast and nuclear genomes in some orchids. The Plant journal: for cell and molecular biology.

[41] Ni Z, Ye Y, Bai T, Xu M, Xu LA (2017). Complete chloroplast genome of Pinus massoniana (Pinaceae): gene rearrangements, loss of ndh genes, and short inverted repeats contraction, expansion. Molecules.

[42] Sun Y, Moore MJ, Lin N, Adelalu KF, Meng A, Jian S, Yang L, Li J, Wang H (2017). Complete plastome sequencing of both living species of Circaeasteraceae (Ranunculales) reveals unusual rearrangements and the loss of the ndh gene family. BMC Genomics.

[43] Alkatib S, Fleischmann TT, Scharff LB, Bock R (2012). Evolutionary constraints on the plastid tRNA set decoding methionine and isoleucine. Nucleic Acids Res.

[44] Mandal D, Köhrer C, Su D, Babu IR, Chan CT, Liu Y, Söll D, Blum P, Kuwahara M, Dedon PC (2014). Identification and codon reading properties of 5-cyanomethyl uridine, a new modified nucleoside found in the anticodon wobble position of mutant haloarchaeal isoleucine tRNAs. RNA.

[45] Morais da Silva G, de Santana Lopes A, Gomes Pacheco T, Lima de Godoy Machado K, Silva MC, de Oliveira JD, de Baura VA, Balsanelli E, Maltempi de Souza E, de Oliveira Pedrosa F (2021). Genetic and evolutionary analyses of plastomes of the subfamily Cactoideae (Cactaceae) indicate relaxed protein biosynthesis and tRNA import from cytosol. Brazilian J Bot.

[46] Dalla Costa TP, Silva MC, de Santana Lopes A, Gomes Pacheco T, de Oliveira JD, de Baura VA, Balsanelli E, Maltempi de Souza E, de Oliveira Pedrosa F, Rogalski M (2022). The plastome of Melocactus glaucescens Buining & Brederoo reveals unique evolutionary features and loss of essential tRNA genes. Planta.

[47] Warren JM, Salinas-Giegé T, Triant DA, Taylor DR, Drouard L, Sloan DB (2021). Rapid shifts in mitochondrial tRNA import in a plant lineage with extensive mitochondrial tRNA gene loss. Mol Biol Evol.

[48] Lee SS, Jeong WJ, Bae JM, Bang JW, Liu JR, Harn CH (2004). Characterization of the plastid-encoded carboxyltransferase subunit (accD) gene of potato. Mol Cells.

[49] Hong CP, Park J, Lee Y, Lee M, Park SG, Uhm Y, Lee J, Kim CK (2017). accD nuclear transfer of Platycodon grandiflorum and the plastid of early Campanulaceae. BMC Genomics.

[50] Rousseau-Gueutin M, Huang X, Higginson E, Ayliffe M, Day A, Timmis JN (2013). Potential functional replacement of the plastidic acetyl-CoA carboxylase subunit (accD) gene by recent transfers to the nucleus in some angiosperm lineages. Plant Physiol.

[51] Anderson EF. The Cactus Family: Timber Press, Portland, Oregon, USA.; 2001.

[52] Gibson A, Nobel P (1986). The Cactus primer.

[53] Root G (2004). Resolving the phylogenetic placement of Blossfeldia liliputana (Cactaceae): reticulate evolution, chloroplast inheritance, and graft-chimeras. Bradleya.

[54] Butterworth C, Cota-Sanchez J, Wallace R (2002). Molecular systematics of Tribe Cacteae (Cactaceae: Cactoideae): a phylogeny based on rpl16 intron sequence variation. Syst Bot.

[55] Eggli U, Nyffeler R. (1352) Proposal to conserve the name Parodia against Frailea (Cactaceae). *TAXON* 1998, 47(2):475–476.

[56] Hong SY, Cheon KS, Yoo KO, Lee HO, Cho KS, Suh JT, Kim SJ, Nam JH, Sohn HB, Kim YH (2017). Complete chloroplast genome sequences and comparative analysis of Chenopodium quinoa and C. album. Front Plant Sci.

[57] Ruhlman TA, Zhang J, Blazier JC, Sabir JSM, Jansen RK (2017). Recombination-dependent replication and gene conversion homogenize repeat sequences and diversify plastid genome structure. Am J Bot.

[58] Qu XJ, Wu CS, Chaw SM, Yi TS (2017). Insights into the existence of Isomeric Plastomes in Cupressoideae (Cupressaceae). Genome Biol Evol.

[59] Kim KJ, Choi KS, Jansen RK (2005). Two chloroplast DNA inversions originated simultaneously during the early evolution of the sunflower family (Asteraceae). Mol Biol Evol.

[60] Sveinsson S, Cronk Q. Conserved gene clusters in the scrambled plastomes of IRLC legumes (Fabaceae: Trifolieae and Fabeae). bioRxiv 2016:040188.

[61] Baker WJ, Bailey P, Barber V, Barker A, Bellot S, Bishop D, Botigué LR, Brewer G, Carruthers T, Clarkson JJ (2022). A Comprehensive Phylogenomic platform for exploring the Angiosperm Tree of Life. Syst Biol.

[62] Arseneau JR, Steeves R, Laflamme M (2017). Modified low-salt CTAB extraction of high-quality DNA from contaminant-rich tissues. Mol Ecol Resour.

[63] Bolger AM, Lohse M, Usadel B (2014). Trimmomatic: a flexible trimmer for Illumina sequence data. Bioinf (Oxford England).

[64] Jin JJ, Yu WB, Yang JB, Song Y, dePamphilis CW, Yi TS, Li DZ (2020). GetOrganelle: a fast and versatile toolkit for accurate de novo assembly of organelle genomes. Genome Biol.

[65] Dierckxsens N, Mardulyn P, Smits G (2017). NOVOPlasty: de novo assembly of organelle genomes from whole genome data. Nucleic Acids Res.

[66] Langmead B, Trapnell C, Pop M, Salzberg SL (2009). Ultrafast and memory-efficient alignment of short DNA sequences to the human genome. Genome Biol.

[67] Wick RR, Schultz MB, Zobel J, Holt KE (2015). Bandage: interactive visualization of de novo genome assemblies. Bioinf (Oxford England).

[68] Tillich M, Lehwark P, Pellizzer T, Ulbricht-Jones ES, Fischer A, Bock R, Greiner S (2017). GeSeq - versatile and accurate annotation of organelle genomes. Nucleic Acids Res.

[69] Misra S, Harris N. Using Apollo to Browse and Edit Genome Annotations. *Current Protocols in Bioinformatics* 2005, 12(1):9.5.1–9.5.28. 10.1002/0471250953.bi0905s12 18428771

[70] Chen Y, Ye W, Zhang Y, Xu Y (2015). High speed BLASTN: an accelerated MegaBLAST search tool. Nucleic Acids Res.

[71] Wynn EL, Christensen AC (2019). Repeats of unusual size in Plant mitochondrial genomes: identification, incidence and evolution. G3 (Bethesda).

[72] Zhang D, Gao F, Jakovlic I, Zou H, Zhang J, Li WX, Wang GT (2020). PhyloSuite: an integrated and scalable desktop platform for streamlined molecular sequence data management and evolutionary phylogenetics studies. Mol Ecol Resour.

[73] Rozewicki J, Li S, Amada KM, Standley DM, Katoh K (2019). MAFFT-DASH: integrated protein sequence and structural alignment. Nucleic Acids Res.

[74] Minh BQ, Schmidt HA, Chernomor O, Schrempf D, Woodhams MD, von Haeseler A, Lanfear R (2020). IQ-TREE 2: New Models and efficient methods for phylogenetic inference in the genomic era. Mol Biol Evol.

[75] Ronquist F, Teslenko M, van der Mark P, Ayres DL, Darling A, Höhna S, Larget B, Liu L, Suchard MA, Huelsenbeck JP (2012). MrBayes 3.2: efficient bayesian phylogenetic inference and model choice across a large model space. Syst Biol.

[76] Darling AE, Mau B, Perna NT (2010). progressiveMauve: multiple genome alignment with gene gain, loss and rearrangement. PLoS ONE.

[77] Wang Y, Tang H, Debarry JD, Tan X, Li J, Wang X, Lee TH, Jin H, Marler B, Guo H (2012). MCScanX: a toolkit for detection and evolutionary analysis of gene synteny and collinearity. Nucleic Acids Res.

[78] Chen C, Chen H, Zhang Y, Thomas HR, Frank MH, He Y, Xia R (2020). TBtools: an integrative Toolkit developed for interactive analyses of big Biological Data. Mol Plant.

[79] Katoh K, Rozewicki J, Yamada KD (2019). MAFFT online service: multiple sequence alignment, interactive sequence choice and visualization. Brief Bioinform.

[80] Krumsiek J, Arnold R, Rattei T (2007). Gepard: a rapid and sensitive tool for creating dotplots on genome scale. Bioinf (Oxford England).

