# Supplementary material for: Plastome variations reveal the distinct evolutionary scenarios of plastomes in the subfamily Cereoideae (Cactaceae)
Source: BMC Plant Biol. 2023 Mar 8;23:132. doi: 10.1186/s12870-023-04148-4 (PMC9993602; doi:10.1186/s12870-023-04148-4)
Supplement: Supplementary file 1 — Supplementary Material 1 [file 12870_2023_4148_MOESM1_ESM.pdf]

Figure S1.Plastid genome map of 35 cacti species. The genes outside the circle are transcribed in the counterclockwise direction, and the genes inside the circle are transcribed in the clockwise direction. Different colors in genes represent different functions. The dark gray area and light gray area of the inner circle represent the ratio of GC content to AT content in the genome, respectively.

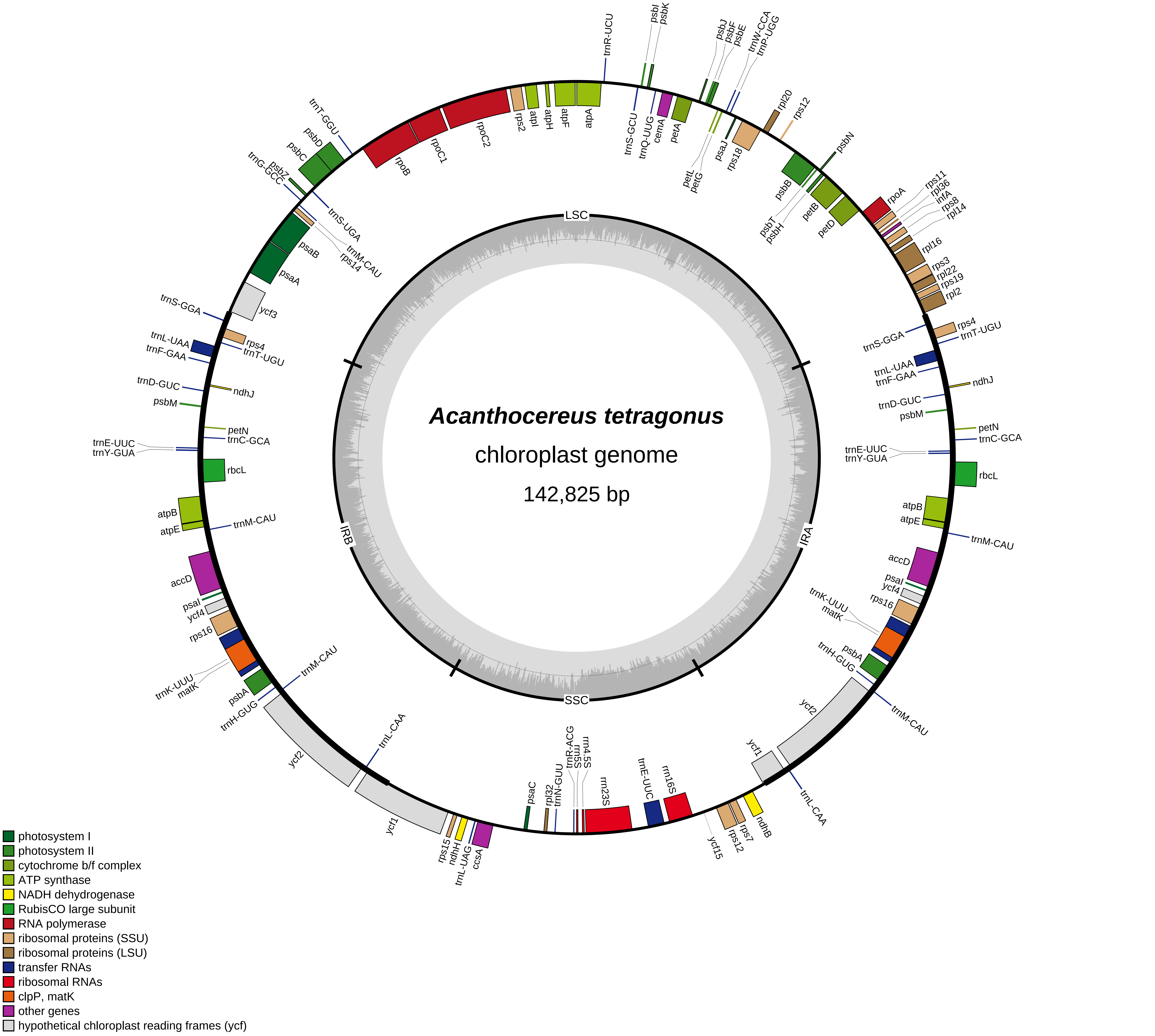

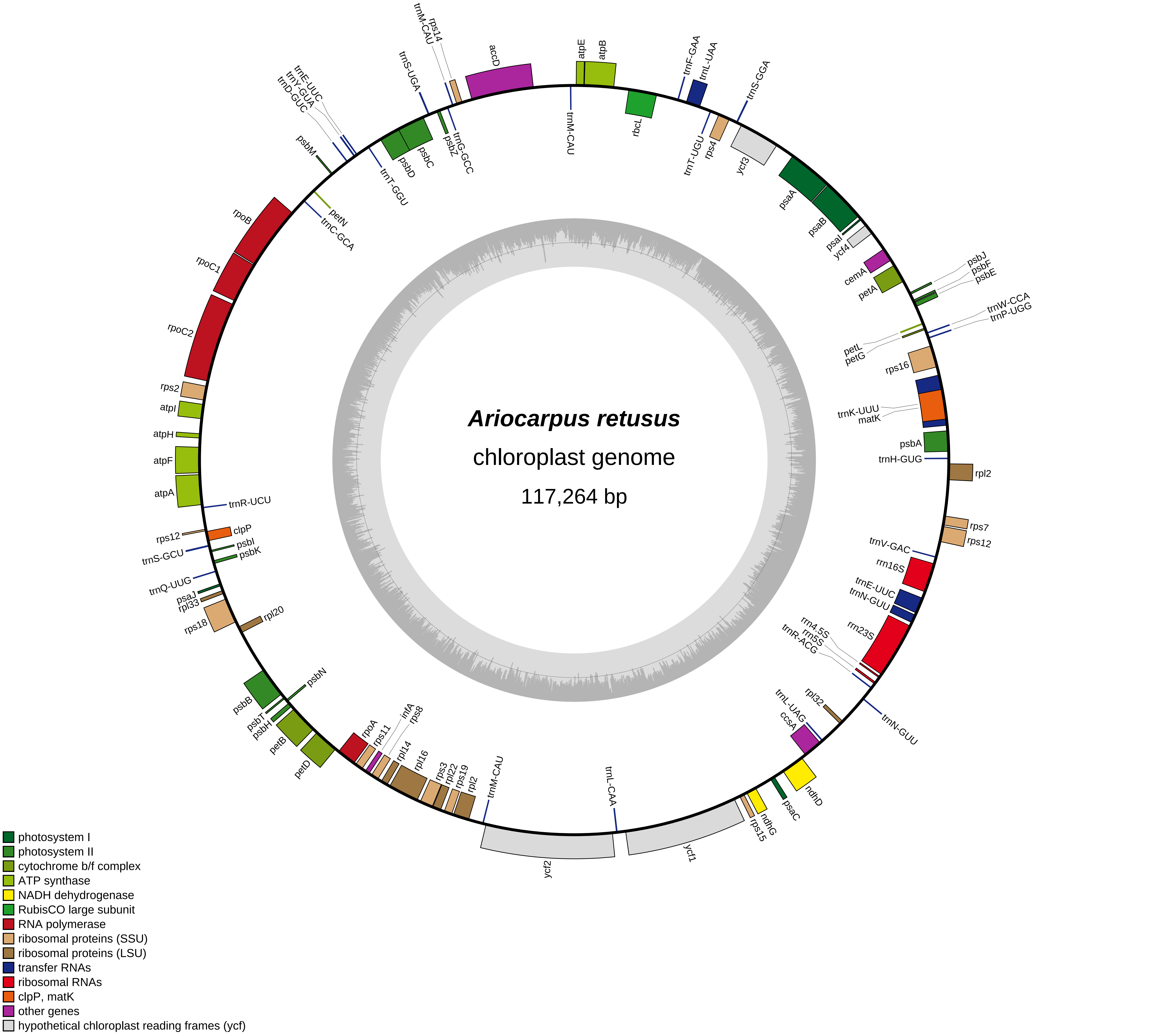

Ariocarpus retusus
chloroplast genome
117,264 bp
trnM-CAU
atpE
atpB
trnF-GAA
trnL-UAA
trnS-GGA
rbcL
trnT-UGU
rps4
ycf3
psaA
psaB
psaI
ycf4
cemA
petA
psbJ
psbF
psbE
trnW-CCA
trnP-UGG
petL
petG
rps16
trnK-UUU
matK
psbA
trnH-GUG
rpl2
rps7
rps12
trnV-GAC
rrn16S
trnE-UUC
trnN-GUU
rrn23S
rrn4.5S
rrn5S
trnR-ACG
trnN-GUU
rpl32
trnL-UAG
ccsA
ndhD
psaC
ndhG
rps15
ycf1
trnL-CAA
ycf2
trnM-CAU
rpl2
rps19
rpl22
rps3
rpl16
rpl14
rps8
infA
rps11
rpoA
petD
petB
psbH
psbT
psbN
psbB
rpl20
rps18
rpl33
psaJ
trnQ-UUG
psbK
psbI
trnS-GCU
clpP
rps12
trnR-UCU
atpA
atpF
atpH
atpI
rps2
rpoC2
rpoC1
rpoB
trnC-GCA
petN
psbM
trnD-GUC
trnY-GUA
trnE-UUC
trnT-GGU
trnS-UGA
psbD
psbC
psbZ
trnG-GCC
trnM-CAU
rps14
accD
photosystem I
photosystem II
cytochrome b/f complex
ATP synthase
NADH dehydrogenase
RubisCO large subunit
RNA polymerase
ribosomal proteins (SSU)
ribosomal proteins (LSU)
transfer RNAs
ribosomal RNAs
clpP, matK
other genes
hypothetical chloroplast reading frames (ycf)

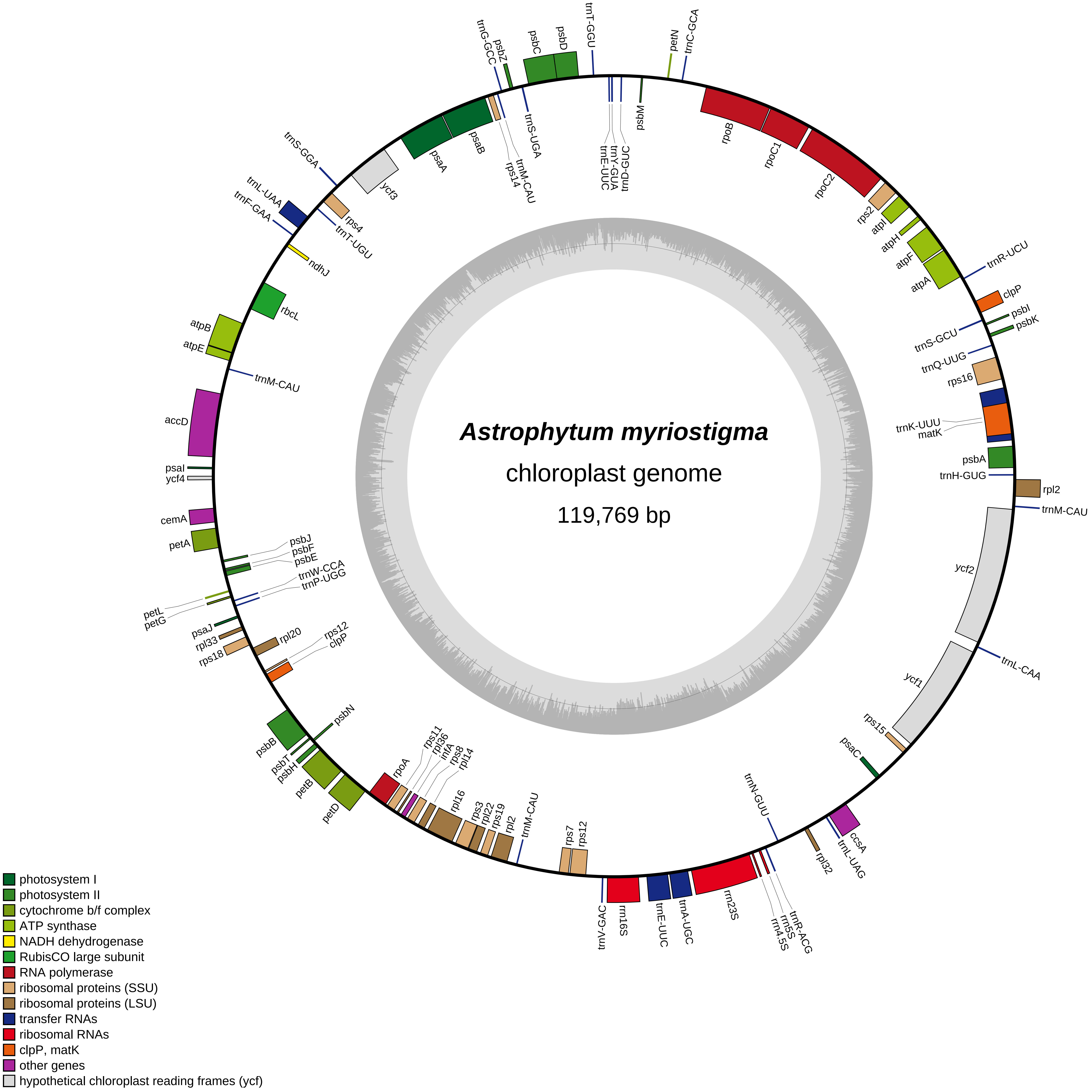
Astrophytum myriostigma
chloroplast genome
119,769 bp
trnG-GCC
psbZ
psbC
psbD
trnT-GGU
petN
trnC-GCA
trnS-UGA
trnE-UUC
trnY-GUA
trnD-GUC
psbM
rpoB
rpoC1
rpoC2
rps2
atpI
atpH
atpF
atpA
trnR-UCU
clpP
psbI
psbK
trnS-GCU
trnQ-UUG
rps16
trnK-UUU
matK
psbA
trnH-GUG
rpl2
trnM-CAU
ycf2
trnL-CAA
ycf1
rps15
psaC
ccsA
trnL-UAG
rpl32
trnN-GUU
trnR-ACG
rrn5S
rrn4.5S
rrn23S
trnA-UGC
trnE-UUC
rrn16S
trnV-GAC
rps12
rps7
trnM-CAU
rpl2
rps19
rpl22
rps3
rpl16
rpl14
rps8
infA
rpl36
rps11
rpoA
petD
petB
psbH
psbT
psbB
psbN
rps12
clpP
rpl20
rps18
rpl33
psaJ
petG
petL
trnP-UGG
trnW-CCA
psbE
psbF
psbJ
petA
cemA
ycf4
psaI
accD
trnM-CAU
atpE
atpB
rbcL
ndhJ
trnF-GAA
trnL-UAA
trnT-UGU
rps4
trnS-GGA
ycf3
psaA
psaB
rps14
trnM-CAU
photosystem I
photosystem II
cytochrome b/f complex
ATP synthase
NADH dehydrogenase
RubisCO large subunit
RNA polymerase
ribosomal proteins (SSU)
ribosomal proteins (LSU)
transfer RNAs
ribosomal RNAs
clpP, matK
other genes
hypothetical chloroplast reading frames (ycf)

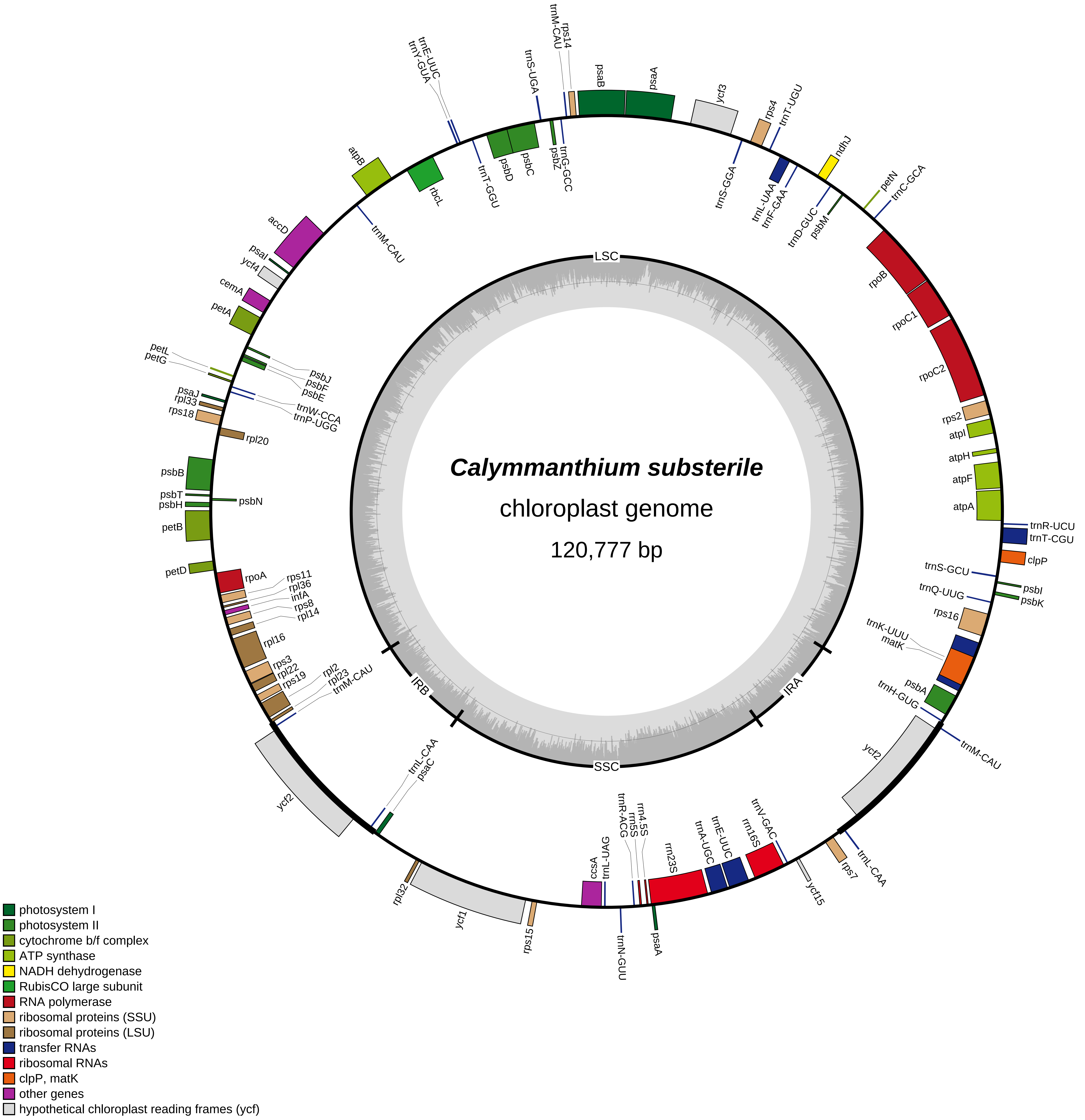
Calymmanthium substerile
chloroplast genome
120,777 bp
LSC
SSC
IRA
IRB
psaB
psaA
ycf3
rps4
trnT-UGU
ndhJ
petN
trnC-GCA
trnS-GGA
trnL-UAA
trnF-GAA
trnD-GUC
psbM
rpoB
rpoC1
rpoC2
rps2
atpI
atpH
atpF
atpA
trnR-UCU
trnT-CGU
clpP
trnS-GCU
psbI
trnQ-UUG
psbK
rps16
trnK-UUU
matK
psbA
trnH-GUG
trnM-CAU
ycf2
trnL-CAA
rps7
ycf15
trnV-GAC
rrn16S
trnE-UUC
trnA-UGC
rrn23S
rrn4.5S
rrn5S
trnR-ACG
psaA
trnN-GUU
trnL-UAG
ccsA
rps15
ycf1
rpl32
psaC
trnL-CAA
ycf2
trnM-CAU
rpl23
rpl2
rps19
rpl22
rps3
rpl16
rpl14
rps8
infA
rpl36
rps11
rpoA
petD
petB
psbH
psbT
psbN
psbB
rpl20
rps18
rpl33
psaJ
trnP-UGG
trnW-CCA
psbE
psbF
psbJ
petG
petL
petA
cemA
ycf4
psaI
accD
trnM-CAU
atpB
rbcL
trnT-GGU
psbD
psbC
psbZ
trnG-GCC
trnS-UGA
trnM-CAU
rps14
trnY-GUA
trnE-UUC
photosystem I
photosystem II
cytochrome b/f complex
ATP synthase
NADH dehydrogenase
RubisCO large subunit
RNA polymerase
ribosomal proteins (SSU)
ribosomal proteins (LSU)
transfer RNAs
ribosomal RNAs
clpP, matK
other genes
hypothetical chloroplast reading frames (ycf)

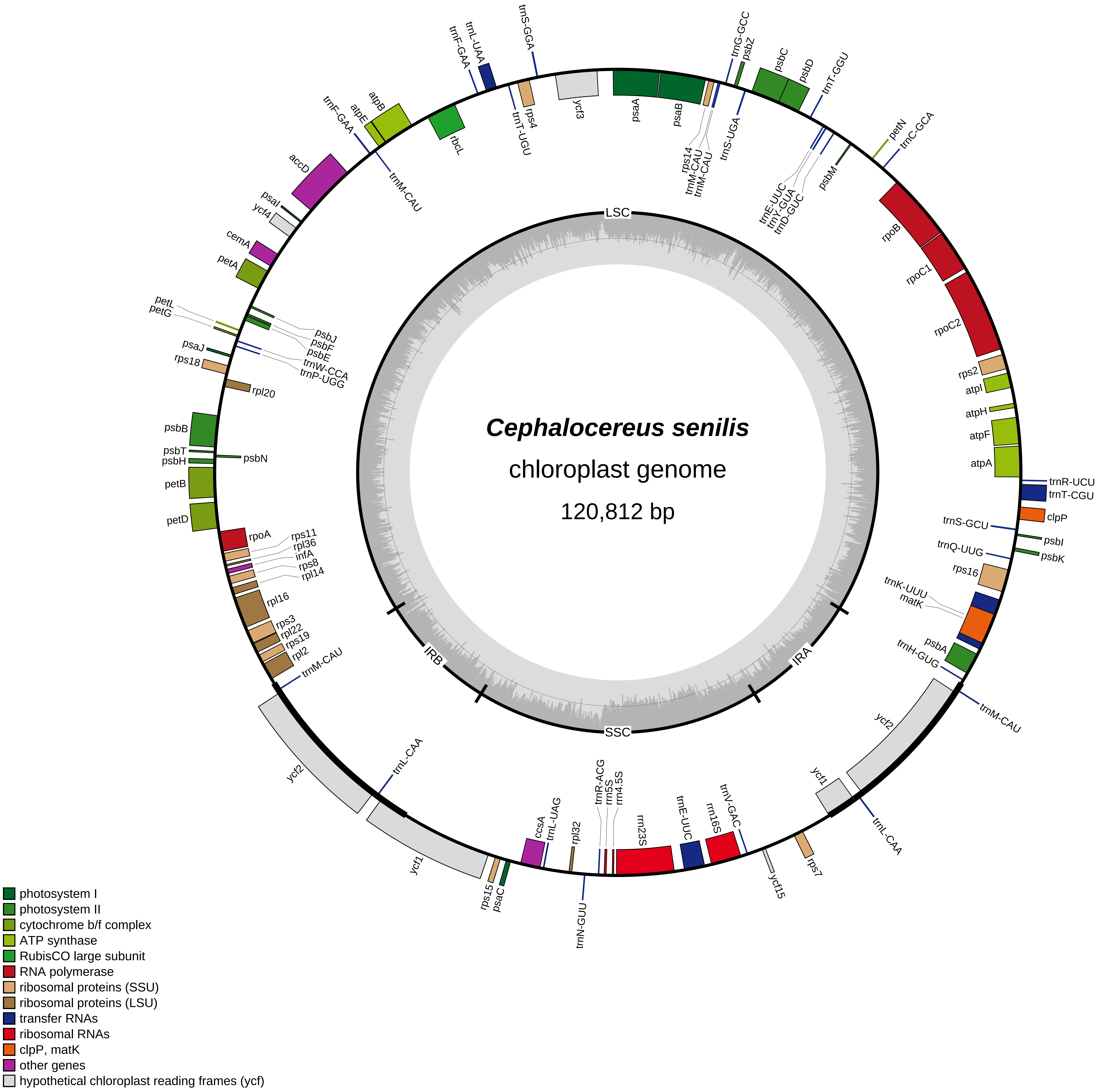

Cephalocereus senilis
chloroplast genome
120,812 bp
LSC
IRA
SSC
IRB
trnS-GGA
trnL-UAA
trnF-GAA
atpB
atpE
trnF-GAA
accD
psaI
ycf4
cemA
petA
petL
petG
psaJ
rps18
rpl20
psbB
psbT
psbH
psbN
petB
petD
rpoA
rps11
rpl36
infA
rps8
rpl14
rpl16
rps3
rpl22
rps19
rpl2
trnM-CAU
ycf2
trnL-CAA
ycf1
rps15
psaC
ccsA
trnL-UAG
rpl32
trnN-GUU
trnR-ACG
rrn5S
rrn4.5S
rrn23S
trnE-UUC
rrn16S
trnV-GAC
ycf15
rps7
trnL-CAA
ycf1
ycf2
trnM-CAU
trnH-GUG
psbA
trnK-UUU
matK
rps16
trnQ-UUG
psbK
psbI
trnS-GCU
clpP
trnT-CGU
trnR-UCU
atpA
atpF
atpH
atpI
rps2
rpoC2
rpoC1
rpoB
trnC-GCA
petN
psbM
trnD-GUC
trnY-GUA
trnE-UUC
trnT-GGU
psbD
psbC
psbZ
trnG-GCC
trnS-UGA
trnM-CAU
trnM-CAU
rps14
psaB
psaA
ycf3
rps4
trnT-UGU
rbcL
trnM-CAU
psbJ
psbF
psbE
trnW-CCA
trnP-UGG
photosystem I
photosystem II
cytochrome b/f complex
ATP synthase
RubisCO large subunit
RNA polymerase
ribosomal proteins (SSU)
ribosomal proteins (LSU)
transfer RNAs
ribosomal RNAs
clpP, matK
other genes
hypothetical chloroplast reading frames (ycf)

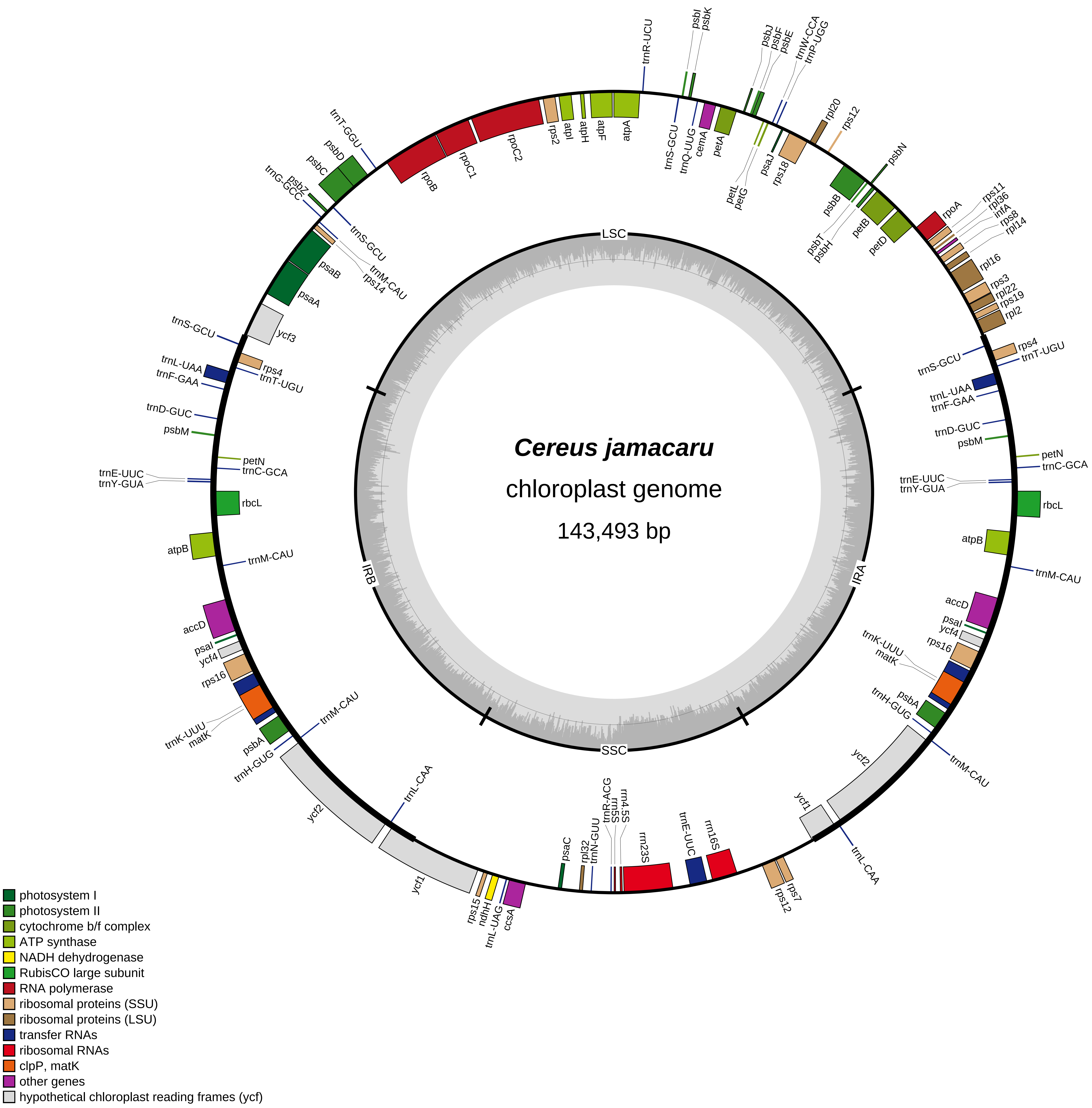

Cereus jamacaru
chloroplast genome
143,493 bp
LSC
IRA
SSC
IRB
trnR-UCU
psbI
psbK
psbJ
psbF
psbE
trnW-CCA
trnP-UGG
rpl20
rps12
trnS-GCU
trnQ-UUG
cemA
petA
petL
petG
psaJ
rps18
psbN
psbB
psbT
psbH
petB
petD
rpoA
rps11
rpl36
infA
rps8
rpl14
rpl16
rps3
rpl22
rps19
rpl2
rps4
trnT-UGU
trnL-UAA
trnF-GAA
trnD-GUC
psbM
petN
trnC-GCA
trnE-UUC
trnY-GUA
rbcL
atpB
trnM-CAU
accD
psaI
ycf4
rps16
trnK-UUU
matK
psbA
trnH-GUG
ycf2
ycf1
trnL-CAA
rps7
rrn16S
trnE-UUC
rrn23S
rrn4.5S
rrn5S
trnR-ACG
trnN-GUU
rpl32
psaC
ccsA
trnL-UAG
ndhH
rps15
ycf3
psaA
psaB
rps14
trnM-CAU
trnS-GCU
trnG-GCC
psbZ
psbC
psbD
trnT-GGU
rpoB
rpoC1
rpoC2
rps2
atpI
atpH
atpF
atpA
photosystem I
photosystem II
cytochrome b/f complex
ATP synthase
NADH dehydrogenase
RubisCO large subunit
RNA polymerase
ribosomal proteins (SSU)
ribosomal proteins (LSU)
transfer RNAs
ribosomal RNAs
clpP, matK
other genes
hypothetical chloroplast reading frames (ycf)

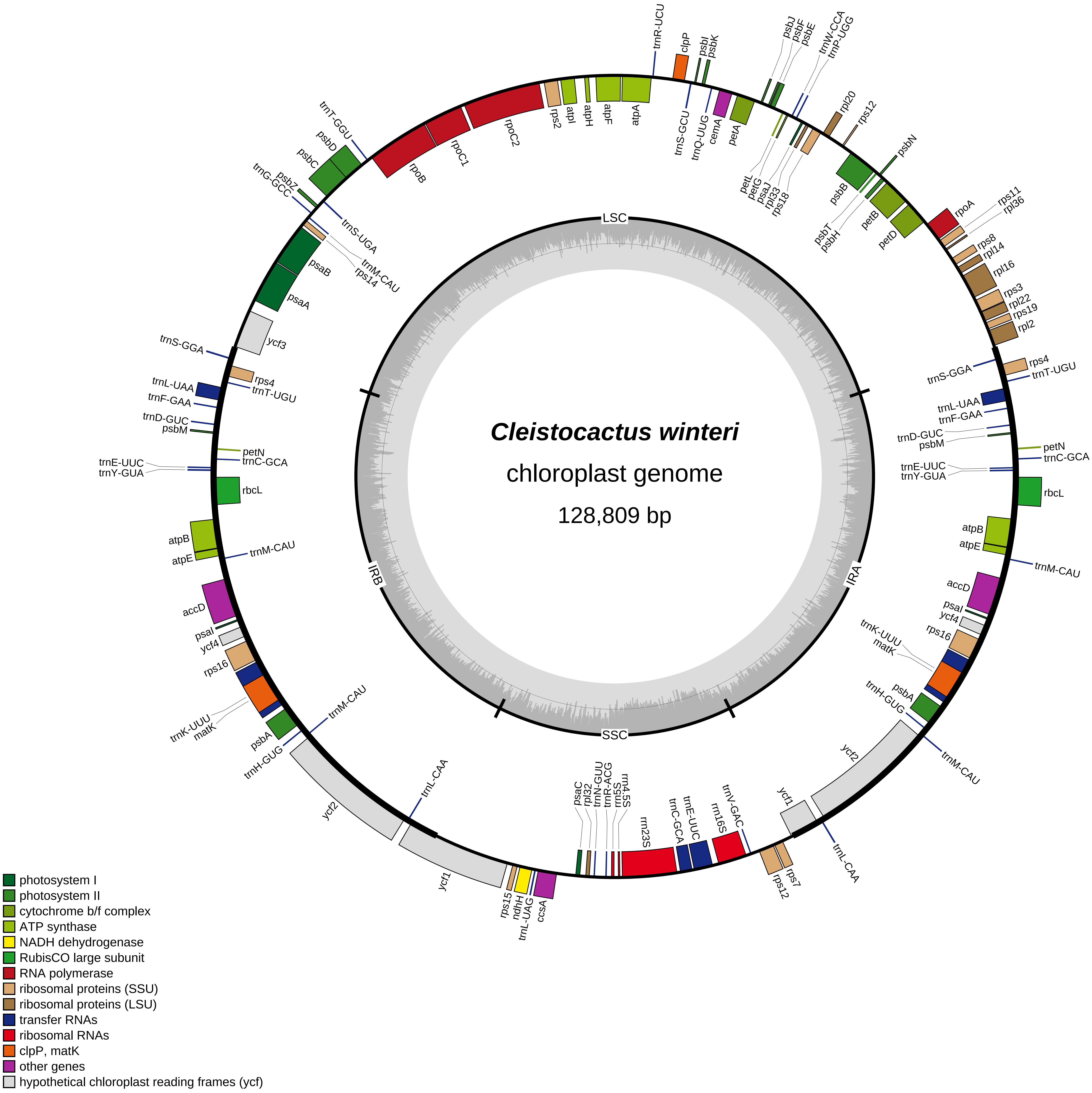

Cleistocactus winteri
chloroplast genome
128,809 bp
LSC
SSC
IRA
IRB
trnR-UCU
clpP
psbI
psbK
psbJ
psbF
psbE
trnW-CCA
trnP-UGG
rpl20
rps12
psbN
rpoA
rps11
rpl36
rps8
rpl14
rpl16
rps3
rpl22
rps19
rpl2
trnS-GCU
trnQ-UUG
cemA
petA
petL
petG
psaJ
rpl33
rps18
psbB
psbT
psbH
petB
petD
rpoC2
rpoC1
rpoB
rps2
atpI
atpH
atpF
atpA
trnT-GGU
psbD
psbC
psbZ
trnG-GCC
trnS-UGA
trnM-CAU
rps14
psaB
psaA
ycf3
trnS-GGA
rps4
trnT-UGU
trnL-UAA
trnF-GAA
trnD-GUC
psbM
petN
trnC-GCA
trnE-UUC
trnY-GUA
rbcL
atpB
atpE
accD
psaI
ycf4
rps16
trnK-UUU
matK
psbA
trnH-GUG
ycf2
trnL-CAA
ycf1
rps15
ndhH
trnL-UAG
ccsA
psaC
rpl32
trnN-GUU
trnR-ACG
rrn5S
rrn4.5S
rrn23S
trnA-UGC
trnI-GAU
rrn16S
trnV-GAC
rps12
rps7
photosystem I
photosystem II
cytochrome b/f complex
ATP synthase
NADH dehydrogenase
RubisCO large subunit
RNA polymerase
ribosomal proteins (SSU)
ribosomal proteins (LSU)
transfer RNAs
ribosomal RNAs
clpP, matK
other genes
hypothetical chloroplast reading frames (ycf)

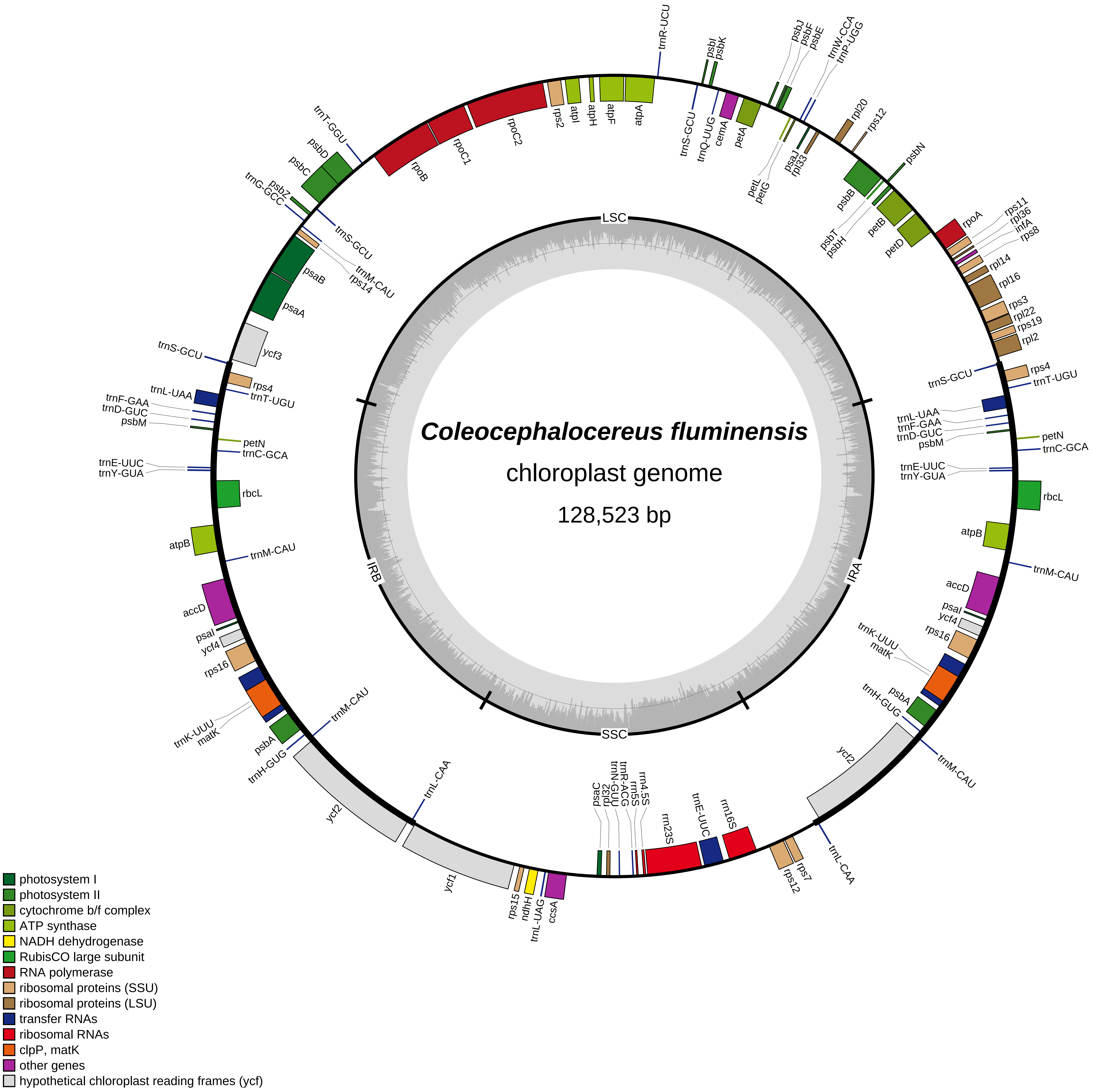
Coleocephalocereus fluminensis
chloroplast genome
128,523 bp
LSC
IRA
SSC
IRB
trnR-UCU
psbI
psbK
psbJ
psbF
psbE
trnW-CCA
trnP-UGG
rpl20
rps12
psbN
rpoA
rps11
rpl36
infA
rps8
rpl14
rpl16
rps3
rpl22
rps19
rpl2
rps4
trnS-GCU
trnT-UGU
trnL-UAA
trnF-GAA
trnD-GUC
psbM
petN
trnC-GCA
trnE-UUC
trnY-GUA
rbcL
atpB
trnM-CAU
accD
psaI
ycf4
rps16
trnK-UUU
matK
psbA
trnH-GUG
ycf2
trnL-CAA
rps7
rrn16S
rrn23S
rrn4.5S
rrn5S
trnR-ACG
trnN-GUU
rpl32
psaC
ccsA
trnL-UAG
ndhH
rps15
ycf1
ycf3
psaA
psaB
rps14
trnM-CAU
trnS-GCU
trnG-GCC
psbZ
psbC
psbD
trnT-GGU
rpoB
rpoC1
rpoC2
rps2
atpI
atpH
atpF
atpA
trnS-GCU
trnQ-UUG
cemA
petA
petL
petG
psaJ
rpl33
psbB
psbT
psbH
petB
petD
photosystem I
photosystem II
cytochrome b/f complex
ATP synthase
NADH dehydrogenase
RubisCO large subunit
RNA polymerase
ribosomal proteins (SSU)
ribosomal proteins (LSU)
transfer RNAs
ribosomal RNAs
clpP, matK
other genes
hypothetical chloroplast reading frames (ycf)

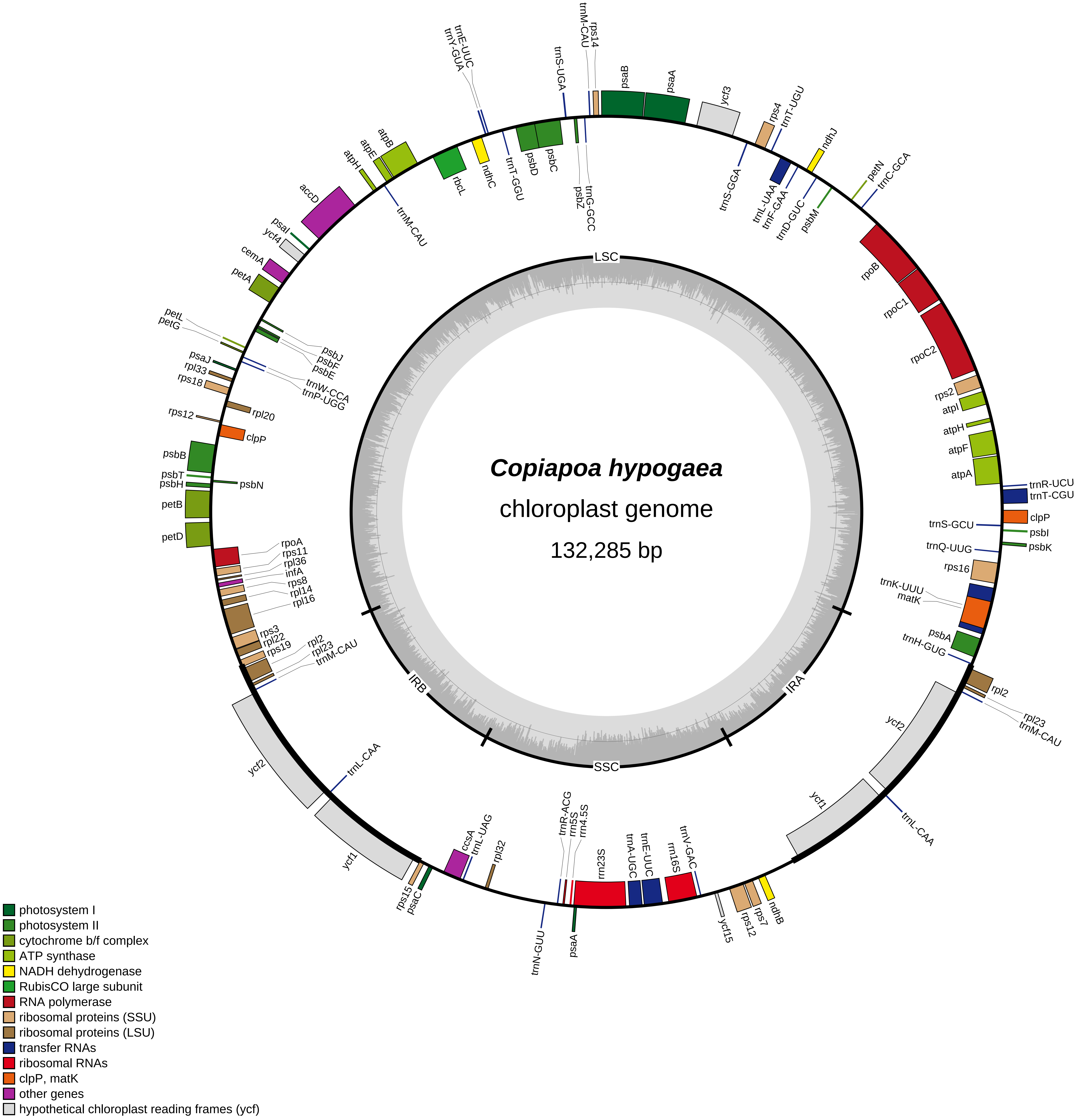
Copiapoa hypogaea
chloroplast genome
132,285 bp
LSC
SSC
IRA
IRB
psaB
psaA
ycf3
rps4
trnT-UGU
ndhJ
petN
trnC-GCA
trnS-GGA
trnL-UAA
trnF-GAA
trnD-GUC
psbM
rpoB
rpoC1
rpoC2
rps2
atpI
atpH
atpF
atpA
trnR-UCU
trnT-CGU
clpP
trnS-GCU
psbI
trnQ-UUG
psbK
rps16
trnK-UUU
matK
psbA
trnH-GUG
rpl2
rpl23
trnM-CAU
ycf2
ycf1
trnL-CAA
ndhB
rps7
rps12
ycf15
trnV-GAC
rrn16S
trnE-UUC
trnA-UGC
rrn23S
rrn4.5S
rrn5S
trnR-ACG
psaA
trnN-GUU
rpl32
trnL-UAG
ccsA
psaC
rps15
rps19
rpl22
rps3
rpl16
rpl14
rps8
infA
rpl36
rps11
rpoA
petD
petB
psbH
psbT
psbN
psbB
clpP
rps12
rpl20
rps18
rpl33
psaJ
trnP-UGG
trnW-CCA
psbE
psbF
psbJ
petG
petL
petA
cemA
ycf4
psaI
accD
atpH
atpE
atpB
trnM-CAU
rbcL
ndhC
trnT-GGU
psbD
psbC
psbZ
trnG-GCC
trnS-UGA
trnM-CAU
rps14
trnE-UUC
trnY-GUA
photosystem I
photosystem II
cytochrome b/f complex
ATP synthase
NADH dehydrogenase
RubisCO large subunit
RNA polymerase
ribosomal proteins (SSU)
ribosomal proteins (LSU)
transfer RNAs
ribosomal RNAs
clpP, matK
other genes
hypothetical chloroplast reading frames (ycf)

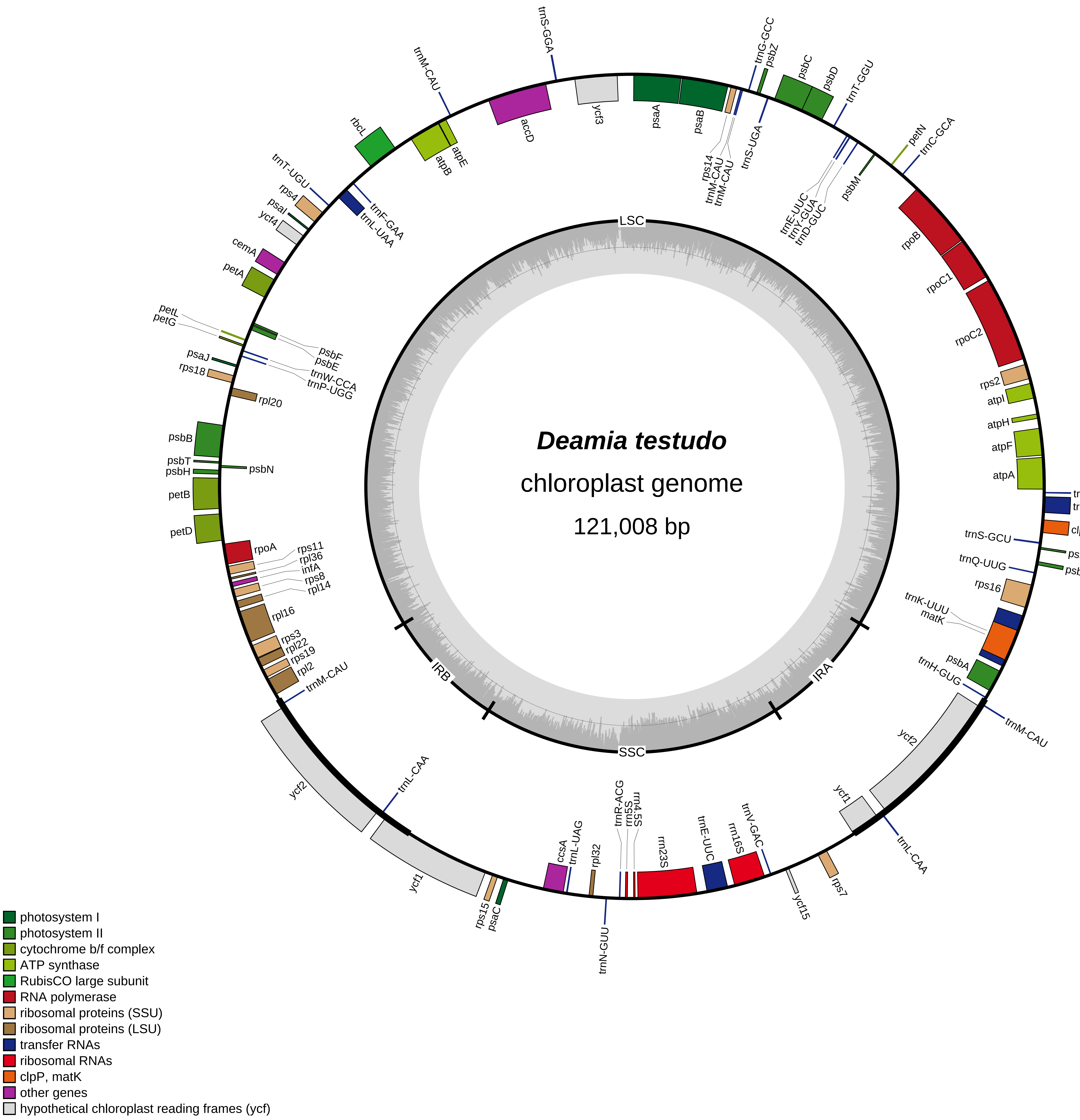
Deamia testudo
chloroplast genome
121,008 bp
LSC
IRA
SSC
IRB
trnS-GGA
trnM-CAU
rbcL
atpE
atpB
accD
ycf3
psaA
psaB
rps14
trnfM-CAU
trnM-CAU
trnG-GCC
psbZ
trnS-UGA
psbC
psbD
trnT-GGU
trnE-UUC
trnY-GUA
trnD-GUC
psbM
petN
trnC-GCA
rpoB
rpoC1
rpoC2
rps2
atpI
atpH
atpF
atpA
trnR-UCU
trnT-CGU
clpP
trnS-GCU
psbI
trnQ-UUG
psbK
rps16
trnK-UUU
matK
psbA
trnH-GUG
trnM-CAU
ycf2
ycf1
trnL-CAA
rps7
ycf15
trnV-GAC
rrn16S
trnE-UUC
rrn23S
rrn4.5S
rrn5S
trnR-ACG
trnN-GUU
rpl32
trnL-UAG
ccsA
psaC
rps15
ycf1
trnL-CAA
ycf2
trnM-CAU
rpl2
rps19
rpl22
rps3
rpl16
rpl14
rps8
infA
rpl36
rps11
rpoA
petD
petB
psbH
psbT
psbN
psbB
rpl20
rps18
psaJ
trnP-UGG
trnW-CCA
psbE
psbF
petG
petL
petA
cemA
ycf4
psaI
rps4
trnT-UGU
trnL-UAA
trnF-GAA
photosystem I
photosystem II
cytochrome b/f complex
ATP synthase
RubisCO large subunit
RNA polymerase
ribosomal proteins (SSU)
ribosomal proteins (LSU)
transfer RNAs
ribosomal RNAs
clpP, matK
other genes
hypothetical chloroplast reading frames (ycf)

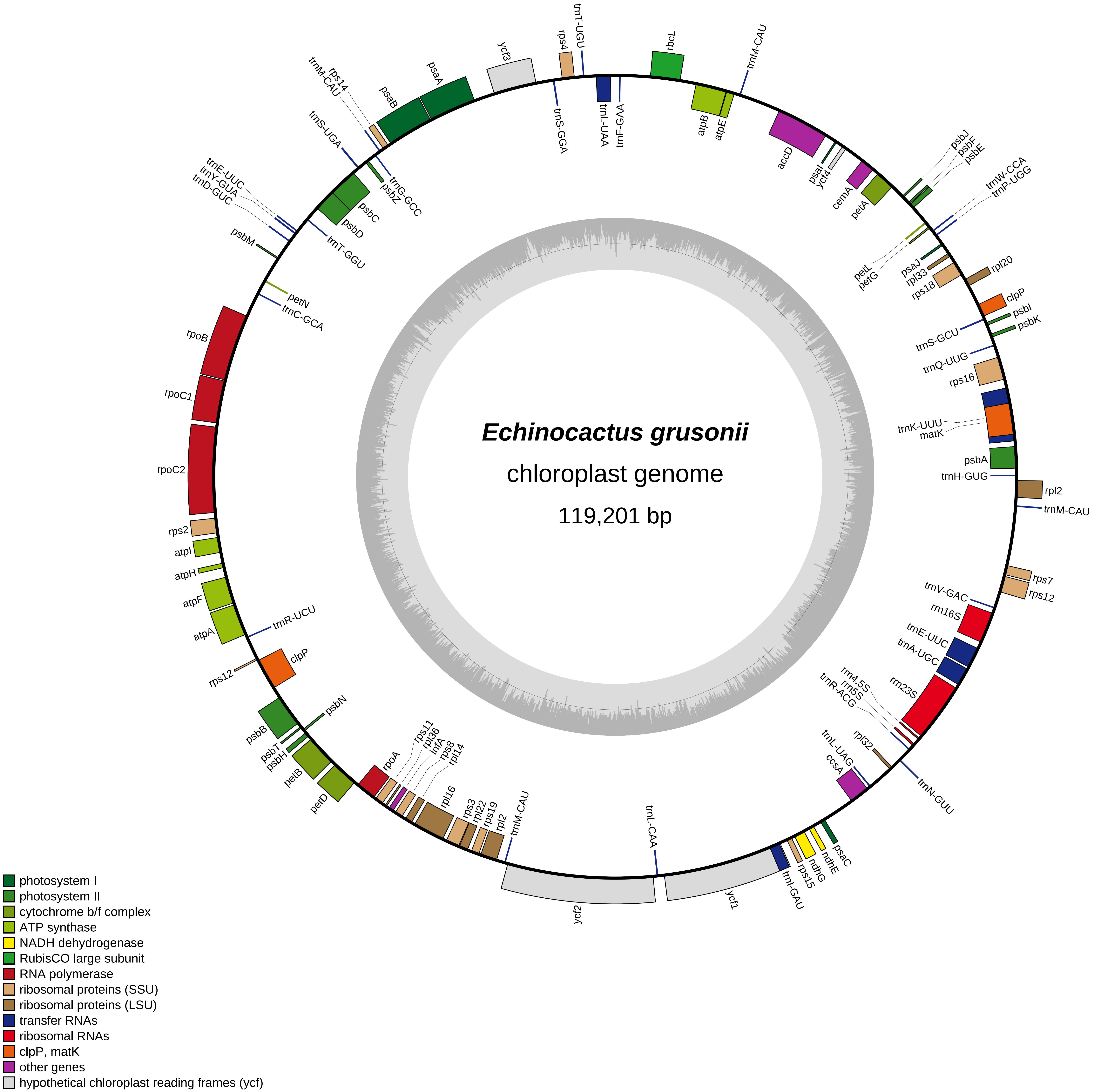

Echinocactus grusonii
chloroplast genome
119,201 bp
trnT-UGU
rps4
ycf3
psaA
psaB
rps14
trnM-CAU
trnS-UGA
trnS-GGA
trnL-UAA
trnF-GAA
rbcL
atpB
atpE
trnM-CAU
accD
psaI
ycf4
cemA
petA
psbJ
psbF
psbE
trnW-CCA
trnP-UGG
petL
petG
psaJ
rpl33
rps18
rpl20
clpP
psbI
psbK
trnS-GCU
trnQ-UUG
rps16
trnK-UUU
matK
psbA
trnH-GUG
rpl2
trnM-CAU
rps7
rps12
trnV-GAC
rrn16S
trnE-UUC
trnA-UGC
rrn23S
rrn4.5S
rrn5S
trnR-ACG
rpl32
trnN-GUU
trnL-UAG
ccsA
psaC
ndhE
ndhG
rps15
trnI-GAU
ycf1
trnL-CAA
ycf2
trnM-CAU
rpl2
rps19
rpl22
rps3
rpl16
rpl14
rps8
infA
rpl36
rps11
rpoA
petD
petB
psbH
psbT
psbN
psbB
rps12
clpP
trnR-UCU
atpA
atpF
atpH
atpI
rps2
rpoC2
rpoC1
rpoB
trnC-GCA
petN
psbM
trnD-GUC
trnY-GUA
trnE-UUC
trnT-GGU
psbD
psbC
psbZ
trnG-GCC
photosystem I
photosystem II
cytochrome b/f complex
ATP synthase
NADH dehydrogenase
RubisCO large subunit
RNA polymerase
ribosomal proteins (SSU)
ribosomal proteins (LSU)
transfer RNAs
ribosomal RNAs
clpP, matK
other genes
hypothetical chloroplast reading frames (ycf)

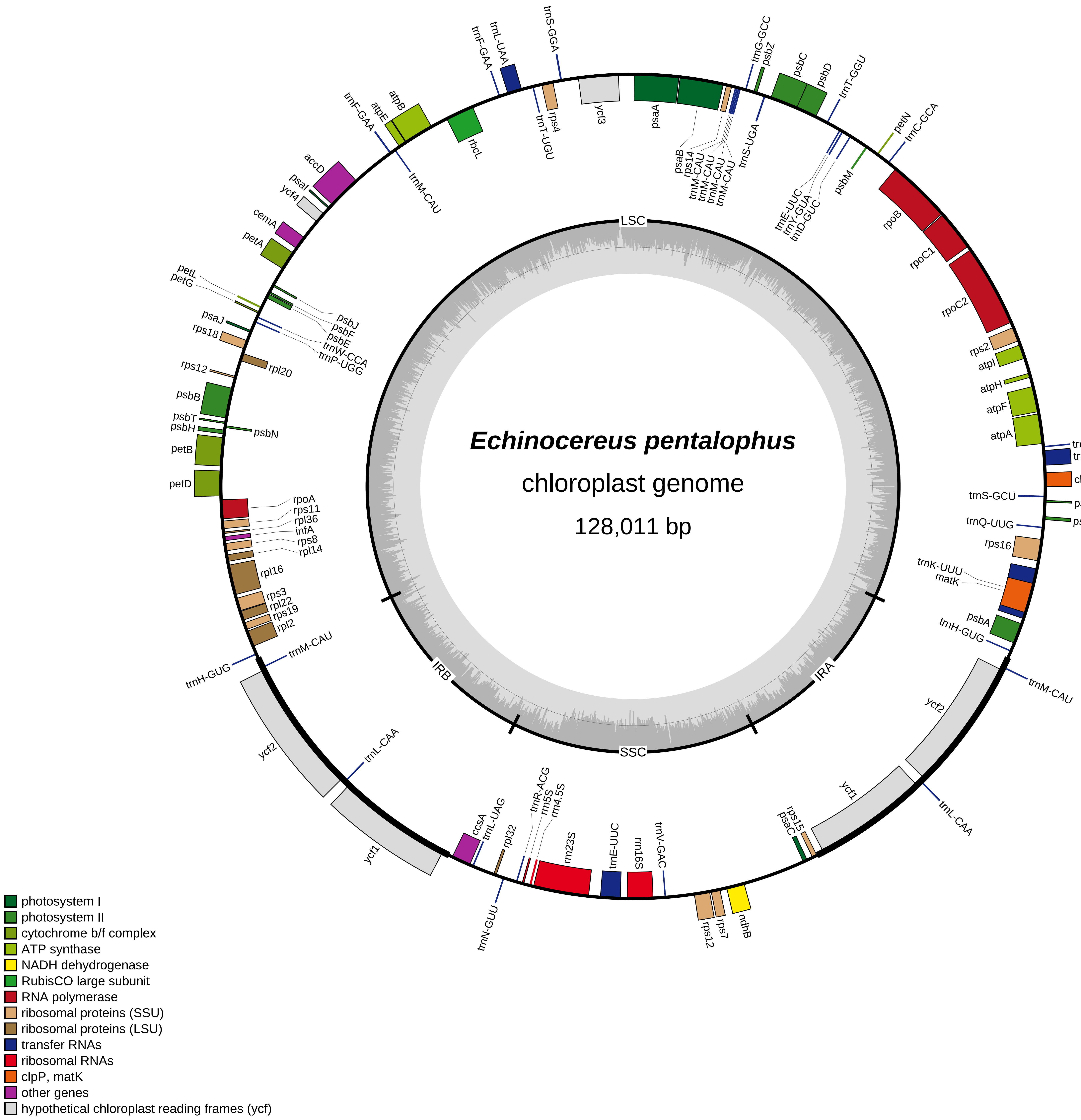
Echinocereus pentalophus
chloroplast genome
128,011 bp
LSC
IRA
IRB
SSC
trnS-GGA
trnL-UAA
trnF-GAA
atpB
atpE
trnF-GAA
trnM-CAU
rbcL
rps4
trnT-UGU
ycf3
psaA
psaB
rps14
trnM-CAU
trnM-CAU
trnM-CAU
trnM-CAU
trnS-UGA
trnG-GCC
psbZ
psbC
psbD
trnT-GGU
trnE-UUC
trnY-GUA
trnD-GUC
psbM
petN
trnC-GCA
rpoB
rpoC1
rpoC2
rps2
atpI
atpH
atpF
atpA
trnR-UCU
trnT-CGU
clpP
trnS-GCU
psbI
trnQ-UUG
psbK
rps16
trnK-UUU
matK
psbA
trnH-GUG
trnM-CAU
ycf2
trnL-CAA
ycf1
rps15
psaC
ndhB
rps7
rps12
trnV-GAC
rrn16S
trnE-UUC
rrn23S
rrn4.5S
rrn5S
trnR-ACG
rpl32
trnL-UAG
ccsA
trnN-GUU
ycf1
trnL-CAA
ycf2
trnH-GUG
trnM-CAU
rpl2
rps19
rpl22
rps3
rpl16
rpl14
rps8
infA
rpl36
rps11
rpoA
petD
petB
psbH
psbT
psbN
psbB
rps12
rpl20
rps18
psaJ
trnP-UGG
trnW-CCA
psbE
psbF
psbJ
petG
petL
petA
cemA
ycf4
psaI
accD
photosystem I
photosystem II
cytochrome b/f complex
ATP synthase
NADH dehydrogenase
RubisCO large subunit
RNA polymerase
ribosomal proteins (SSU)
ribosomal proteins (LSU)
transfer RNAs
ribosomal RNAs
clpP, matK
other genes
hypothetical chloroplast reading frames (ycf)

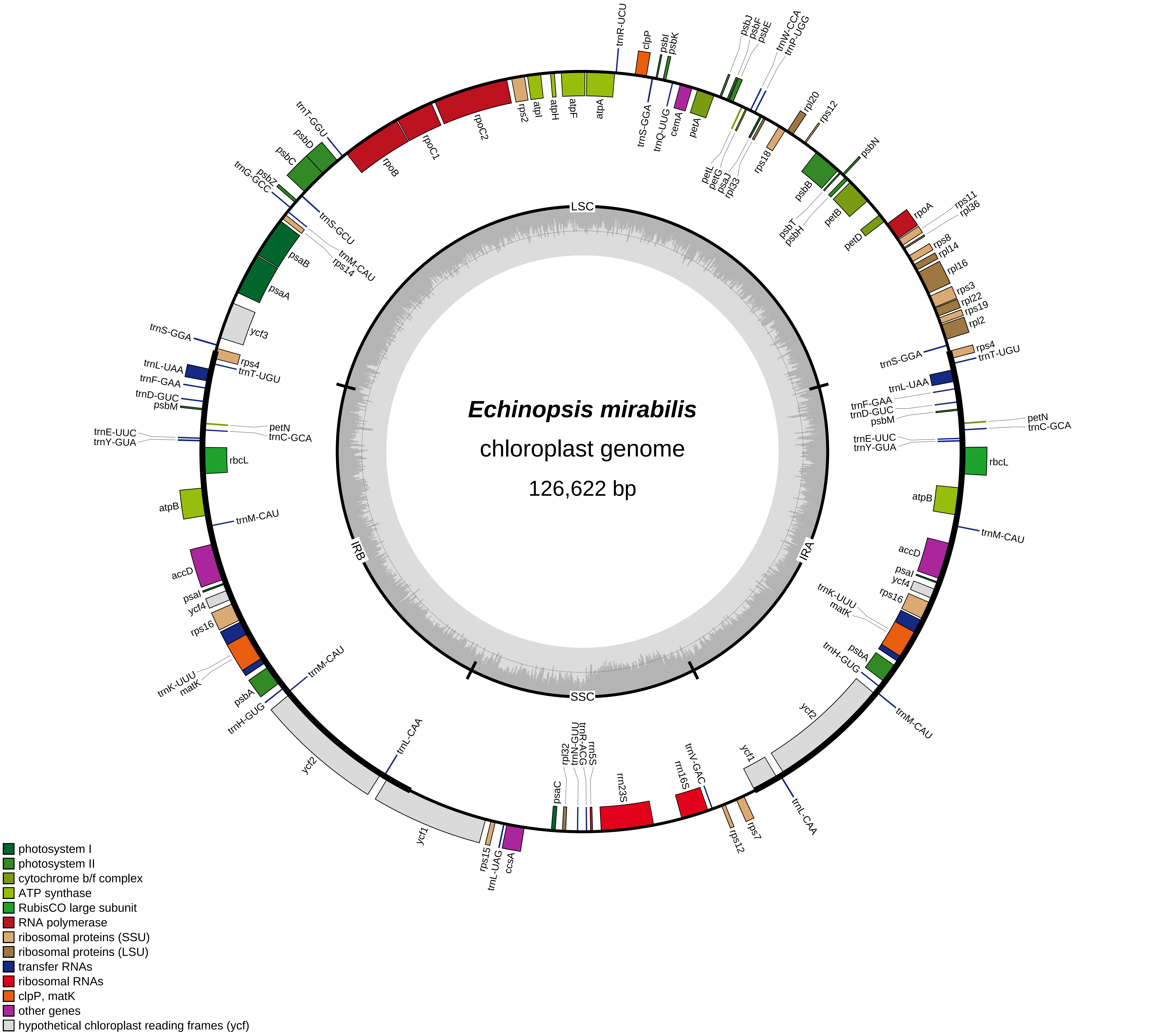
Echinopsis mirabilis
chloroplast genome
126,622 bp
LSC
IRA
SSC
IRB
trnR-UCU
clpP
psbI
psbK
psbJ
psbF
psbE
trnW-CCA
trnP-UGG
rpl20
rps12
trnS-GGA
trnQ-UUG
cemA
petA
petL
petG
psaJ
rpl33
rps18
psbN
psbB
psbT
psbH
petB
petD
rpoA
rps11
rpl36
rps8
rpl14
rpl16
rps3
rpl22
rps19
rpl2
rps4
trnT-UGU
trnL-UAA
trnF-GAA
trnD-GUC
psbM
petN
trnC-GCA
trnE-UUC
trnY-GUA
rbcL
atpB
trnM-CAU
accD
psaI
ycf4
rps16
trnK-UUU
matK
psbA
trnH-GUG
ycf2
ycf1
trnL-CAA
rps7
trnV-GAC
rrn16S
rrn23S
rrn5S
trnR-ACG
trnN-GUU
rpl32
psaC
ccsA
trnL-UAG
rps15
atpA
atpF
atpH
atpI
rps2
rpoC2
rpoC1
rpoB
trnT-GGU
psbD
psbC
psbZ
trnG-GCC
trnS-GCU
trnM-CAU
rps14
psaB
psaA
ycf3
photosystem I
photosystem II
cytochrome b/f complex
ATP synthase
RubisCO large subunit
RNA polymerase
ribosomal proteins (SSU)
ribosomal proteins (LSU)
transfer RNAs
ribosomal RNAs
clpP, matK
other genes
hypothetical chloroplast reading frames (ycf)

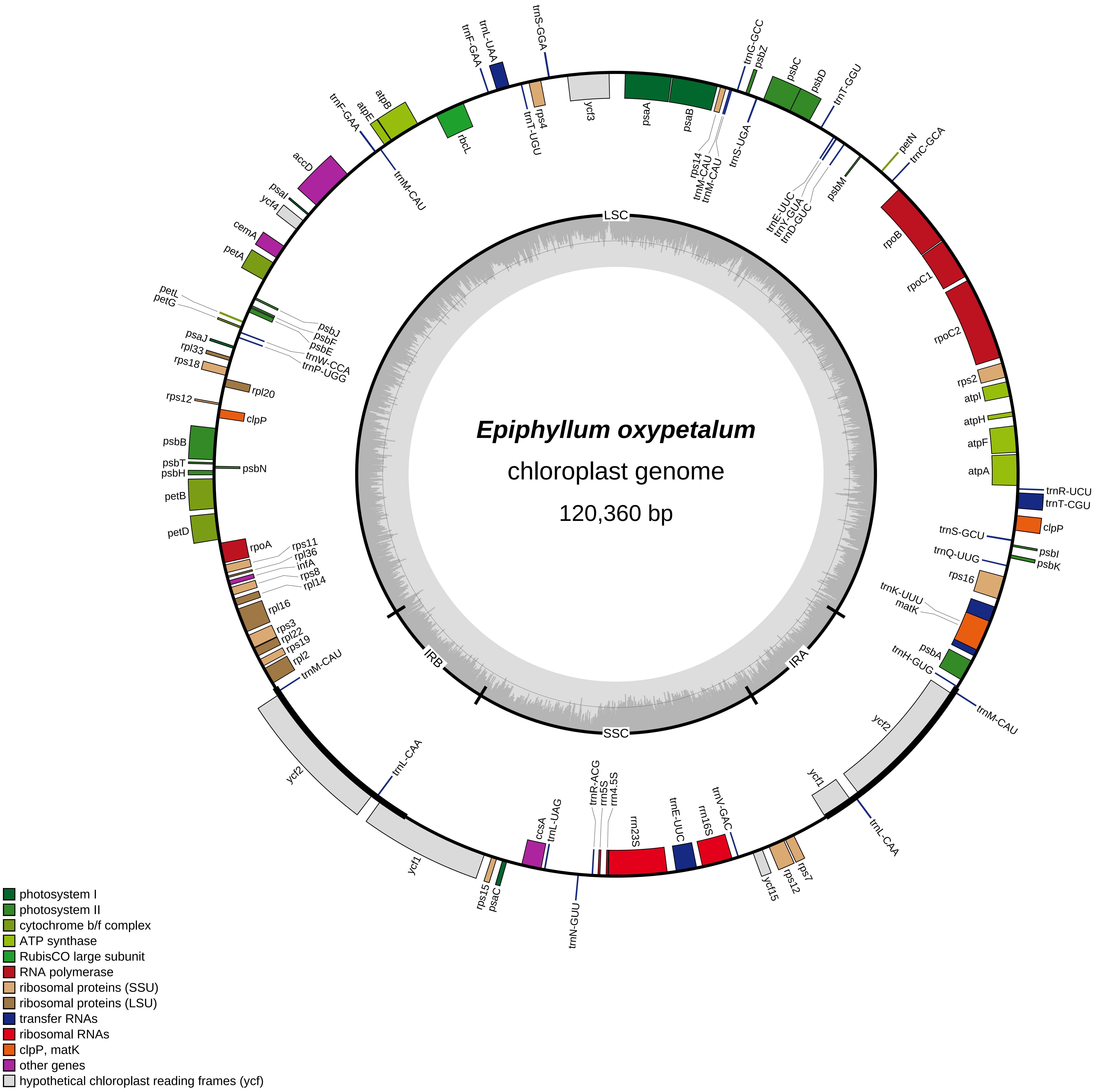

Epiphyllum oxypetalum
chloroplast genome
120,360 bp
LSC
IRA
SSC
IRB
trnS-GGA
trnL-UAA
trnF-GAA
atpB
atpE
trnF-GAA
trnM-CAU
rbcL
trnT-UGU
rps4
ycf3
psaA
psaB
rps14
trnM-CAU
trnM-CAU
trnG-GCC
psbZ
trnS-UGA
psbC
psbD
trnT-GGU
trnE-UUC
trnY-GUA
trnD-GUC
psbM
petN
trnC-GCA
rpoB
rpoC1
rpoC2
rps2
atpI
atpH
atpF
atpA
trnR-UCU
trnT-CGU
clpP
trnS-GCU
psbI
trnQ-UUG
psbK
rps16
trnK-UUU
matK
psbA
trnH-GUG
trnM-CAU
ycf2
ycf1
trnL-CAA
rps7
rps12
ycf15
trnV-GAC
rrn16S
trnE-UUC
rrn23S
rrn4.5S
rrn5S
trnR-ACG
trnN-GUU
trnL-UAG
ccsA
psaC
rps15
ycf1
trnL-CAA
ycf2
trnM-CAU
rpl2
rps19
rpl22
rps3
rpl16
rpl14
rps8
infA
rpl36
rps11
rpoA
petD
petB
psbH
psbT
psbN
psbB
clpP
rps12
rpl20
rps18
rpl33
psaJ
trnP-UGG
trnW-CCA
psbE
psbF
psbJ
petG
petL
petA
cemA
ycf4
psaI
accD
photosystem I
photosystem II
cytochrome b/f complex
ATP synthase
RubisCO large subunit
RNA polymerase
ribosomal proteins (SSU)
ribosomal proteins (LSU)
transfer RNAs
ribosomal RNAs
clpP, matK
other genes
hypothetical chloroplast reading frames (ycf)

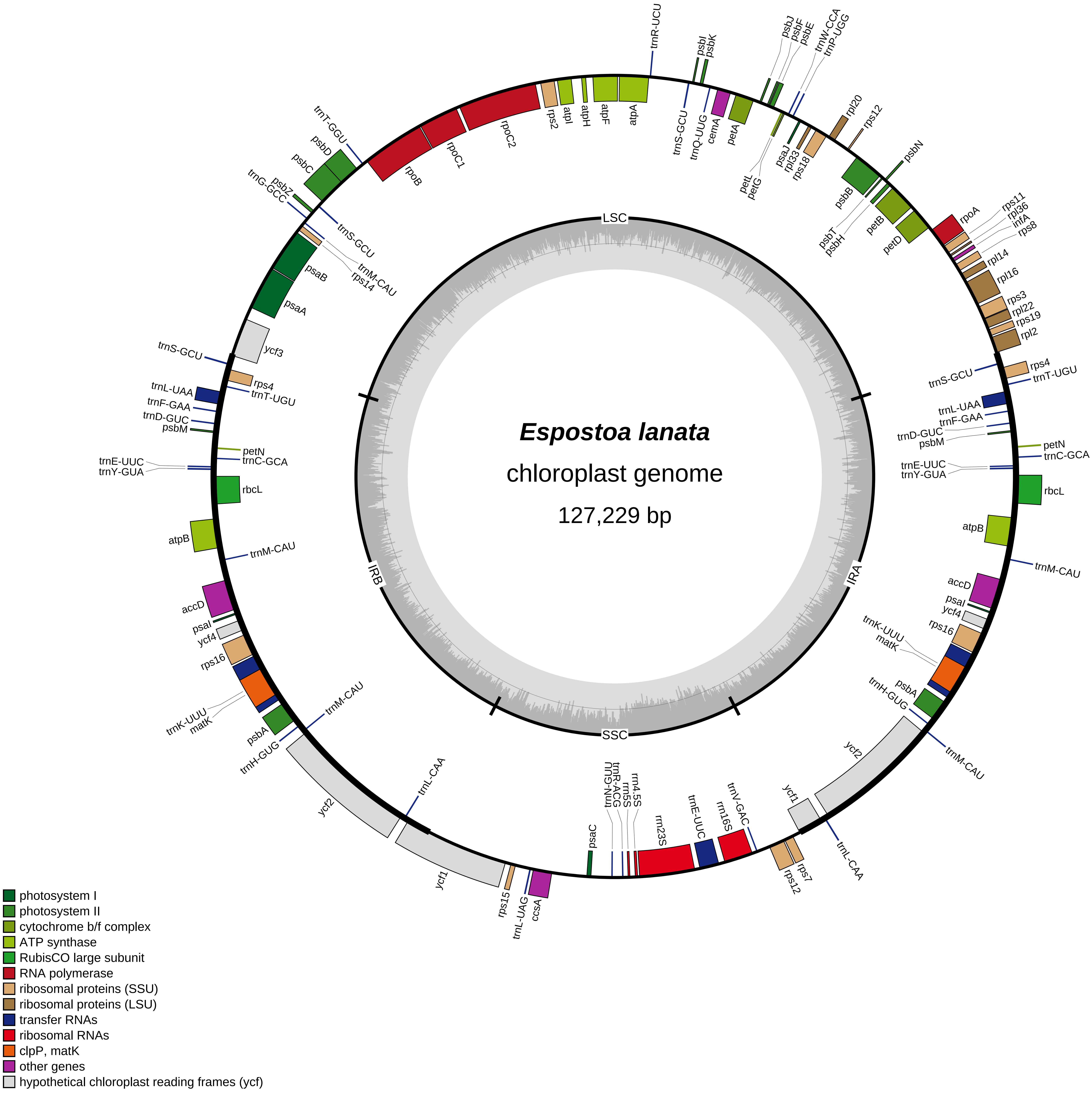

Espostoa lanata
chloroplast genome
127,229 bp
LSC
IRA
SSC
IRB
trnR-UCU
psbI
psbK
trnS-GCU
trnQ-UUG
cemA
petA
psbJ
psbF
psbE
trnW-CCA
trnP-UGG
petL
petG
psaJ
rpl33
rps18
rpl20
rps12
psbN
psbB
psbT
psbH
petB
petD
rpoA
rps11
rpl36
infA
rps8
rpl14
rpl16
rps3
rpl22
rps19
rpl2
rps4
trnT-UGU
trnL-UAA
trnF-GAA
trnD-GUC
psbM
petN
trnC-GCA
trnE-UUC
trnY-GUA
rbcL
atpB
trnM-CAU
accD
psaI
ycf4
rps16
trnK-UUU
matK
psbA
trnH-GUG
ycf2
ycf1
trnL-CAA
rps7
trnV-GAC
rrn16S
rrn23S
rrn4.5S
rrn5S
trnR-ACG
trnN-GUU
psaC
ccsA
trnL-UAG
rps15
trnG-GCC
psbZ
psbC
psbD
trnT-GGU
rpoB
rpoC1
rpoC2
rps2
atpI
atpH
atpF
atpA
trnS-GCU
trnM-CAU
rps14
psaB
psaA
ycf3
photosystem I
photosystem II
cytochrome b/f complex
ATP synthase
RubisCO large subunit
RNA polymerase
ribosomal proteins (SSU)
ribosomal proteins (LSU)
transfer RNAs
ribosomal RNAs
clpP, matK
other genes
hypothetical chloroplast reading frames (ycf)

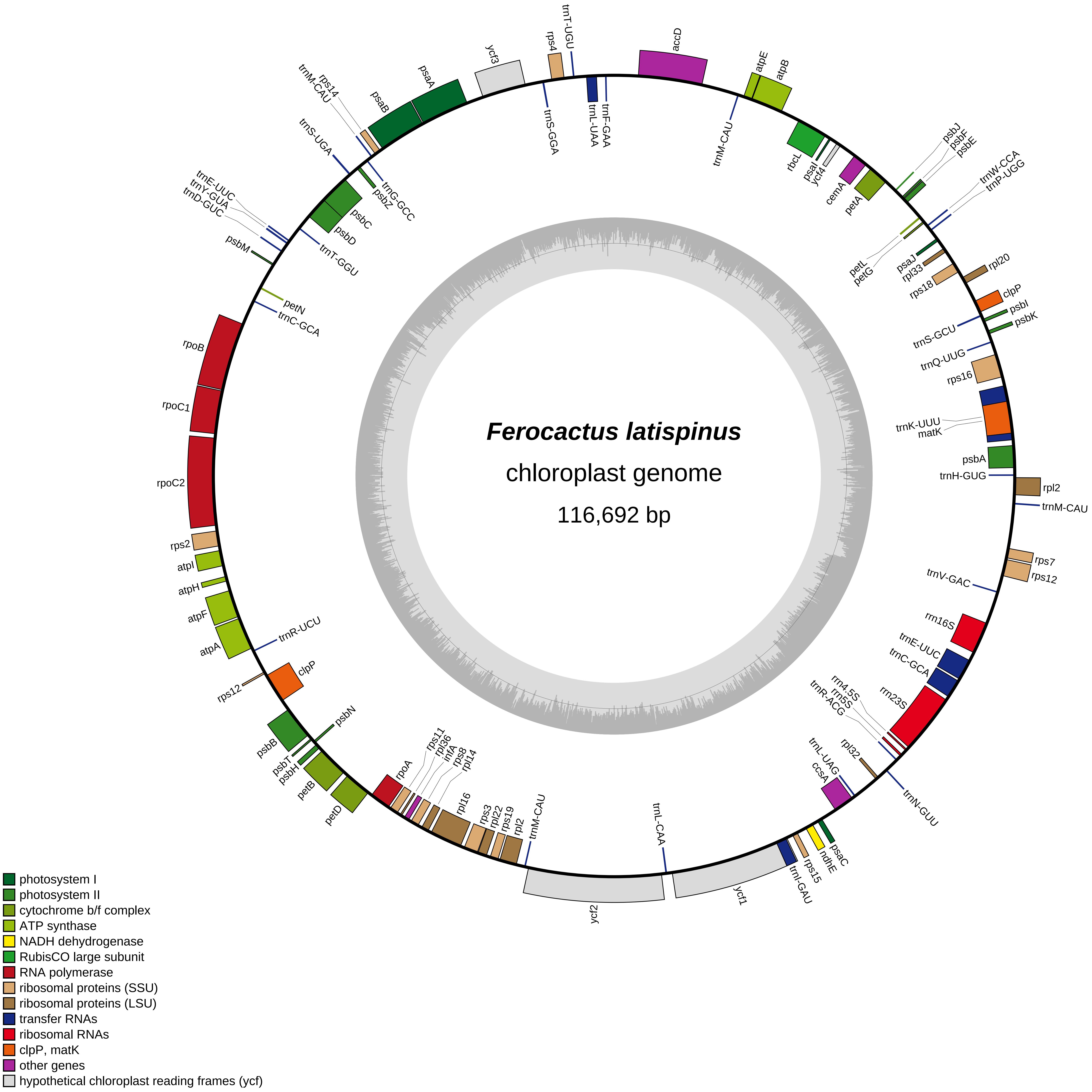
Ferocactus latispinus
chloroplast genome
116,692 bp
accD
atpE
atpB
trnT-UGU
rps4
ycf3
psaA
psaB
rps14
trnM-CAU
trnS-UGA
trnS-GGA
trnL-UAA
trnF-GAA
trnM-CAU
rbcL
psaI
ycf4
cemA
petA
psbJ
psbF
psbE
trnW-CCA
trnP-UGG
petL
petG
psaJ
rpl33
rps18
rpl20
clpP
psbI
psbK
trnS-GCU
trnQ-UUG
rps16
trnK-UUU
matK
psbA
trnH-GUG
rpl2
trnM-CAU
rps7
rps12
trnV-GAC
rrn16S
trnE-UUC
trnC-GCA
rrn23S
rrn4.5S
rrn5S
trnR-ACG
rpl32
trnL-UAG
ccsA
trnN-GUU
psaC
ndhE
rps15
trnI-GAU
ycf1
trnL-CAA
ycf2
trnM-CAU
rpl2
rps19
rpl22
rps3
rpl16
rpl14
rps8
infA
rpl36
rps11
rpoA
petD
petB
psbH
psbT
psbB
psbN
rps12
clpP
trnR-UCU
atpA
atpF
atpH
atpI
rps2
rpoC2
rpoC1
rpoB
trnC-GCA
petN
psbM
trnE-UUC
trnY-GUA
trnD-GUC
trnT-GGU
psbD
psbC
psbZ
trnG-GCC
photosystem I
photosystem II
cytochrome b/f complex
ATP synthase
NADH dehydrogenase
RubisCO large subunit
RNA polymerase
ribosomal proteins (SSU)
ribosomal proteins (LSU)
transfer RNAs
ribosomal RNAs
clpP, matK
other genes
hypothetical chloroplast reading frames (ycf)

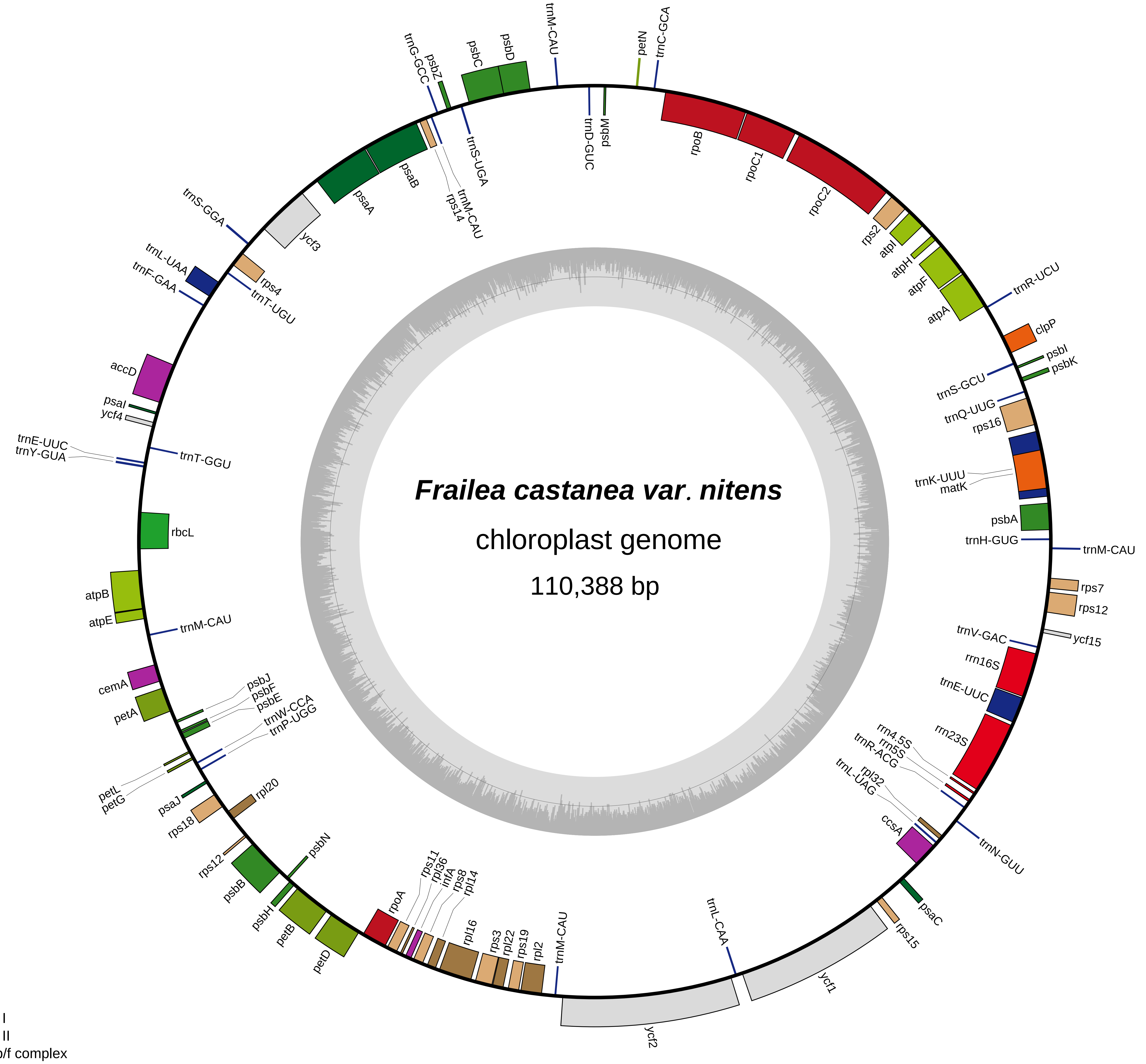

photosystem I
photosystem II
cytochrome b/f complex
ATP synthase
RubisCO large subunit
RNA polymerase
ribosomal proteins (SSU)
ribosomal proteins (LSU)
transfer RNAs
ribosomal RNAs
clpP, matK
other genes
hypothetical chloroplast reading frames (ycf)

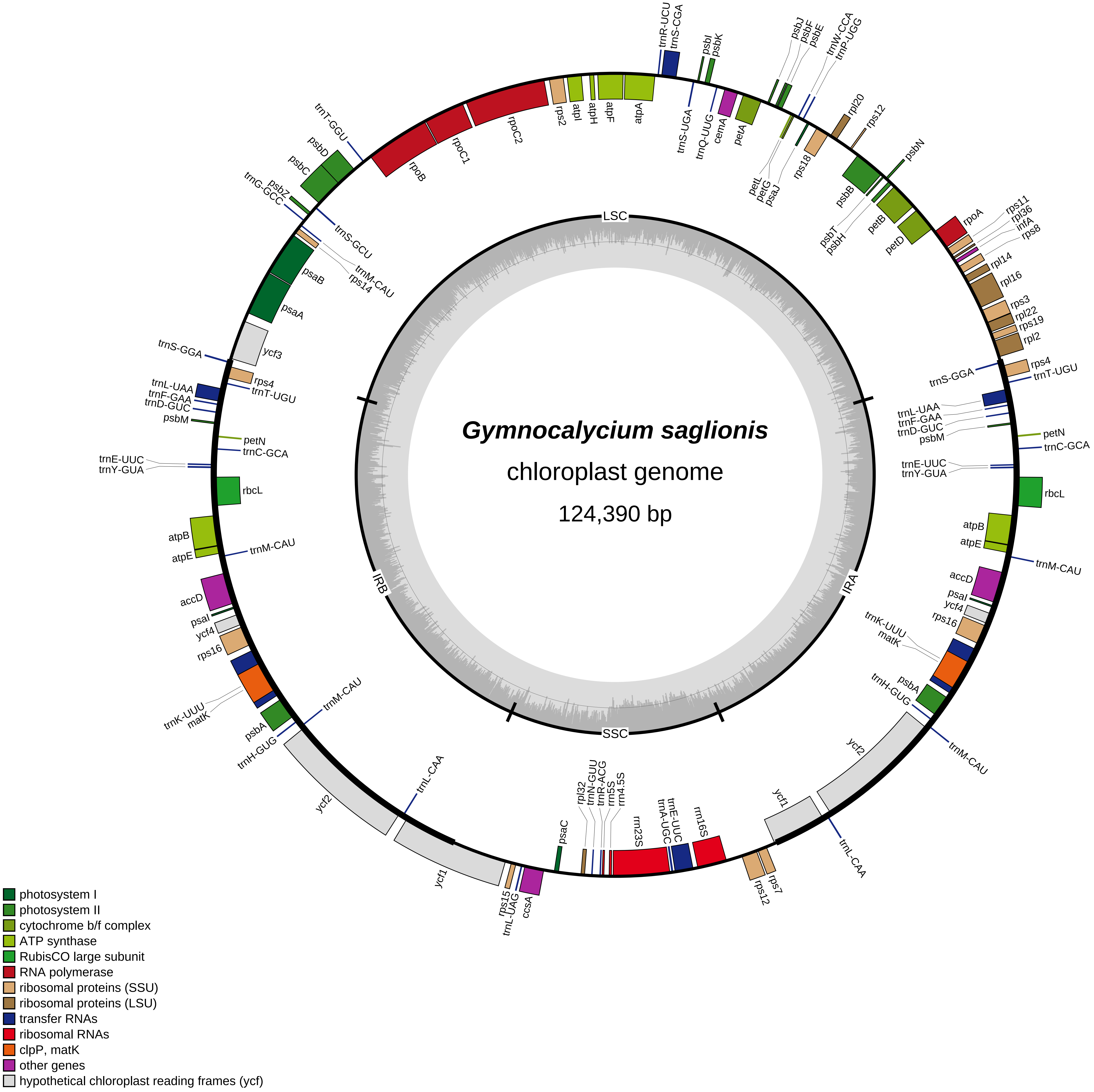

Gymnocalycium saglionis
chloroplast genome
124,390 bp
LSC
IRA
SSC
IRB
trnR-UCU
trnS-CGA
psbI
psbK
psbJ
psbF
psbE
trnW-CCA
trnP-UGG
rpl20
rps12
psbN
rpoA
rps11
rpl36
infA
rps8
rpl14
rpl16
rps3
rpl22
rps19
rpl2
rps4
trnT-UGU
trnS-GGA
trnL-UAA
trnF-GAA
trnD-GUC
psbM
petN
trnC-GCA
trnE-UUC
trnY-GUA
rbcL
atpB
atpE
trnM-CAU
accD
psaI
ycf4
rps16
trnK-UUU
matK
psbA
trnH-GUG
ycf2
ycf1
trnL-CAA
rps7
rrn16S
trnE-UUC
trnA-UGC
rrn23S
rrn4.5S
rrn5S
trnR-ACG
trnN-GUU
rpl32
psaC
ccsA
trnL-UAG
rps15
ycf3
psaA
psaB
rps14
trnM-CAU
trnS-GCU
trnG-GCC
psbZ
psbC
psbD
trnT-GGU
rpoB
rpoC1
rpoC2
rps2
atpI
atpH
atpF
atpA
trnS-UGA
trnQ-UUG
cemA
petA
petL
petG
psaJ
rps18
psbB
psbT
psbH
petB
petD
photosystem I
photosystem II
cytochrome b/f complex
ATP synthase
RubisCO large subunit
RNA polymerase
ribosomal proteins (SSU)
ribosomal proteins (LSU)
transfer RNAs
ribosomal RNAs
clpP, matK
other genes
hypothetical chloroplast reading frames (ycf)

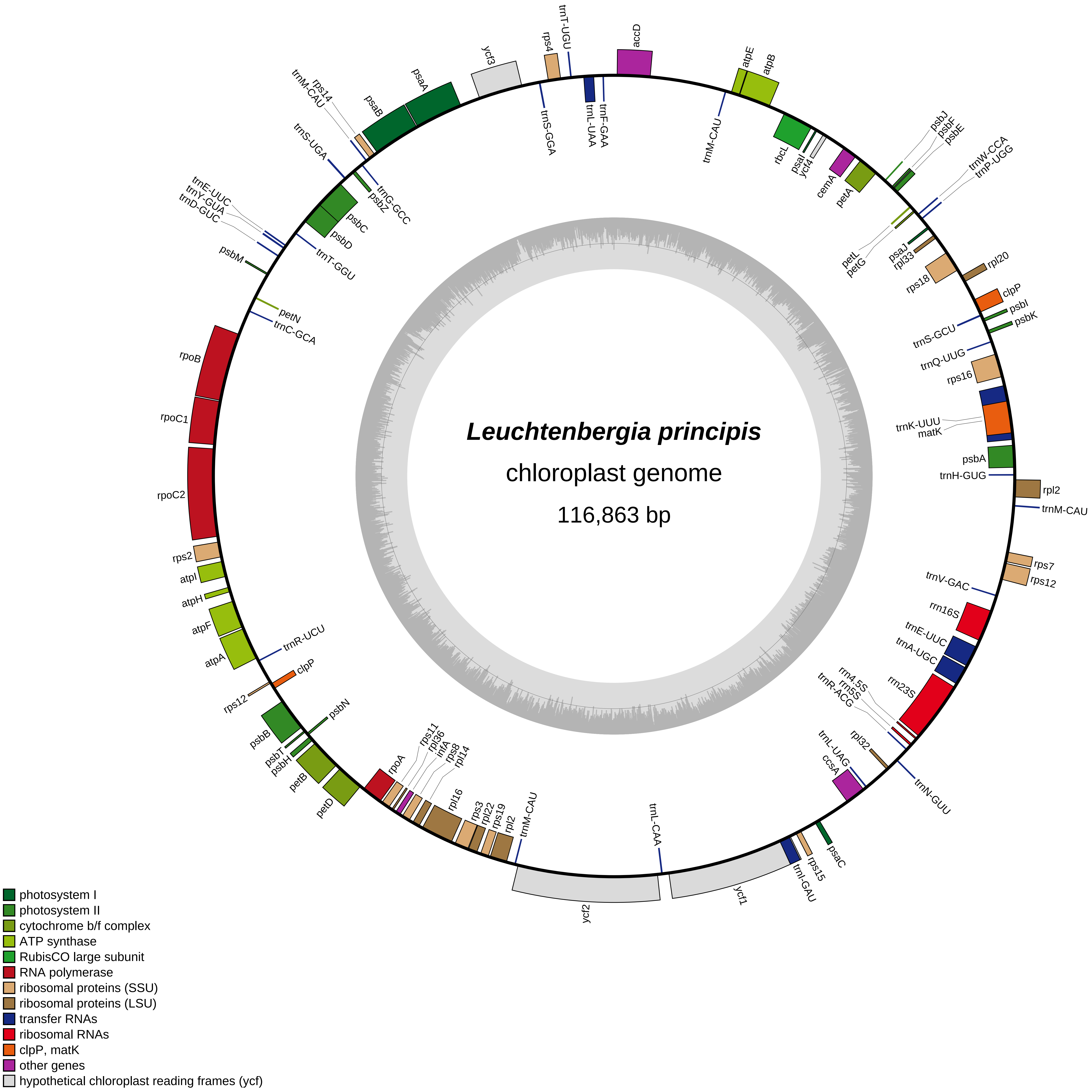
Leuchtenbergia principis
chloroplast genome
116,863 bp
accD
trnT-UGU
rps4
ycf3
psaA
psaB
rps14
trnM-CAU
trnS-UGA
trnS-GGA
trnL-UAA
trnF-GAA
trnM-CAU
atpE
atpB
rbcL
psaI
ycf4
cemA
petA
psbJ
psbF
psbE
trnW-CCA
trnP-UGG
petL
petG
psaJ
rpl33
rps18
rpl20
clpP
psbI
psbK
trnS-GCU
trnQ-UUG
rps16
trnK-UUU
matK
psbA
trnH-GUG
rpl2
trnM-CAU
rps7
rps12
trnV-GAC
rrn16S
trnE-UUC
trnA-UGC
rrn23S
rrn4.5S
rrn5S
trnR-ACG
rpl32
trnN-GUU
trnL-UAG
ccsA
psaC
rps15
trnI-GAU
ycf1
trnL-CAA
ycf2
trnM-CAU
rpl2
rps19
rpl22
rps3
rpl16
rpl14
rps8
infA
rpl36
rps11
rpoA
petD
petB
psbH
psbT
psbN
psbB
rps12
clpP
trnR-UCU
atpA
atpF
atpH
atpI
rps2
rpoC2
rpoC1
rpoB
trnC-GCA
petN
psbM
trnD-GUC
trnY-GUA
trnE-UUC
trnT-GGU
psbD
psbC
psbZ
trnG-GCC
photosystem I
photosystem II
cytochrome b/f complex
ATP synthase
RubisCO large subunit
RNA polymerase
ribosomal proteins (SSU)
ribosomal proteins (LSU)
transfer RNAs
ribosomal RNAs
clpP, matK
other genes
hypothetical chloroplast reading frames (ycf)

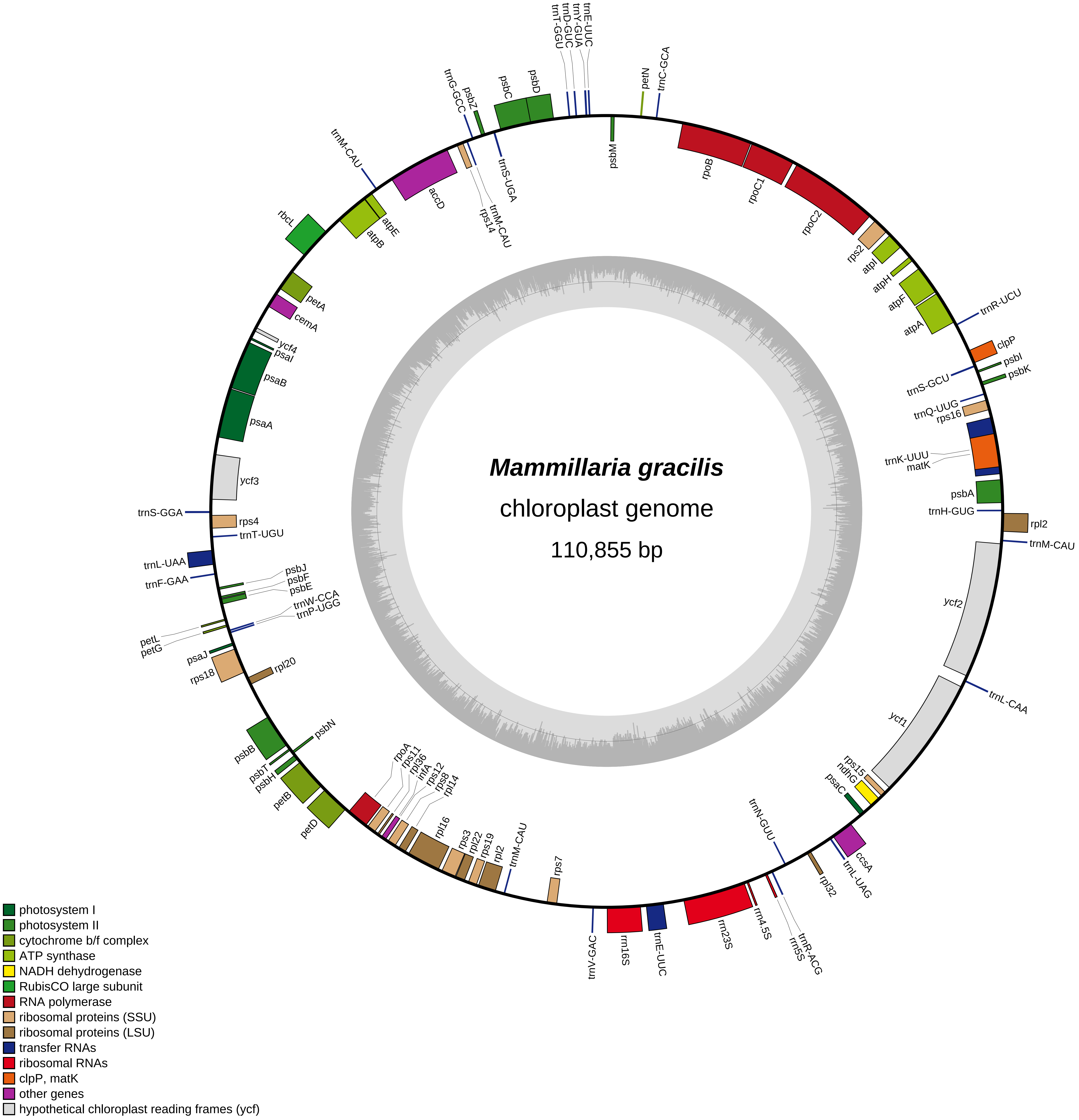

Mammillaria gracilis
chloroplast genome
110,855 bp
photosystem I
photosystem II
cytochrome b/f complex
ATP synthase
NADH dehydrogenase
RubisCO large subunit
RNA polymerase
ribosomal proteins (SSU)
ribosomal proteins (LSU)
transfer RNAs
ribosomal RNAs
clpP, matK
other genes
hypothetical chloroplast reading frames (ycf)
trnT-GGU
trnD-GUC
trnY-GUA
trnE-UUC
petN
trnC-GCA
psbM
rpoB
rpoC1
rpoC2
rps2
atpI
atpH
atpF
atpA
trnR-UCU
clpP
psbI
psbK
trnS-GCU
trnQ-UUG
rps16
trnK-UUU
matK
psbA
trnH-GUG
rpl2
trnM-CAU
ycf2
trnL-CAA
ycf1
rps15
ndhG
psaC
ccsA
trnL-UAG
rpl32
trnN-GUU
trnR-ACG
rrn5S
rrn4.5S
rrn23S
trnE-UUC
rrn16S
trnV-GAC
rps7
trnM-CAU
rpl2
rps19
rpl22
rps3
rpl16
rpl14
rps8
rps12
infA
rpl36
rps11
rpoA
petD
petB
psbH
psbT
psbB
psbN
rpl20
rps18
psaJ
petG
petL
trnP-UGG
trnW-CCA
psbE
psbF
psbJ
trnF-GAA
trnL-UAA
trnT-UGU
rps4
trnS-GGA
ycf3
psaA
psaB
psaI
ycf4
cemA
petA
rbcL
atpB
atpE
trnM-CAU
accD
rps14
trnM-CAU
trnS-UGA
trnG-GCC
psbZ
psbC
psbD

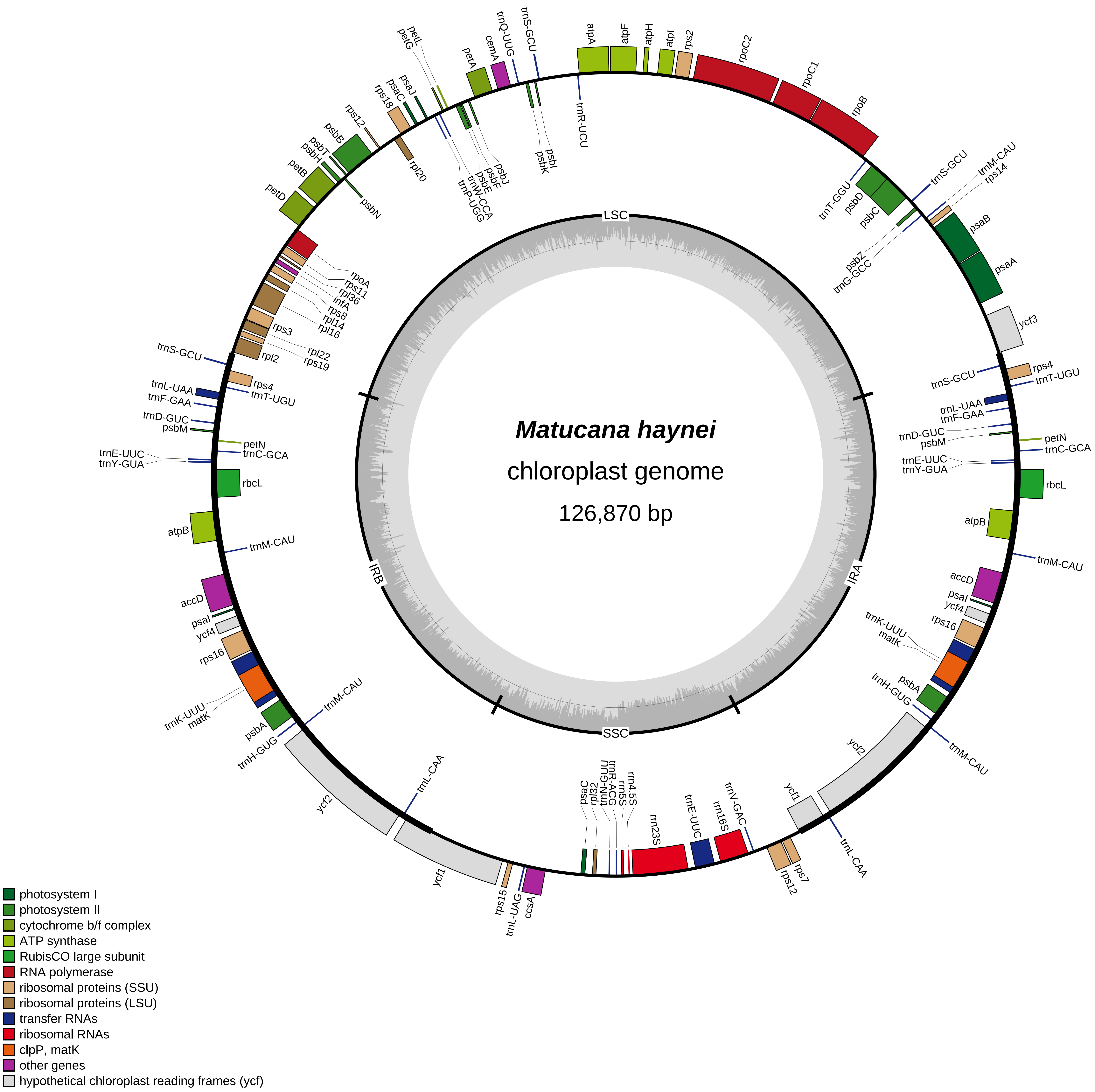
Matucana haynei
chloroplast genome
126,870 bp
LSC
IRA
SSC
IRB
atpA
atpF
atpH
atpI
rps2
rpoC2
rpoC1
rpoB
trnR-UCU
trnS-GCU
trnQ-UUG
cemA
petA
psaJ
psaC
rps18
rps12
psbB
psbT
psbH
petB
petD
petL
petG
psbI
psbK
psbJ
psbF
psbE
trnW-CCA
trnP-UGG
rpl20
psbN
trnT-GGU
psbD
psbC
trnS-GCU
trnM-CAU
rps14
psbZ
trnG-GCC
psaB
psaA
ycf3
rps4
trnT-UGU
trnL-UAA
trnF-GAA
trnD-GUC
psbM
petN
trnC-GCA
trnE-UUC
trnY-GUA
rbcL
atpB
trnM-CAU
accD
psaI
ycf4
rps16
trnK-UUU
matK
psbA
trnH-GUG
ycf2
ycf1
trnL-CAA
rps12
rps7
trnV-GAC
rrn16S
trnE-UUC
rrn23S
rrn4.5S
rrn5S
trnR-ACG
trnN-GUU
rpl32
psaC
ccsA
trnL-UAG
rps15
rpoA
rps11
rpl36
infA
rps8
rpl14
rpl16
rps3
rpl22
rps19
rpl2
photosystem I
photosystem II
cytochrome b/f complex
ATP synthase
RubisCO large subunit
RNA polymerase
ribosomal proteins (SSU)
ribosomal proteins (LSU)
transfer RNAs
ribosomal RNAs
clpP, matK
other genes
hypothetical chloroplast reading frames (ycf)

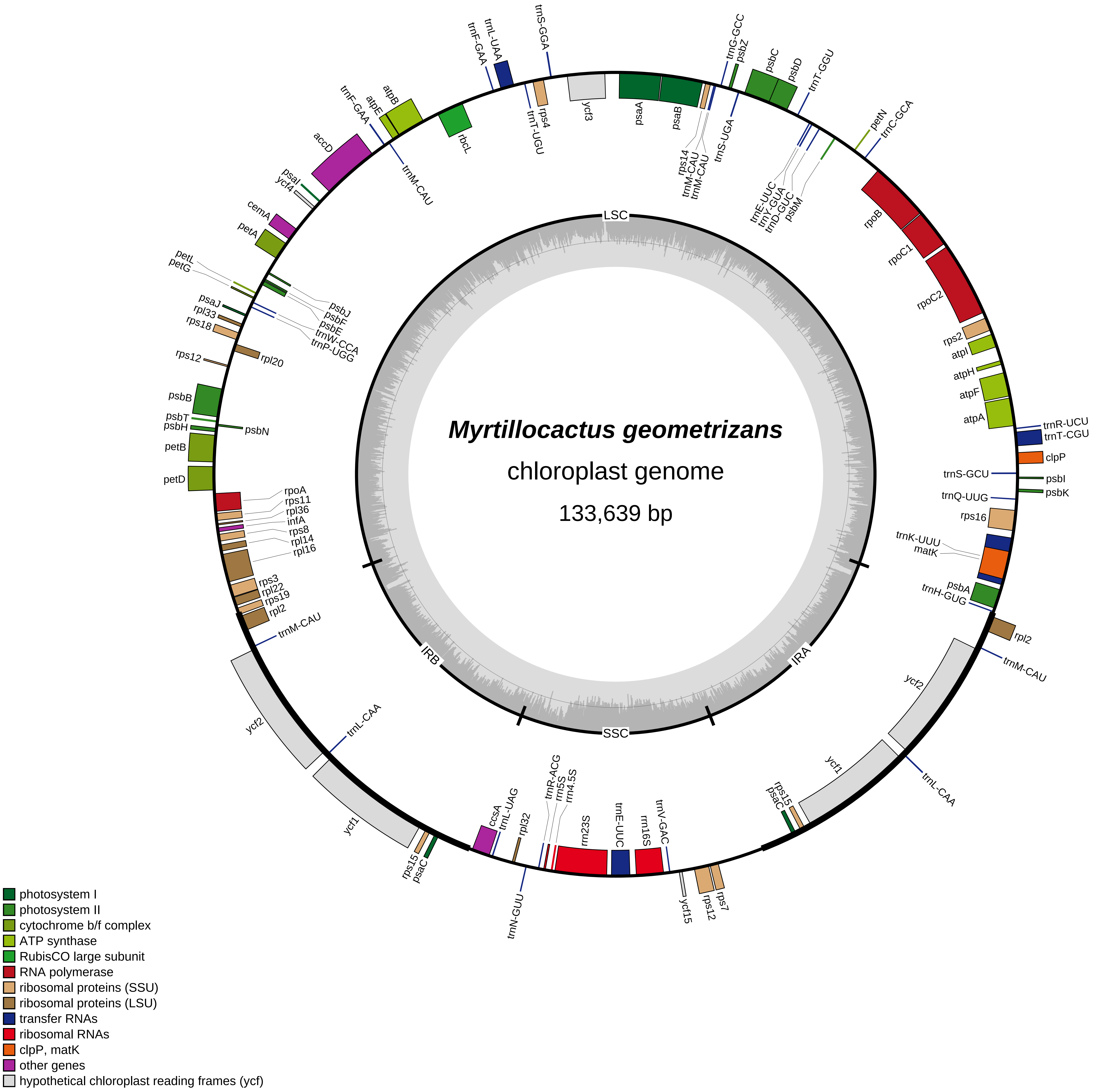

Myrtillocactus geometrizans
chloroplast genome
133,639 bp
LSC
IRA
SSC
IRB
psaA
psaB
ycf3
rps4
trnS-GGA
trnL-UAA
trnF-GAA
trnT-UGU
rbcL
atpB
atpE
trnF-GAA
trnM-CAU
accD
psaI
ycf4
cemA
petA
petL
petG
psbJ
psbF
psbE
trnW-CCA
trnP-UGG
psaJ
rpl33
rps18
rpl20
rps12
psbB
psbT
psbH
psbN
petB
petD
rpoA
rps11
rpl36
infA
rps8
rpl14
rpl16
rps3
rpl22
rps19
rpl2
trnM-CAU
ycf2
trnL-CAA
ycf1
rps15
psaC
ccsA
trnL-UAG
rpl32
trnN-GUU
trnR-ACG
rrn5S
rrn4.5S
rrn23S
trnE-UUC
rrn16S
trnV-GAC
ycf15
rps12
rps7
rps15
psaC
ycf1
trnL-CAA
ycf2
trnM-CAU
rpl2
trnH-GUG
psbA
matK
trnK-UUU
rps16
trnQ-UUG
trnS-GCU
psbI
psbK
clpP
trnR-UCU
trnT-CGU
atpA
atpF
atpH
atpI
rps2
rpoC2
rpoC1
rpoB
petN
trnC-GCA
trnE-UUC
trnY-GUA
trnD-GUC
psbM
trnT-GGU
psbD
psbC
psbZ
trnG-GCC
trnS-UGA
rps14
trnM-CAU
trnM-CAU
photosystem I
photosystem II
cytochrome b/f complex
ATP synthase
RubisCO large subunit
RNA polymerase
ribosomal proteins (SSU)
ribosomal proteins (LSU)
transfer RNAs
ribosomal RNAs
clpP, matK
other genes
hypothetical chloroplast reading frames (ycf)

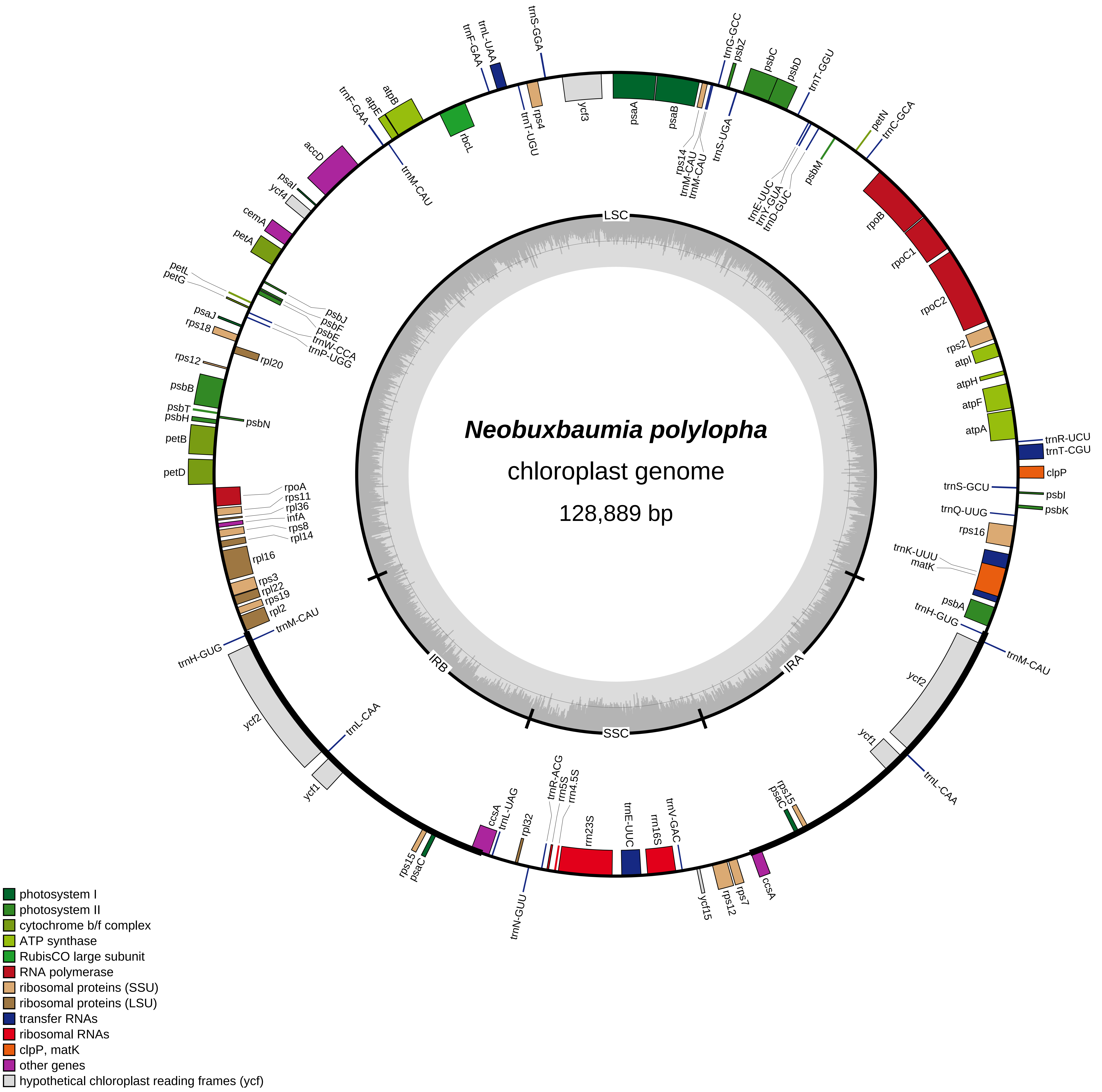
Neobuxbaumia polylopha
chloroplast genome
128,889 bp
LSC
IRB
IRA
SSC
trnS-GGA
trnL-UAA
trnF-GAA
trnT-UGU
rps4
ycf3
psaA
psaB
trnG-GCC
psbZ
psbC
psbD
trnT-GGU
rps14
trnM-CAU
trnfM-CAU
trnS-UGA
trnE-UUC
trnY-GUA
trnD-GUC
psbM
petN
trnC-GCA
rpoB
rpoC1
rpoC2
rps2
atpI
atpH
atpF
atpA
trnR-UCU
trnT-CGU
clpP
trnS-GCU
psbI
trnQ-UUG
psbK
rps16
trnK-UUU
matK
psbA
trnH-GUG
trnM-CAU
ycf2
ycf1
trnL-CAA
rps15
psaC
ccsA
rps7
rps12
ycf15
trnV-GAC
rrn16S
trnE-UUC
rrn23S
rrn4.5S
rrn5S
trnR-ACG
rpl32
trnL-UAG
ccsA
trnN-GUU
psaC
rps15
ycf1
trnL-CAA
ycf2
trnH-GUG
trnM-CAU
rpl2
rps19
rpl22
rps3
rpl16
rpl14
rps8
infA
rpl36
rps11
rpoA
petD
petB
psbH
psbT
psbN
psbB
rps12
rpl20
rps18
psaJ
trnP-UGG
trnW-CCA
psbE
psbF
psbJ
petG
petL
petA
cemA
ycf4
psaI
accD
trnM-CAU
atpE
atpB
rbcL
photosystem I
photosystem II
cytochrome b/f complex
ATP synthase
RubisCO large subunit
RNA polymerase
ribosomal proteins (SSU)
ribosomal proteins (LSU)
transfer RNAs
ribosomal RNAs
clpP, matK
other genes
hypothetical chloroplast reading frames (ycf)

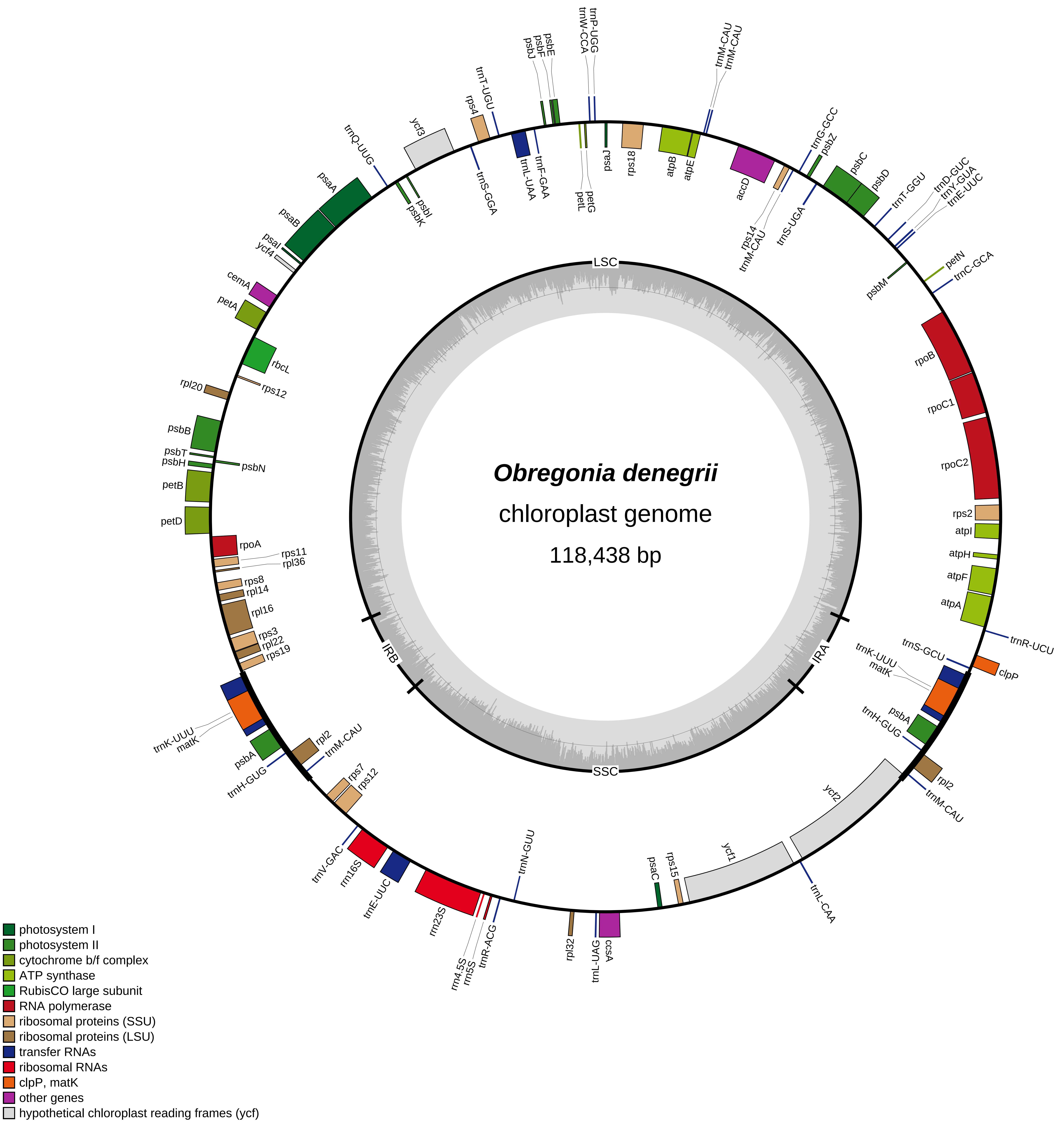
Obregonia denegrii
chloroplast genome
118,438 bp
LSC
IRA
SSC
IRB
trnP-UGG
trnW-CCA
psbE
psbF
psbJ
trnT-UGU
rps4
ycf3
trnQ-UUG
psaA
psaB
psaI
ycf4
cemA
petA
rbcL
rps12
rpl20
psbB
psbT
psbH
psbN
petB
petD
rpoA
rps11
rpl36
rps8
rpl14
rpl16
rps3
rpl22
rps19
trnK-UUU
matK
psbA
trnH-GUG
rpl2
trnM-CAU
rps7
rps12
trnV-GAC
rrn16S
trnE-UUC
rrn23S
rrn4.5S
rrn5S
trnR-ACG
trnN-GUU
rpl32
trnL-UAG
ccsA
psaC
rps15
ycf1
trnL-CAA
ycf2
rpl2
trnM-CAU
trnH-GUG
psbA
trnK-UUU
matK
trnS-GCU
clpP
trnR-UCU
atpA
atpF
atpH
atpI
rps2
rpoC2
rpoC1
rpoB
petN
trnC-GCA
psbM
trnD-GUC
trnY-GUA
trnE-UUC
trnT-GGU
psbD
psbC
trnS-UGA
psbZ
trnG-GCC
rps14
trnM-CAU
accD
atpE
atpB
rps18
psaJ
petG
petL
trnF-GAA
trnL-UAA
trnS-GGA
psbI
psbK
trnM-CAU
trnM-CAU
photosystem I
photosystem II
cytochrome b/f complex
ATP synthase
RubisCO large subunit
RNA polymerase
ribosomal proteins (SSU)
ribosomal proteins (LSU)
transfer RNAs
ribosomal RNAs
clpP, matK
other genes
hypothetical chloroplast reading frames (ycf)

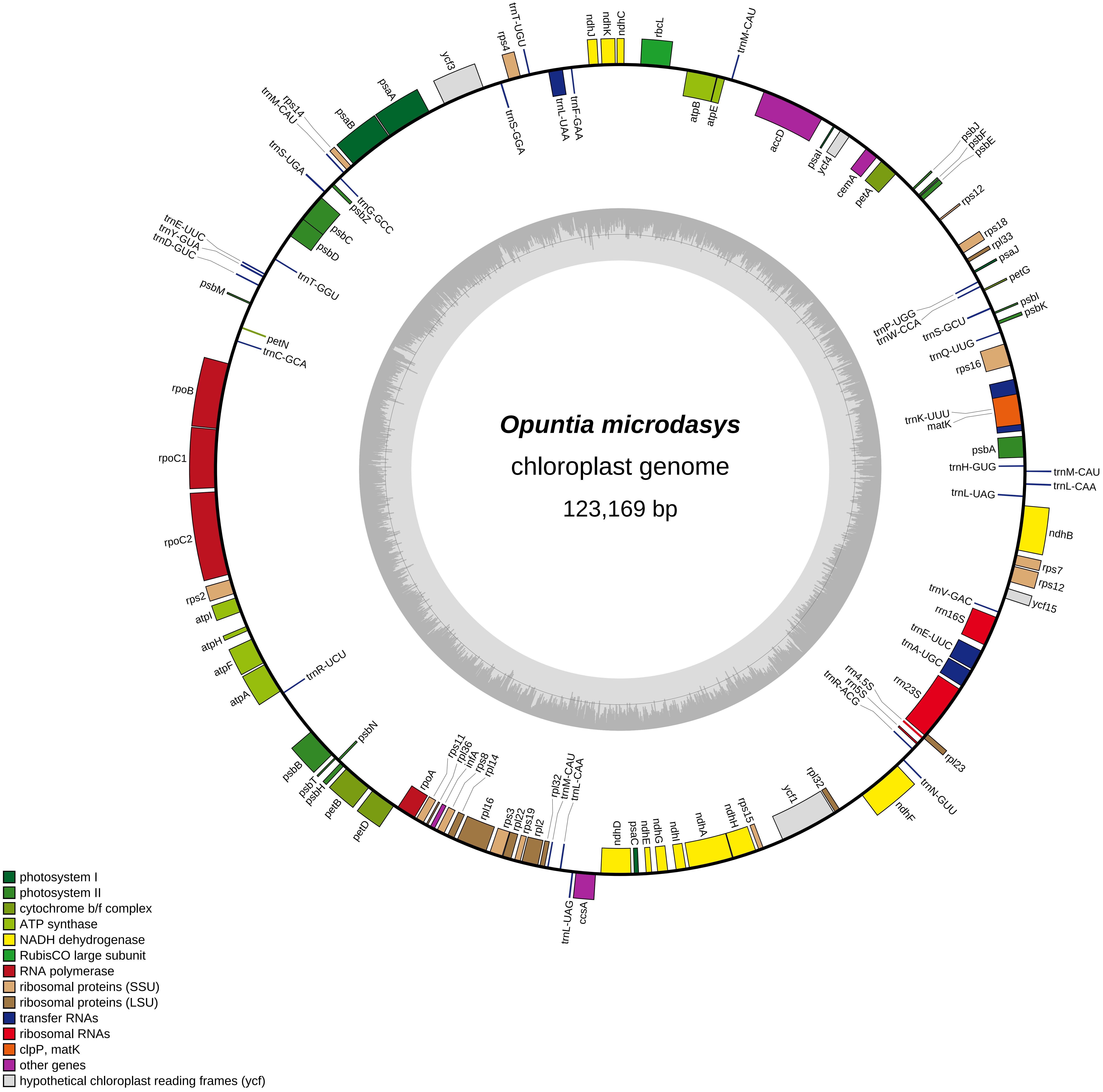

Opuntia microdasys
chloroplast genome
123,169 bp
trnT-UGU
rps4
ycf3
psaA
psaB
rps14
trnM-CAU
trnS-UGA
trnS-GGA
trnL-UAA
trnF-GAA
ndhJ
ndhK
ndhC
rbcL
trnM-CAU
atpB
atpE
accD
psaI
ycf4
cemA
petA
psbJ
psbL
psbF
psbE
rps12
rps18
rpl33
psaJ
petG
psbI
psbK
trnP-UGG
trnW-CCA
trnS-GCU
trnQ-UUG
rps16
trnK-UUU
matK
psbA
trnH-GUG
trnM-CAU
trnL-CAA
trnL-UAG
ndhB
rps7
rps12
ycf15
trnV-GAC
rrn16S
trnE-UUC
trnA-UGC
rrn23S
rrn4.5S
rrn5S
trnR-ACG
rpl23
trnN-GUU
ndhF
rpl32
ycf1
rps15
ndhH
ndhA
ndhI
ndhG
ndhE
psaC
ndhD
ccsA
trnL-UAG
trnL-CAA
trnM-CAU
rpl32
rpl2
rps19
rpl22
rps3
rpl16
rpl14
rps8
infA
rpl36
rps11
rpoA
petD
petB
psbH
psbT
psbB
psbN
trnR-UCU
atpA
atpF
atpH
atpI
rps2
rpoC2
rpoC1
rpoB
trnC-GCA
petN
psbM
trnE-UUC
trnY-GUA
trnD-GUC
trnT-GGU
psbD
psbC
psbZ
trnG-GCC
photosystem I
photosystem II
cytochrome b/f complex
ATP synthase
NADH dehydrogenase
RubisCO large subunit
RNA polymerase
ribosomal proteins (SSU)
ribosomal proteins (LSU)
transfer RNAs
ribosomal RNAs
clpP, matK
other genes
hypothetical chloroplast reading frames (ycf)

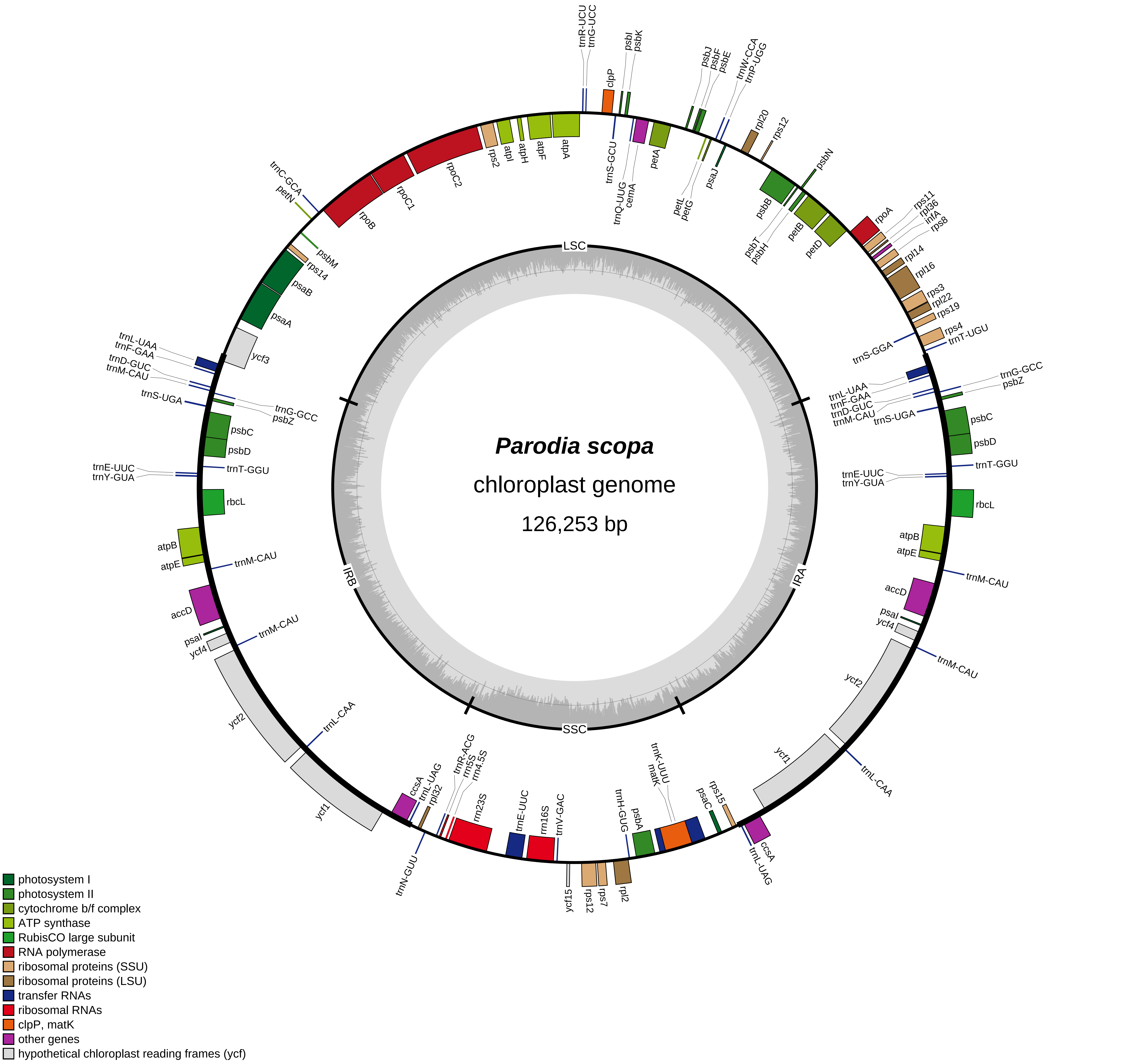

Parodia scopa
chloroplast genome
126,253 bp
LSC
IRA
SSC
IRB
trnR-UCU
trnG-UCC
clpP
psbI
psbK
psbJ
psbF
psbE
trnW-CCA
trnP-UGG
rpl20
rps12
psbN
trnS-GCU
trnQ-UUG
cemA
petA
petL
petG
psaJ
psbB
psbT
psbH
petB
petD
rpoA
rps11
rpl36
infA
rps8
rpl14
rpl16
rps3
rpl22
rps19
rps4
trnT-UGU
trnS-GGA
trnC-GCA
petN
rpoB
rpoC1
rpoC2
rps2
atpI
atpH
atpF
atpA
psbM
rps14
psaB
psaA
ycf3
trnL-UAA
trnF-GAA
trnD-GUC
trnM-CAU
trnS-UGA
trnG-GCC
psbZ
psbC
psbD
trnE-UUC
trnY-GUA
trnT-GGU
rbcL
atpB
atpE
accD
psaI
ycf4
ycf2
trnL-CAA
ycf1
ccsA
trnL-UAG
rpl32
trnN-GUU
trnR-ACG
rrn5S
rrn4.5S
rrn23S
trnE-UUC
rrn16S
trnV-GAC
ycf15
rps12
rps7
rpl2
trnH-GUG
psbA
trnK-UUU
matK
psaC
rps15
photosystem I
photosystem II
cytochrome b/f complex
ATP synthase
RubisCO large subunit
RNA polymerase
ribosomal proteins (SSU)
ribosomal proteins (LSU)
transfer RNAs
ribosomal RNAs
clpP, matK
other genes
hypothetical chloroplast reading frames (ycf)

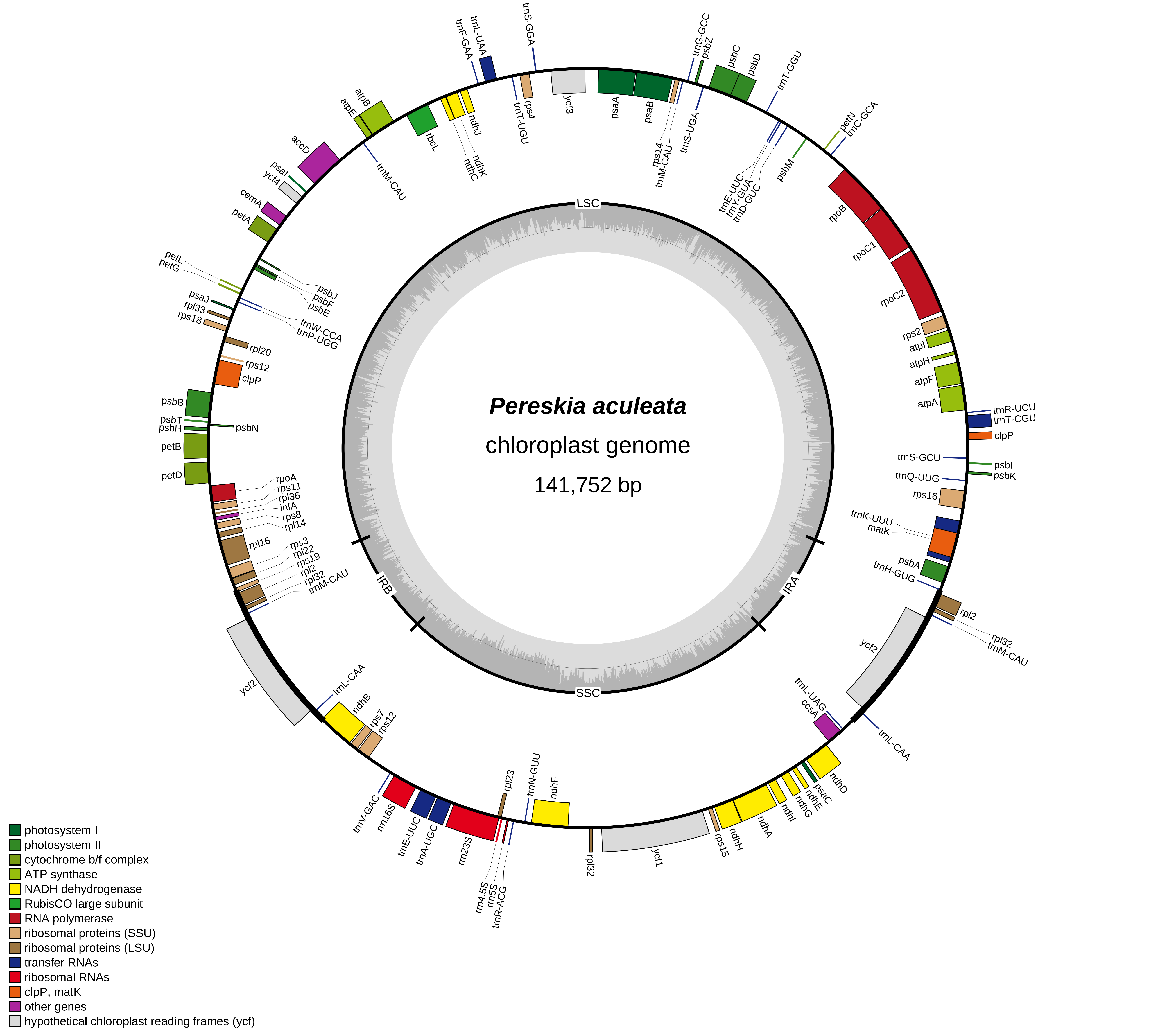
Pereskia aculeata
chloroplast genome
141,752 bp
LSC
IRA
SSC
IRB
trnS-GGA
trnL-UAA
trnF-GAA
trnG-GCC
psbZ
psbC
psbD
trnT-GGU
petN
trnC-GCA
psbM
trnE-UUC
trnY-GUA
trnD-GUC
trnS-UGA
rps14
trnM-CAU
psaA
psaB
ycf3
rps4
trnT-UGU
ndhJ
ndhK
ndhC
rbcL
atpB
atpE
trnM-CAU
accD
psaI
ycf4
cemA
petA
petL
petG
psbJ
psbF
psbE
psaJ
rpl33
rps18
trnW-CCA
trnP-UGG
rpl20
rps12
clpP
psbB
psbT
psbH
psbN
petB
petD
rpoA
rps11
rpl36
infA
rps8
rpl14
rpl16
rps3
rpl22
rps19
rpl2
rpl32
trnM-CAU
ycf2
trnL-CAA
ndhB
rps7
rps12
trnV-GAC
rrn16S
trnE-UUC
trnA-UGC
rrn23S
rrn4.5S
rrn5S
trnR-ACG
rpl23
trnN-GUU
ndhF
rpl32
ycf1
rps15
ndhH
ndhA
ndhI
ndhG
ndhE
psaC
ndhD
ccsA
trnL-UAG
trnL-CAA
ycf2
rpl2
rpl32
trnM-CAU
trnH-GUG
psbA
matK
trnK-UUU
rps16
trnQ-UUG
trnS-GCU
psbI
psbK
clpP
trnR-UCU
trnT-CGU
atpA
atpF
atpH
atpI
rps2
rpoC2
rpoC1
rpoB
photosystem I
photosystem II
cytochrome b/f complex
ATP synthase
NADH dehydrogenase
RubisCO large subunit
RNA polymerase
ribosomal proteins (SSU)
ribosomal proteins (LSU)
transfer RNAs
ribosomal RNAs
clpP, matK
other genes
hypothetical chloroplast reading frames (ycf)

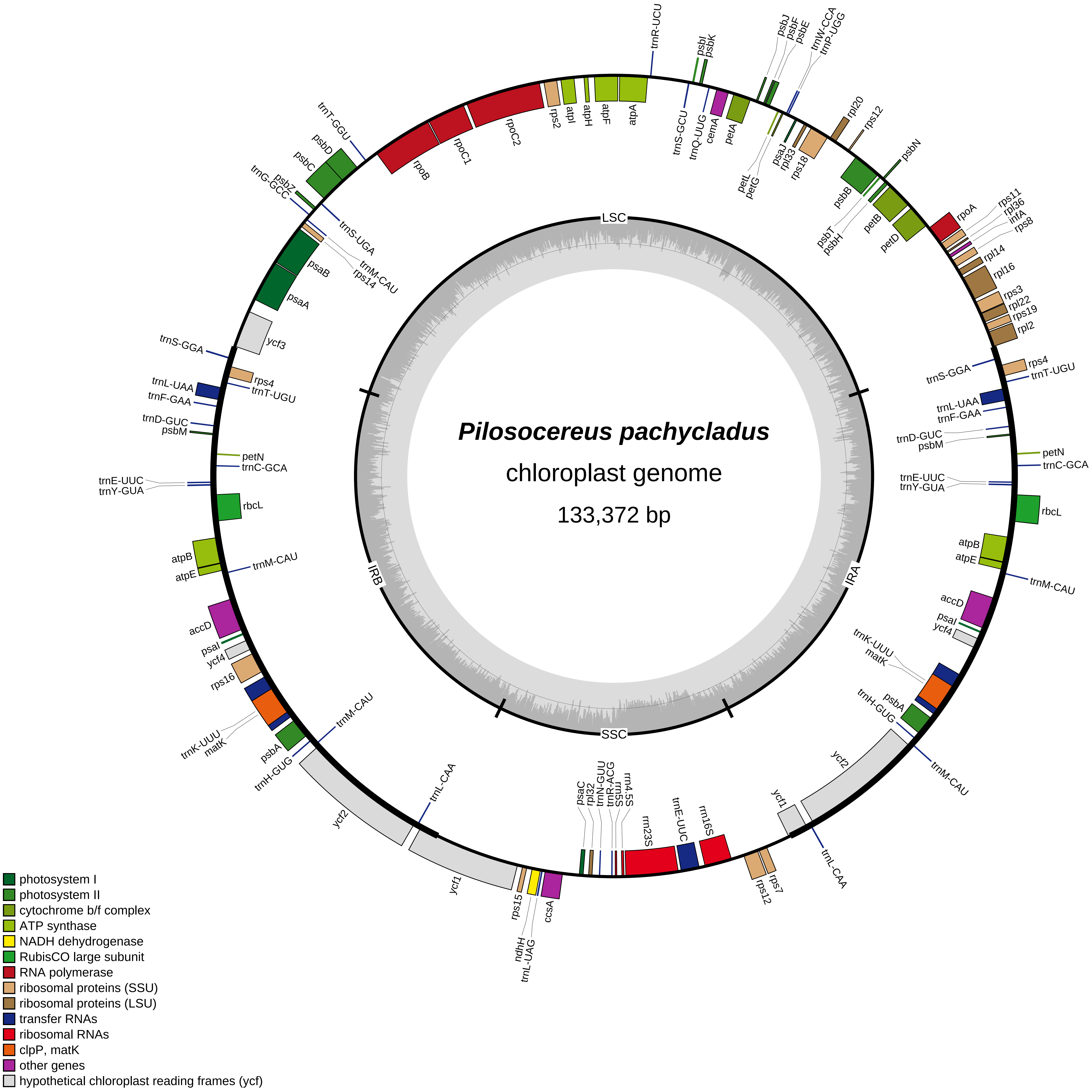
Pilosocereus pachycladus
chloroplast genome
133,372 bp
LSC
IRA
SSC
IRB
trnR-UCU
psbI
psbK
psbJ
psbF
psbE
trnW-CCA
trnP-UGG
rpl20
rps12
psbN
rpoA
rps11
rpl36
infA
rps8
rpl14
rpl16
rps3
rpl22
rps19
rpl2
rps4
trnT-UGU
trnS-GGA
trnL-UAA
trnF-GAA
trnD-GUC
psbM
petN
trnC-GCA
trnE-UUC
trnY-GUA
rbcL
atpB
atpE
trnM-CAU
accD
psaI
ycf4
trnK-UUU
matK
psbA
trnH-GUG
ycf2
ycf1
trnL-CAA
rps7
rrn16S
trnE-UUC
rrn23S
rrn4.5S
rrn5S
trnR-ACG
trnN-GUU
rpl32
psaC
ccsA
ndhH
trnL-UAG
rps15
rps16
ycf3
psaA
psaB
rps14
trnM-CAU
trnS-UGA
trnG-GCC
psbZ
psbC
psbD
trnT-GGU
rpoB
rpoC1
rpoC2
rps2
atpI
atpH
atpF
atpA
trnS-GCU
trnQ-UUG
cemA
petA
petL
petG
psaJ
rpl33
rps18
psbB
psbT
psbH
petB
petD
photosystem I
photosystem II
cytochrome b/f complex
ATP synthase
NADH dehydrogenase
RubisCO large subunit
RNA polymerase
ribosomal proteins (SSU)
ribosomal proteins (LSU)
transfer RNAs
ribosomal RNAs
clpP, matK
other genes
hypothetical chloroplast reading frames (ycf)

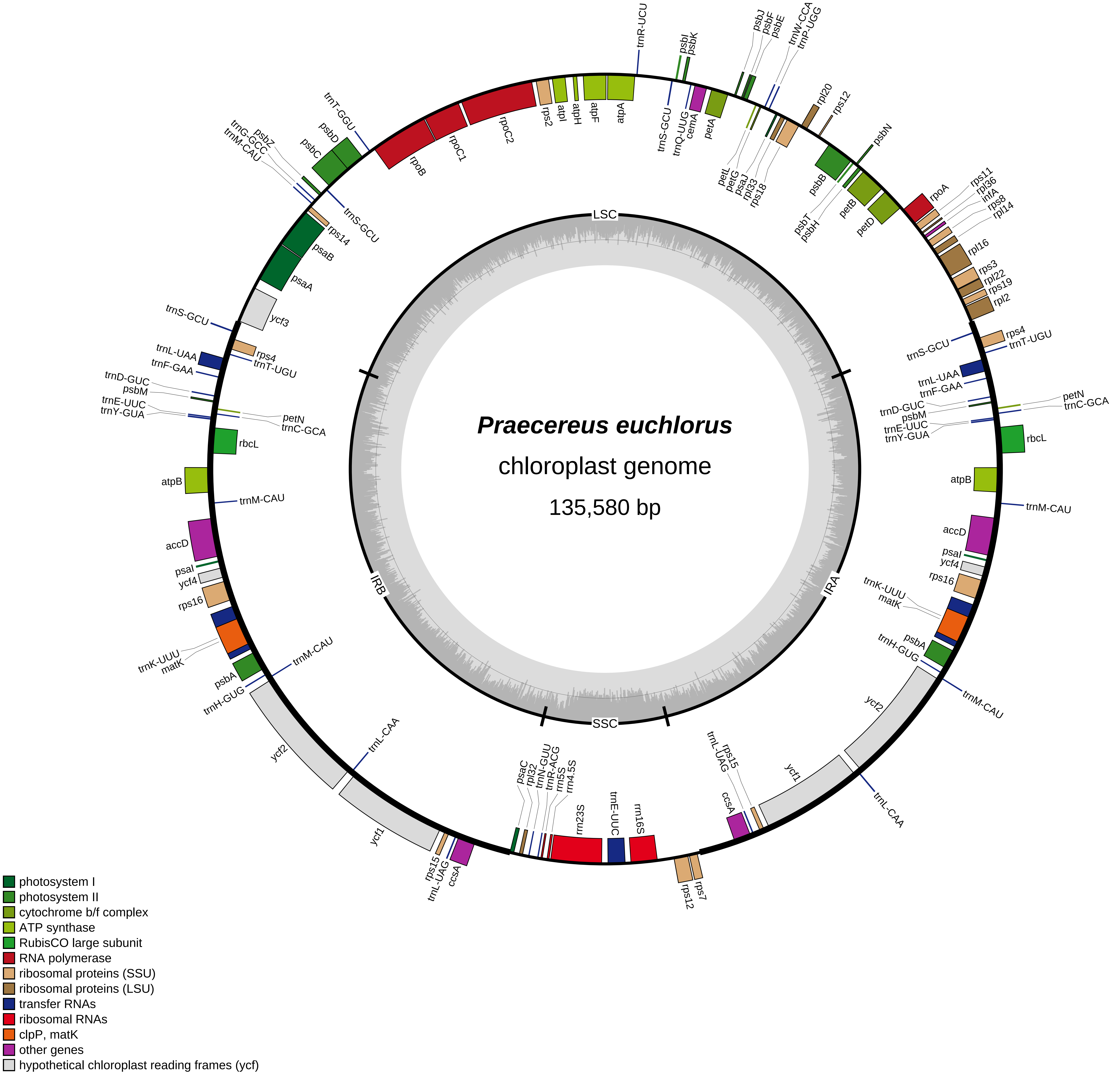
Praecereus euchlorus
chloroplast genome
135,580 bp
LSC
SSC
IRA
IRB
photosystem I
photosystem II
cytochrome b/f complex
ATP synthase
RubisCO large subunit
RNA polymerase
ribosomal proteins (SSU)
ribosomal proteins (LSU)
transfer RNAs
ribosomal RNAs
clpP, matK
other genes
hypothetical chloroplast reading frames (ycf)

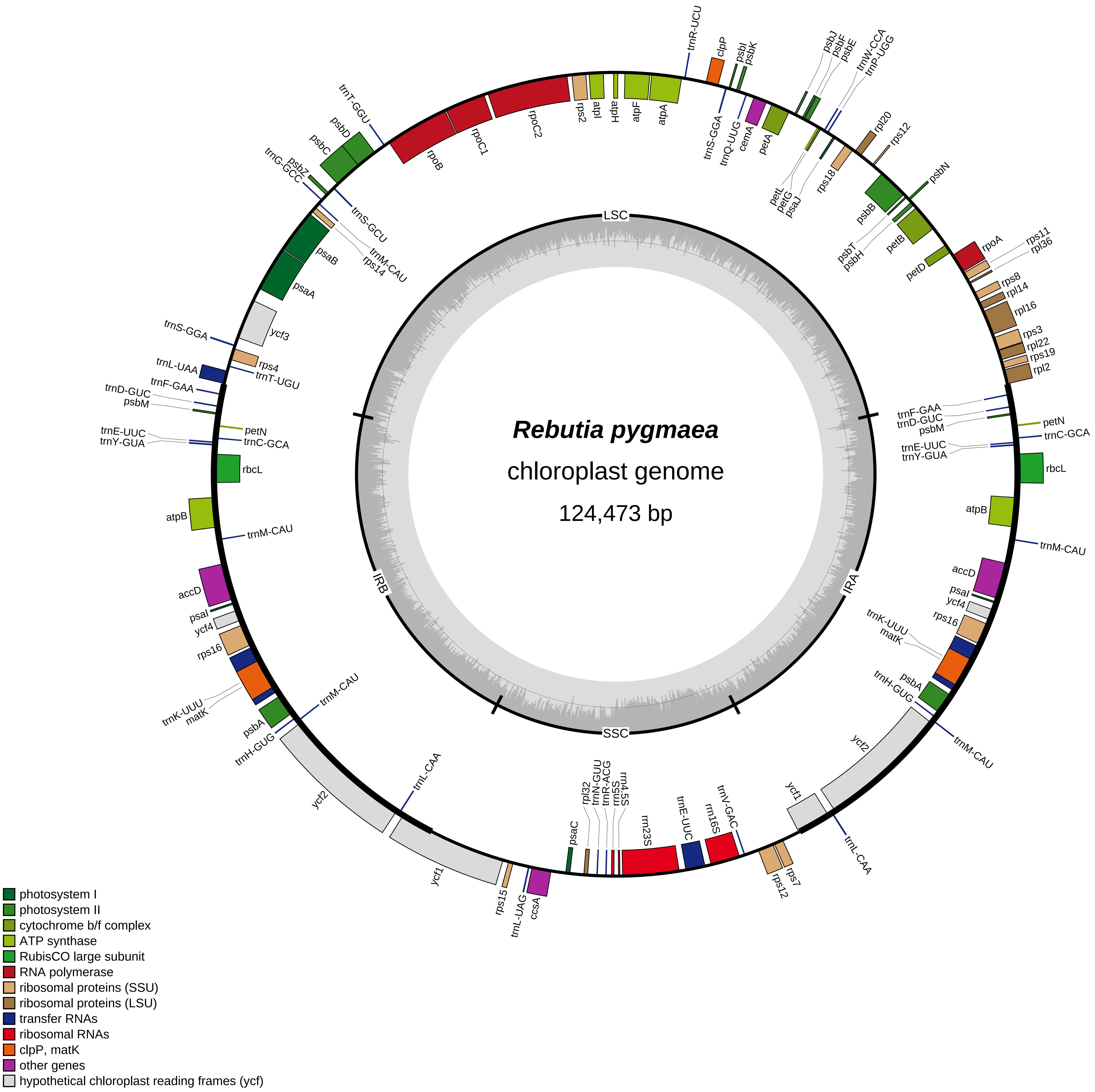

Rebutia pygmaea
chloroplast genome
124,473 bp
LSC
IRA
SSC
IRB
trnR-UCU
clpP
psbI
psbK
psbJ
psbF
psbE
trnW-CCA
trnP-UGG
rpl20
rps12
psbN
rpoA
rps11
rpl36
rps8
rpl14
rpl16
rps3
rpl22
rps19
rpl2
trnF-GAA
trnD-GUC
psbM
petN
trnC-GCA
trnE-UUC
trnY-GUA
rbcL
atpB
trnM-CAU
accD
psaI
ycf4
rps16
trnK-UUU
matK
psbA
trnH-GUG
trnM-CAU
ycf2
ycf1
trnL-CAA
rps7
rps12
trnV-GAC
rrn16S
trnE-UUC
rrn23S
rrn4.5S
rrn5S
trnR-ACG
trnN-GUU
rpl32
psaC
ccsA
trnL-UAG
rps15
ycf1
ycf2
trnL-CAA
trnM-CAU
trnH-GUG
psbA
trnK-UUU
matK
rps16
ycf4
psaI
accD
trnM-CAU
atpB
rbcL
trnE-UUC
trnY-GUA
petN
trnC-GCA
trnD-GUC
psbM
trnF-GAA
trnL-UAA
trnT-UGU
rps4
trnS-GGA
ycf3
psaA
psaB
rps14
trnM-CAU
trnS-GCU
trnG-GCC
psbZ
psbC
psbD
trnT-GGU
rpoB
rpoC1
rpoC2
rps2
atpI
atpH
atpF
atpA
trnS-GGA
trnQ-UUG
cemA
petA
petL
petG
psaJ
rps18
psbB
psbT
psbH
petB
petD
photosystem I
photosystem II
cytochrome b/f complex
ATP synthase
RubisCO large subunit
RNA polymerase
ribosomal proteins (SSU)
ribosomal proteins (LSU)
transfer RNAs
ribosomal RNAs
clpP, matK
other genes
hypothetical chloroplast reading frames (ycf)

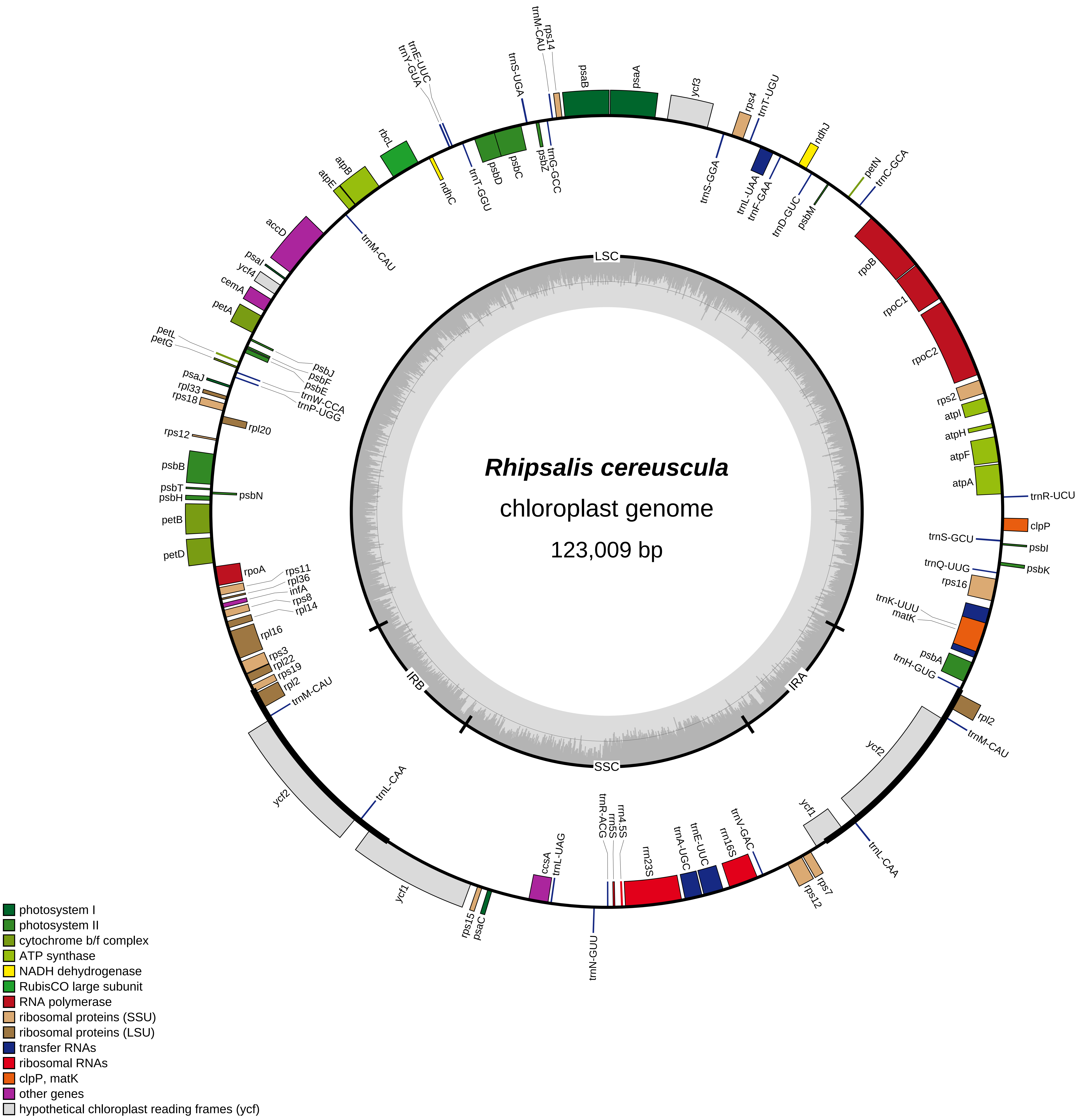

Rhipsalis cereuscula
chloroplast genome
123,009 bp
LSC
SSC
IRA
IRB
psaB
psaA
ycf3
rps4
trnT-UGU
trnS-GGA
trnL-UAA
trnF-GAA
ndhJ
trnD-GUC
psbM
petN
trnC-GCA
rpoB
rpoC1
rpoC2
rps2
atpI
atpH
atpF
atpA
trnR-UCU
clpP
trnS-GCU
psbI
trnQ-UUG
psbK
rps16
trnK-UUU
matK
psbA
trnH-GUG
rpl2
trnM-CAU
ycf2
ycf1
trnL-CAA
rps7
rps12
trnV-GAC
rrn16S
trnE-UUC
trnA-UGC
rrn23S
rrn4.5S
rrn5S
trnR-ACG
trnN-GUU
trnL-UAG
ccsA
psaC
rps15
ycf1
ycf2
trnL-CAA
trnM-CAU
rpl2
rps19
rpl22
rps3
rpl16
rpl14
rps8
infA
rpl36
rps11
rpoA
petD
petB
psbH
psbT
psbN
psbB
rps12
rpl20
rps18
rpl33
psaJ
trnP-UGG
trnW-CCA
psbE
psbF
psbJ
petG
petL
petA
cemA
ycf4
psaI
accD
atpE
atpB
trnM-CAU
rbcL
ndhC
trnT-GGU
trnY-GUA
trnE-UUC
trnS-UGA
psbD
psbC
psbZ
trnG-GCC
trnM-CAU
rps14
photosystem I
photosystem II
cytochrome b/f complex
ATP synthase
NADH dehydrogenase
RubisCO large subunit
RNA polymerase
ribosomal proteins (SSU)
ribosomal proteins (LSU)
transfer RNAs
ribosomal RNAs
clpP, matK
other genes
hypothetical chloroplast reading frames (ycf)

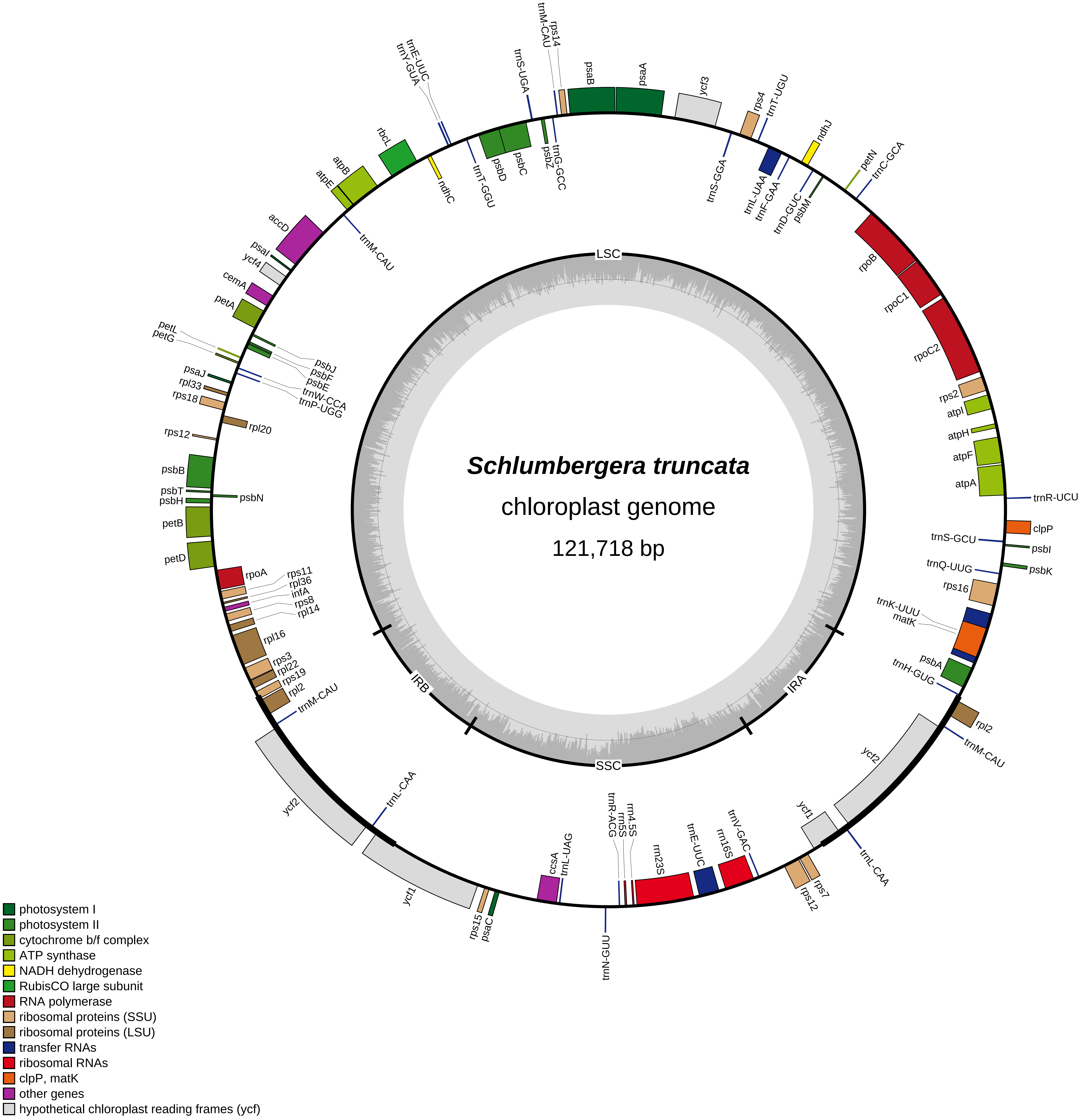

Schlumbergera truncata
chloroplast genome
121,718 bp
LSC
IRA
SSC
IRB
psaB
psaA
ycf3
rps4
trnT-UGU
ndhJ
petN
trnC-GCA
rps14
trnM-CAU
trnS-UGA
trnE-UUC
trnY-GUA
rbcL
atpB
atpE
accD
psaI
ycf4
cemA
petA
petL
petG
psbJ
psbF
psbE
trnW-CCA
trnP-UGG
psaJ
rpl33
rps18
rpl20
rps12
psbB
psbT
psbH
psbN
petB
petD
rpoA
rps11
rpl36
infA
rps8
rpl14
rpl16
rps3
rpl22
rps19
rpl2
trnM-CAU
ycf2
trnL-CAA
ycf1
rps15
psaC
ccsA
trnL-UAG
trnN-GUU
trnR-ACG
rrn5S
rrn4.5S
rrn23S
trnE-UUC
rrn16S
trnV-GAC
rps12
rps7
ycf1
trnL-CAA
ycf2
trnM-CAU
rpl2
trnH-GUG
psbA
matK
trnK-UUU
rps16
trnQ-UUG
psbK
psbI
trnS-GCU
clpP
trnR-UCU
atpA
atpF
atpH
atpI
rps2
rpoC2
rpoC1
rpoB
psbM
trnD-GUC
trnF-GAA
trnL-UAA
trnS-GGA
psbZ
trnG-GCC
psbC
psbD
trnT-GGU
ndhC
trnM-CAU
photosystem I
photosystem II
cytochrome b/f complex
ATP synthase
NADH dehydrogenase
RubisCO large subunit
RNA polymerase
ribosomal proteins (SSU)
ribosomal proteins (LSU)
transfer RNAs
ribosomal RNAs
clpP, matK
other genes
hypothetical chloroplast reading frames (ycf)

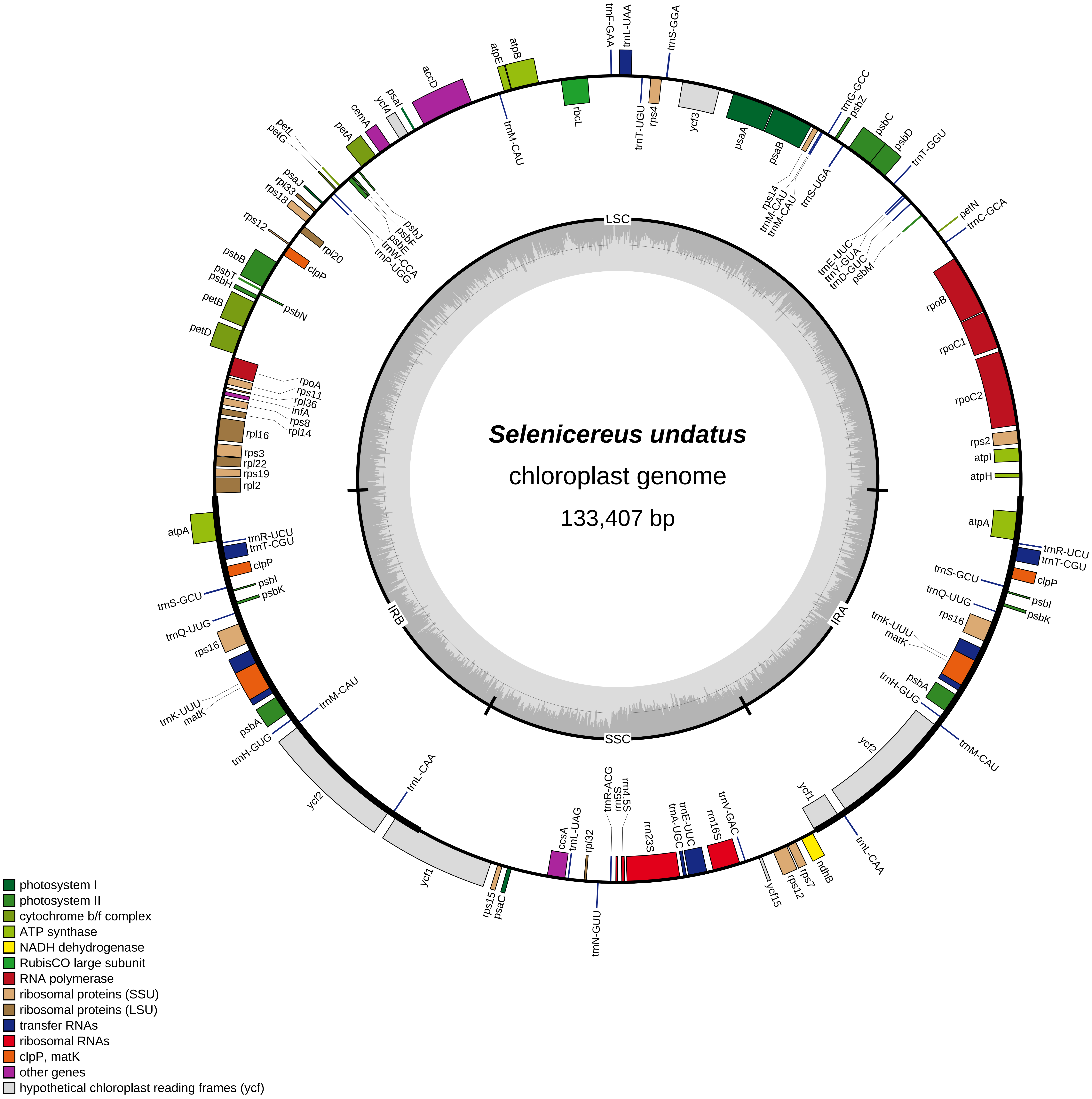
Selenicereus undatus
chloroplast genome
133,407 bp
LSC
IRA
SSC
IRB
trnF-GAA
trnL-UAA
trnS-GGA
atpE
atpB
accD
psaI
ycf4
cemA
petA
petL
petG
psaJ
rpl33
rps18
rps12
rpl20
clpP
psbB
psbT
psbH
psbN
petB
petD
rpoA
rps11
rpl36
infA
rps8
rpl14
rpl16
rps3
rpl22
rps19
rpl2
psbJ
psbL
psbF
psbE
trnW-CCA
trnP-UGG
trnM-CAU
rbcL
trnT-UGU
rps4
ycf3
psaA
psaB
rps14
trnfM-CAU
trnS-UGA
trnG-GCC
psbZ
psbC
psbD
trnT-GGU
trnE-UUC
trnY-GUA
trnD-GUC
psbM
petN
trnC-GCA
rpoB
rpoC1
rpoC2
rps2
atpI
atpH
atpA
trnR-UCU
trnT-CGU
clpP
trnS-GCU
psbI
trnQ-UUG
psbK
rps16
trnK-UUU
matK
psbA
trnH-GUG
trnM-CAU
ycf2
ycf1
trnL-CAA
ndhB
rps7
rps12
ycf15
trnV-GAC
rrn16S
trnE-UUC
trnA-UGC
rrn23S
rrn4.5S
rrn5S
trnR-ACG
trnN-GUU
rpl32
trnL-UAG
ccsA
psaC
rps15
photosystem I
photosystem II
cytochrome b/f complex
ATP synthase
NADH dehydrogenase
RubisCO large subunit
RNA polymerase
ribosomal proteins (SSU)
ribosomal proteins (LSU)
transfer RNAs
ribosomal RNAs
clpP, matK
other genes
hypothetical chloroplast reading frames (ycf)

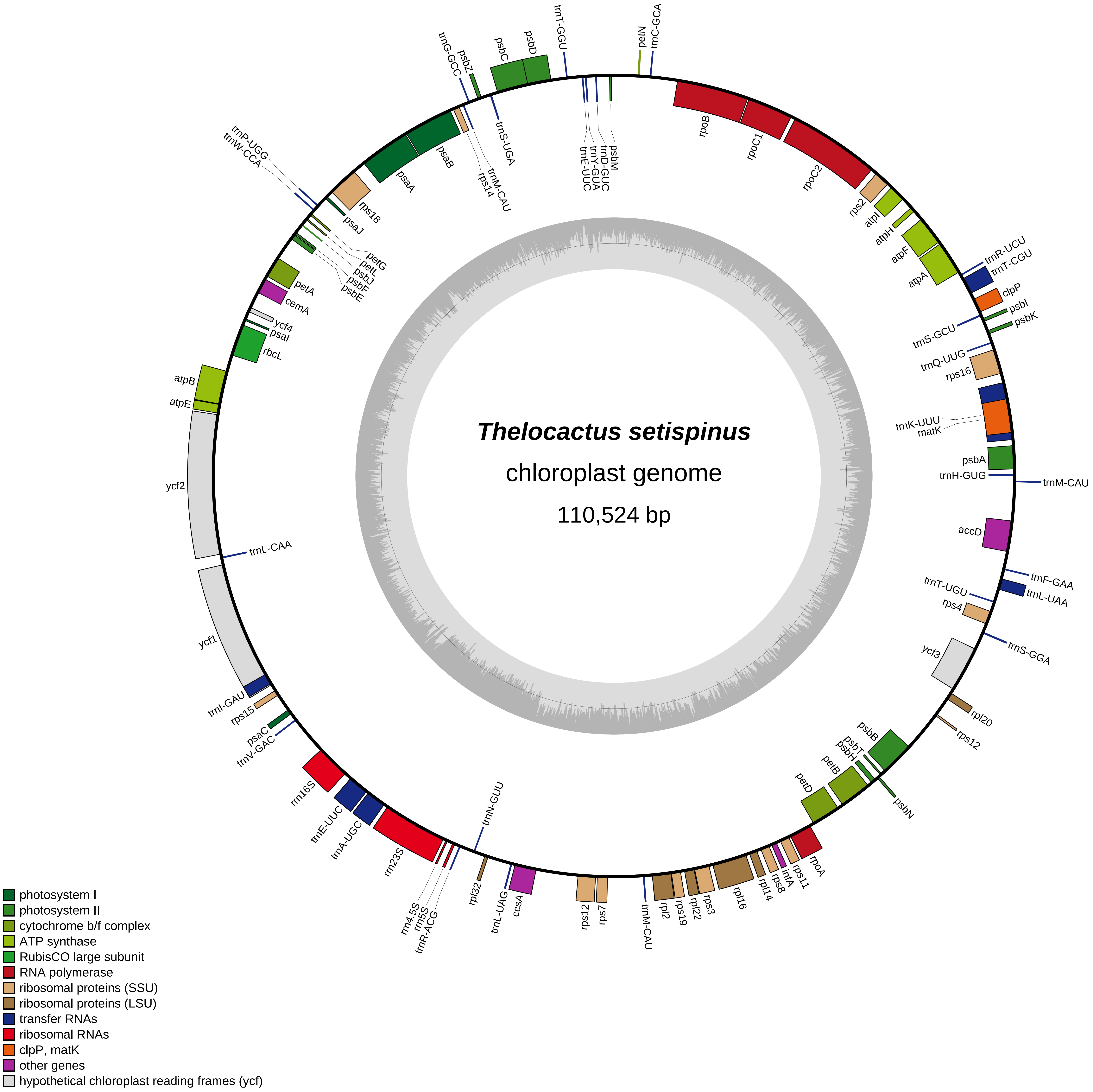
Thelocactus setispinus
chloroplast genome
110,524 bp
trnT-GGU
petN
trnC-GCA
psbD
psbC
psbZ
trnG-GCC
trnS-UGA
psbM
trnD-GUC
trnY-GUA
trnE-UUC
rpoB
rpoC1
rpoC2
rps2
atpI
atpH
atpF
atpA
trnR-UCU
trnT-CGU
clpP
psbI
psbK
trnS-GCU
trnQ-UUG
rps16
trnK-UUU
matK
psbA
trnH-GUG
trnM-CAU
accD
trnF-GAA
trnL-UAA
trnT-UGU
rps4
ycf3
trnS-GGA
rpl20
rps12
psbB
psbT
psbH
petB
psbN
petD
rpoA
rps11
infA
rps8
rpl14
rpl16
rps3
rpl22
rps19
rpl2
trnM-CAU
rps7
rps12
ccsA
trnL-UAG
rpl32
trnN-GUU
trnR-ACG
rrn5S
rrn4.5S
rrn23S
trnA-UGC
trnE-UUC
rrn16S
trnV-GAC
psaC
rps15
trnI-GAU
ycf1
trnL-CAA
ycf2
atpE
atpB
rbcL
psaI
ycf4
cemA
petA
psbE
psbF
psbJ
petL
petG
psaJ
rps18
trnW-CCA
trnP-UGG
psaA
psaB
rps14
trnM-CAU
photosystem I
photosystem II
cytochrome b/f complex
ATP synthase
RubisCO large subunit
RNA polymerase
ribosomal proteins (SSU)
ribosomal proteins (LSU)
transfer RNAs
ribosomal RNAs
clpP, matK
other genes
hypothetical chloroplast reading frames (ycf)

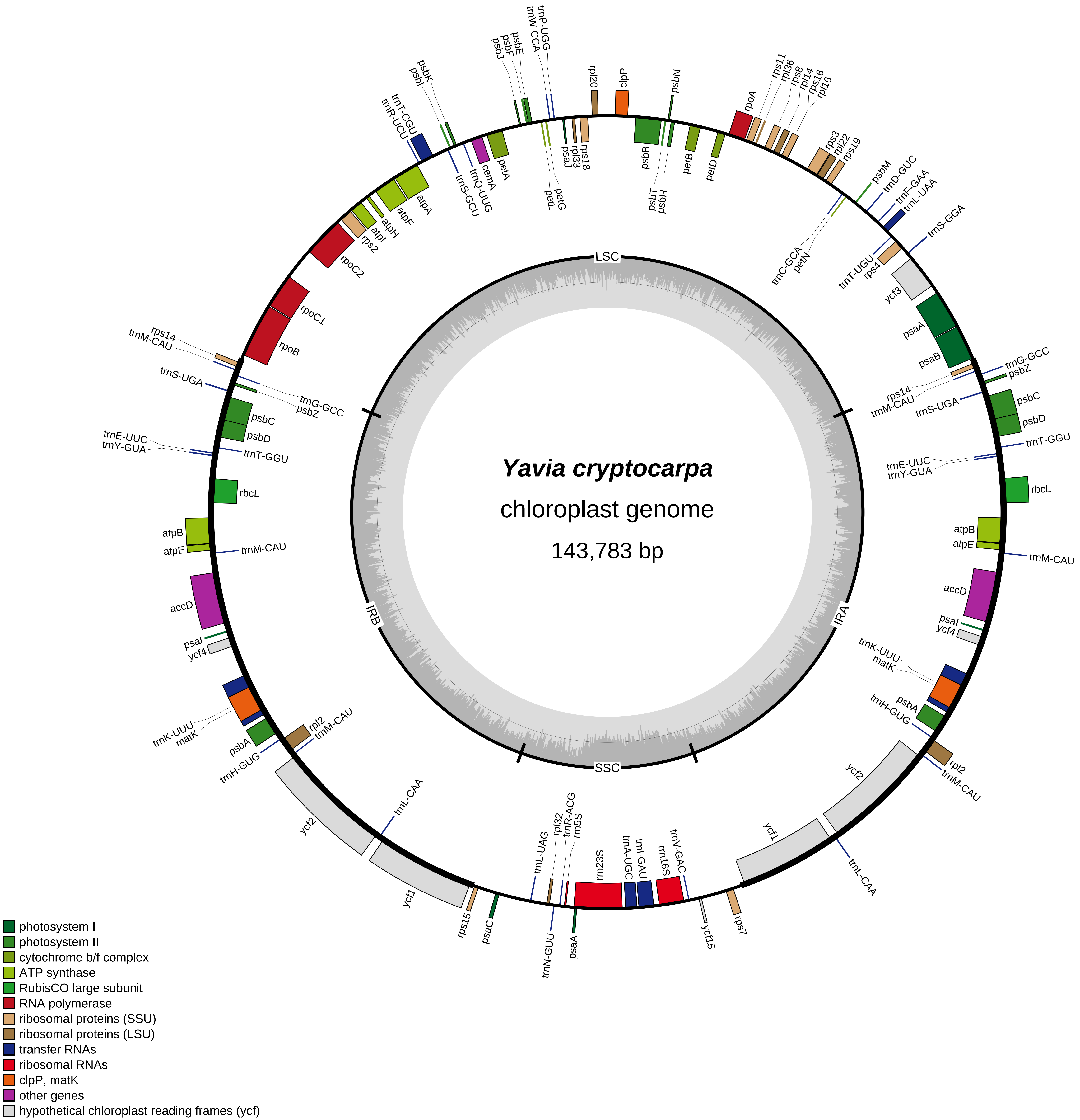

Yavia cryptocarpa
chloroplast genome
143,783 bp
LSC
IRA
SSC
IRB
rpoA
rps11
rpl36
rps8
rpl14
rps16
rpl16
rps3
rpl22
rps19
psbM
trnD-GUC
trnF-GAA
trnL-UAA
trnS-GGA
trnC-GCA
petN
trnT-UGU
rps4
ycf3
psaA
psaB
trnG-GCC
psbZ
rps14
trnM-CAU
trnS-UGA
psbC
psbD
trnT-GGU
trnE-UUC
trnY-GUA
rbcL
atpB
atpE
trnM-CAU
accD
psaI
ycf4
trnK-UUU
matK
psbA
trnH-GUG
rpl2
trnM-CAU
ycf2
ycf1
trnL-CAA
rps7
ycf15
trnV-GAC
rrn16S
trnI-GAU
trnA-UGC
rrn23S
rrn5S
trnR-ACG
rpl32
trnL-UAG
trnN-GUU
psaA
psaC
rps15
ycf1
trnL-CAA
ycf2
trnM-CAU
rpl2
psbA
trnH-GUG
matK
trnK-UUU
ycf4
psaI
accD
atpE
trnM-CAU
atpB
rbcL
trnE-UUC
trnY-GUA
trnT-GGU
psbD
psbC
trnS-UGA
psbZ
trnG-GCC
trnM-CAU
rps14
rpoB
rpoC1
rpoC2
rps2
atpI
atpH
atpF
atpA
trnR-UCU
trnT-CGU
psbI
psbK
trnS-GCU
trnQ-UUG
cemA
petA
psbJ
psbF
psbE
trnW-CCA
trnP-UGG
petL
petG
psaJ
rpl33
rps18
rpl20
clpP
psbN
psbB
psbT
psbH
petB
petD
photosystem I
photosystem II
cytochrome b/f complex
ATP synthase
RubisCO large subunit
RNA polymerase
ribosomal proteins (SSU)
ribosomal proteins (LSU)
transfer RNAs
ribosomal RNAs
clpP, matK
other genes
hypothetical chloroplast reading frames (ycf)
