# Supplementary material for: Plastome variations reveal the distinct evolutionary scenarios of plastomes in the subfamily Cereoideae (Cactaceae)
Source: BMC Plant Biol. 2023 Mar 8;23:132. doi: 10.1186/s12870-023-04148-4 (PMC9993602; doi:10.1186/s12870-023-04148-4)
Supplement: Supplementary file 2 — Supplementary Material 2 [file 12870_2023_4148_MOESM2_ESM.pdf]

Figure S2 A ~60-kb inversion observed in LSC region in Cactaceae plastomes. a. The linear maps of plastomes. Take the comparison of *Astrophytum myriostigma* and *Ariocarpus retusus* as an example, the dotted lines in orange indicate inversions, the blue lines indicate the region of changes in genome position (not inverted). **b.** The dot-plots of *As. myriostigma with itself and Ar. retusus*. A pair of short-inverted repeats (SIR) was found at the place of the ~60-kb inversion in *As. myriostigma* **c.** The remaining 19 plastomes with this SIR structure. The red circle marked the SIR structure, and the blue circle marked some of the other repeats. It is worth noting that in genus *Selenicereus*, due to the expansion of IR regions, SIR structures were captured by IR region and its position has changed. This SIR structure perhaps mediated intramolecular recombination. Table S7 listed the positions and lengths of this SIR structures.

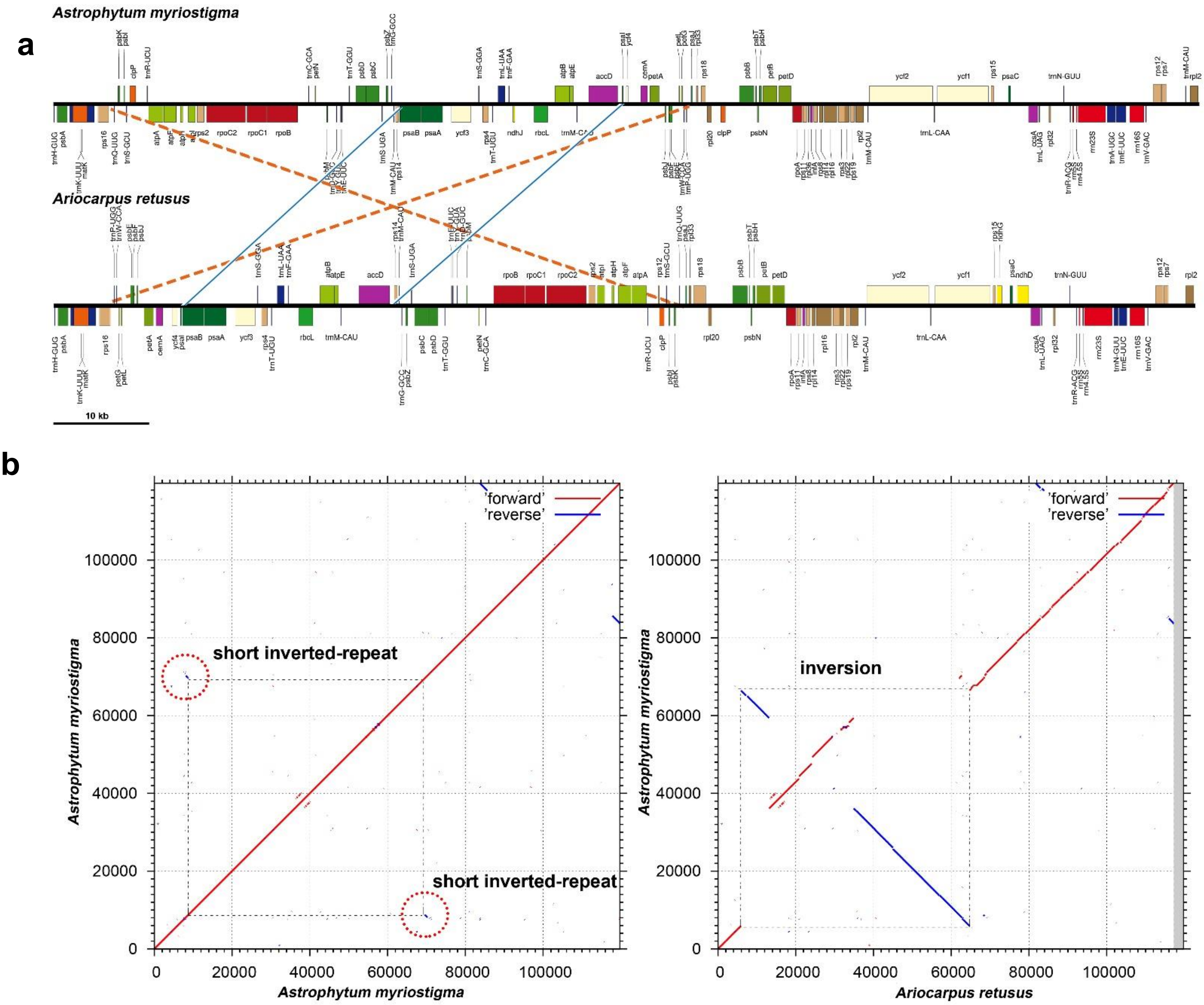

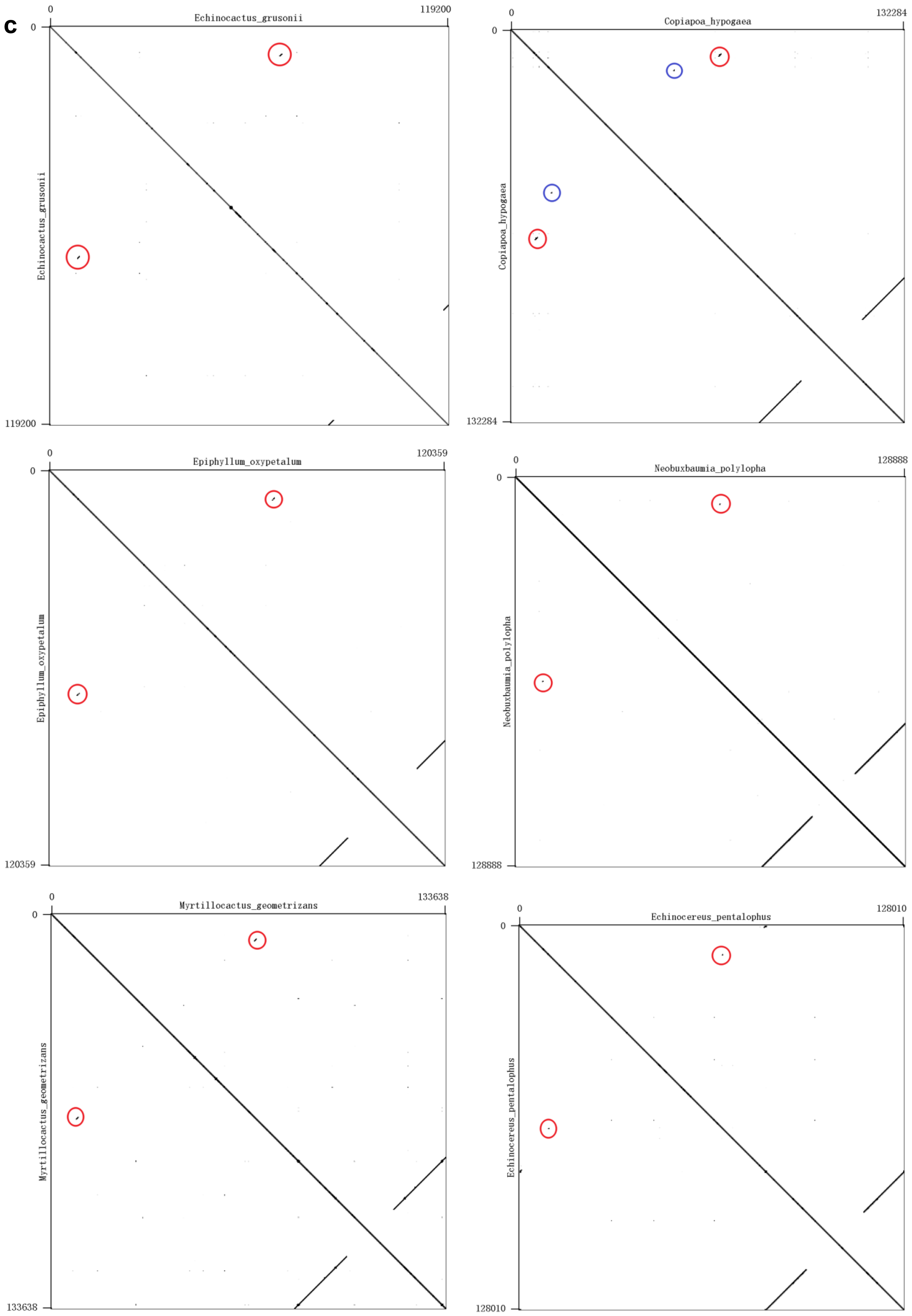
c
0
Echinocactus_grusonii
119200
0
Echinocactus_grusonii
119200
0
Copiapoa_hypogaea
132284
0
Copiapoa_hypogaea
132284
0
Epiphyllum_oxypetalum
120359
0
Epiphyllum_oxypetalum
120359
0
Neobuxbaumia_polylopha
128888
0
Neobuxbaumia_polylopha
128888
0
Myrtillocactus_geometrizans
133638
0
Myrtillocactus_geometrizans
133638
0
Echinocereus_pentalophus
128010
0
Echinocereus_pentalophus
128010

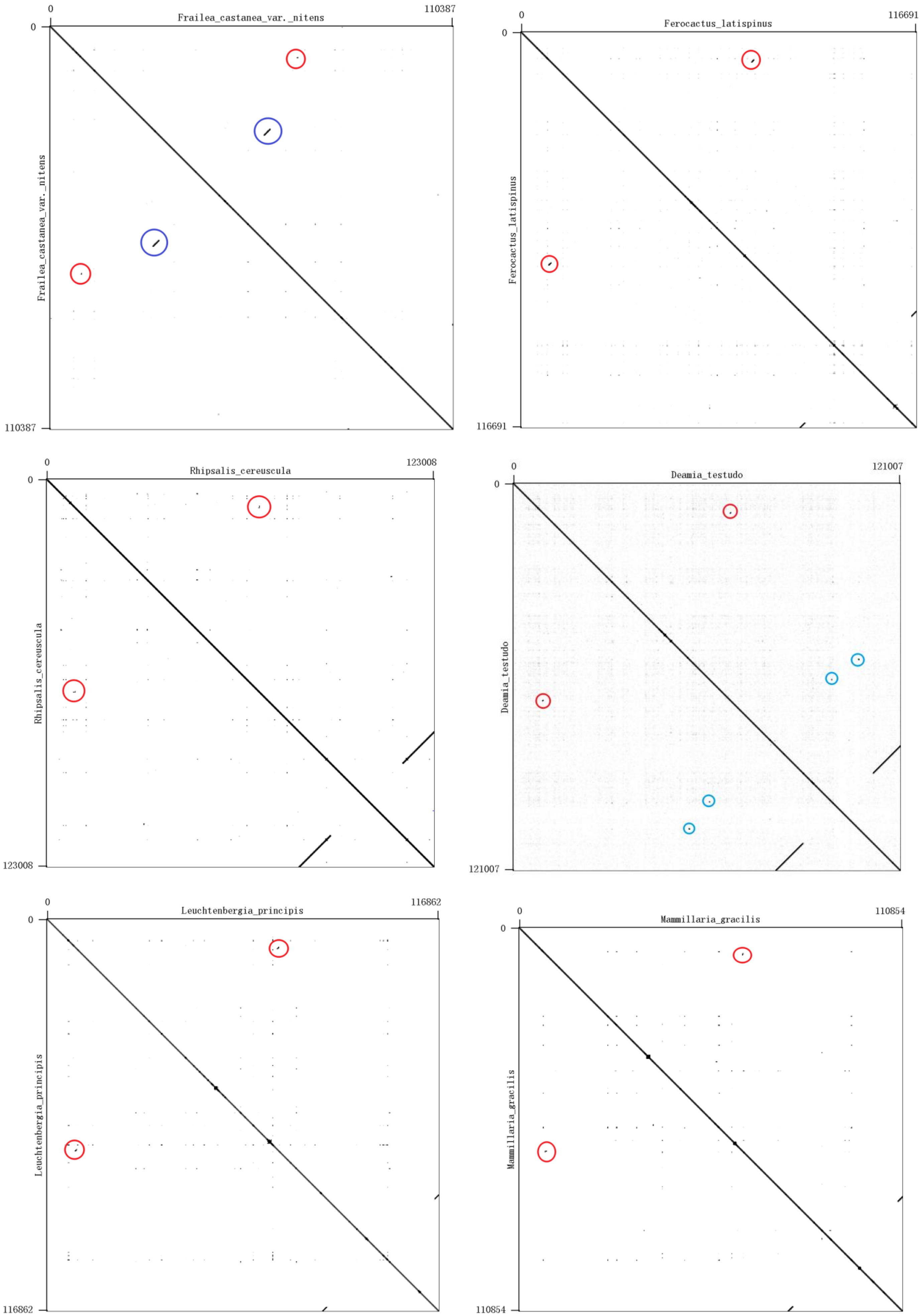

0
Frailea_castanea_var._nitens
110387
0
Frailea_castanea_var._nitens
110387
0
Ferocactus_latispinus
116691
0
Ferocactus_latispinus
116691
0
Rhipsalis_cereuscula
123008
0
Rhipsalis_cereuscula
123008
0
Deamia_testudo
121007
0
Deamia_testudo
121007
0
Leuchtenbergia_principis
116862
0
Leuchtenbergia_principis
116862
0
Mammillaria_gracilis
110854
0
Mammillaria_gracilis
110854

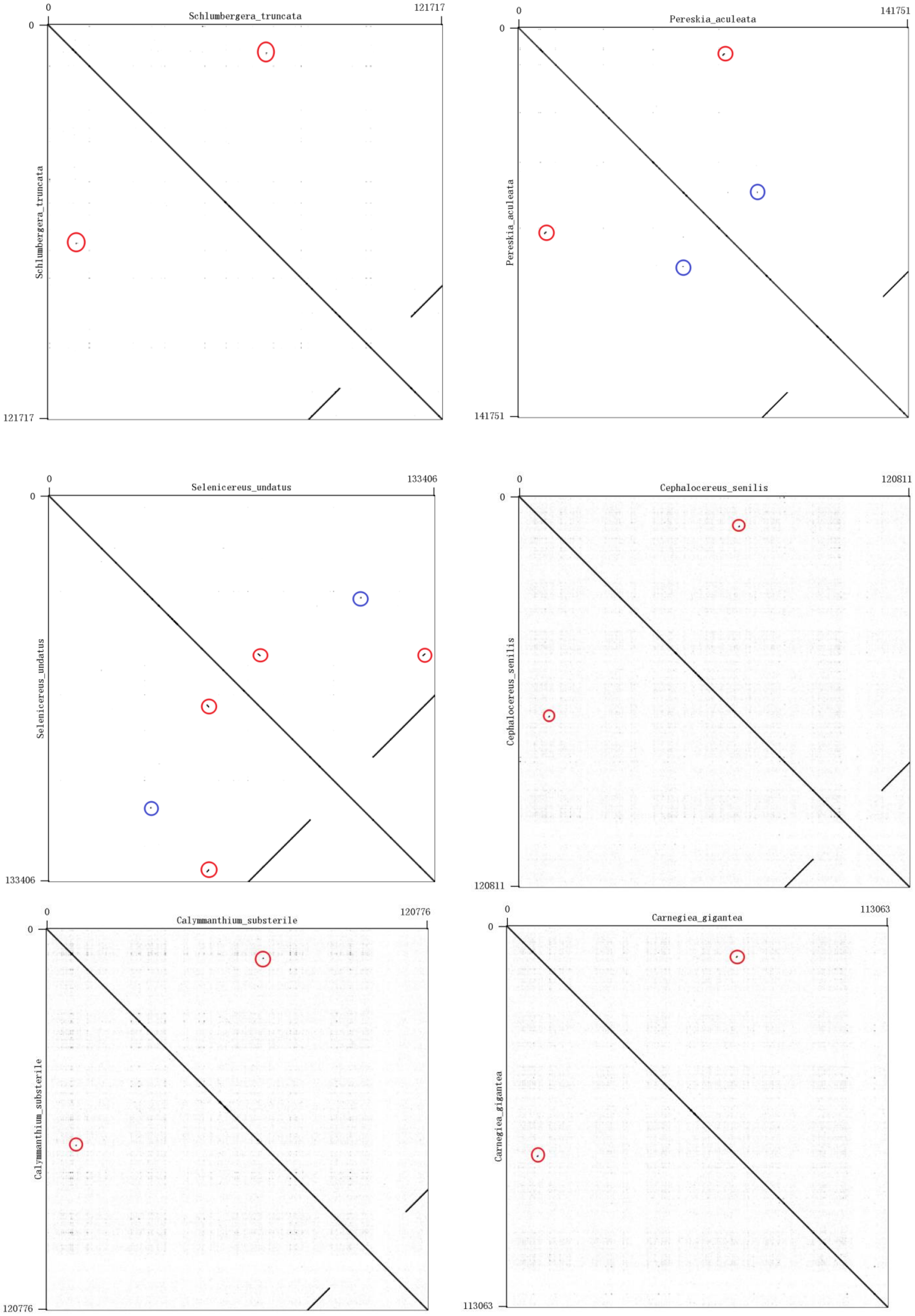
0
Schlumbergera_truncata
121717
0
Schlumbergera_truncata
121717
0
Pereskia_aculeata
141751
0
Pereskia_aculeata
141751
0
Selenicereus_undatus
133406
0
Selenicereus_undatus
133406
0
Cephalocereus_senilis
120811
0
Cephalocereus_senilis
120811
0
Calymmanthium_substerile
120776
0
Calymmanthium_substerile
120776
0
Carnegiea_gigantea
113063
0
Carnegiea_gigantea
113063

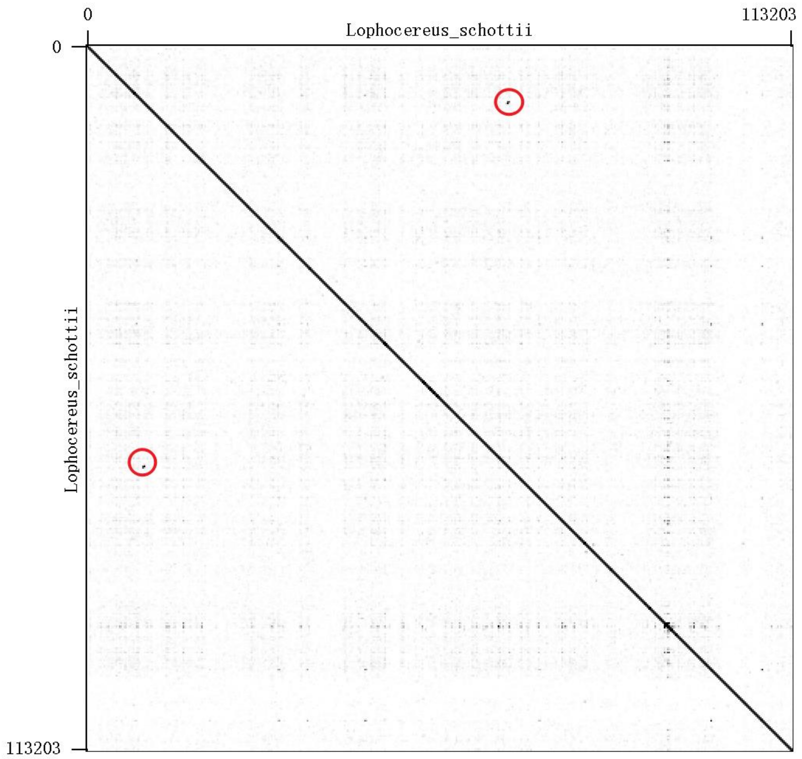

0
Lophocereus_schottii
113203
0
Lophocereus_schottii
113203

Figure S3 Dot-plots among *Portulaca oleracea* and *Pereskia aculeate*, *Opuntia microdasys*. The SSC region of *Pe. aculeate* and *Op. microdasys* shared the same rearrangement event. Mark the SSC region with a gray dotted box. A small rearrangement (6-kb) was observed in the LSC region of *Pe. aculeate*, and a large rearrangement (60-kb) was also observed in the LSC region of *Op. microdasys*.

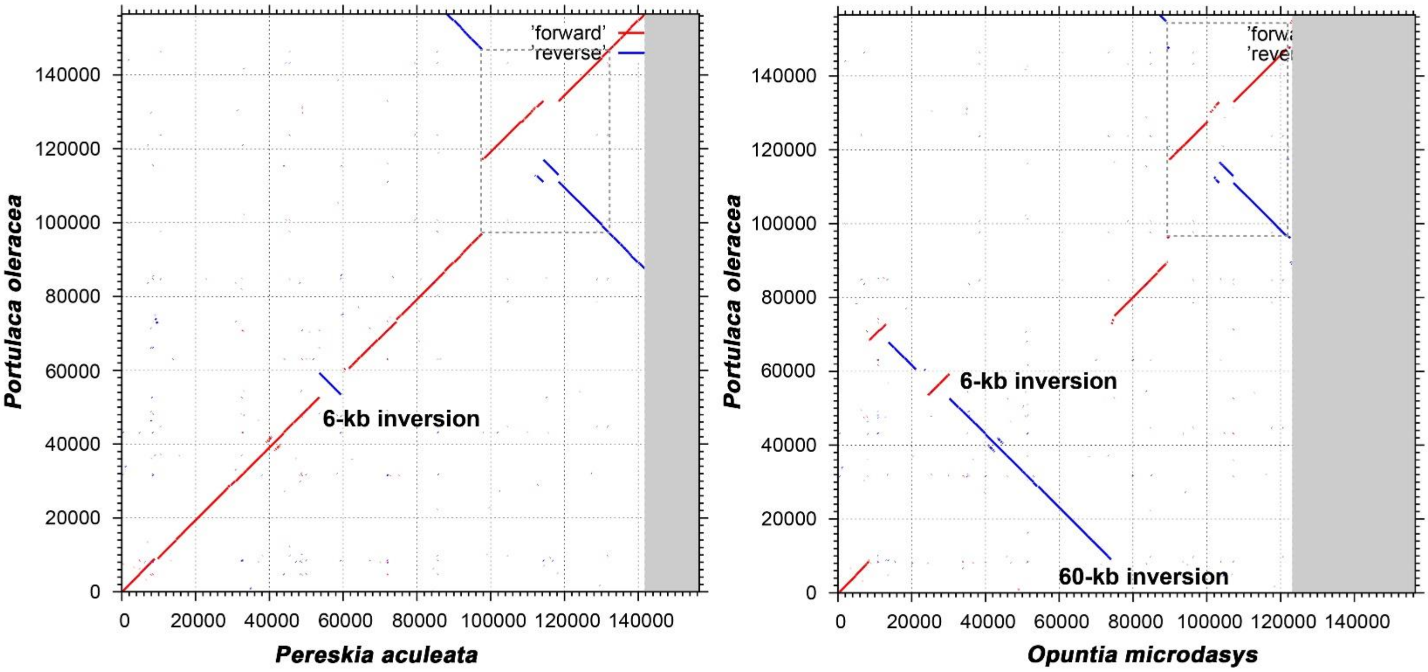

Figure S4 Dot-plots among Cacteae plastomes. We only exhibited the plastomes that underwent genome rearrangement events.

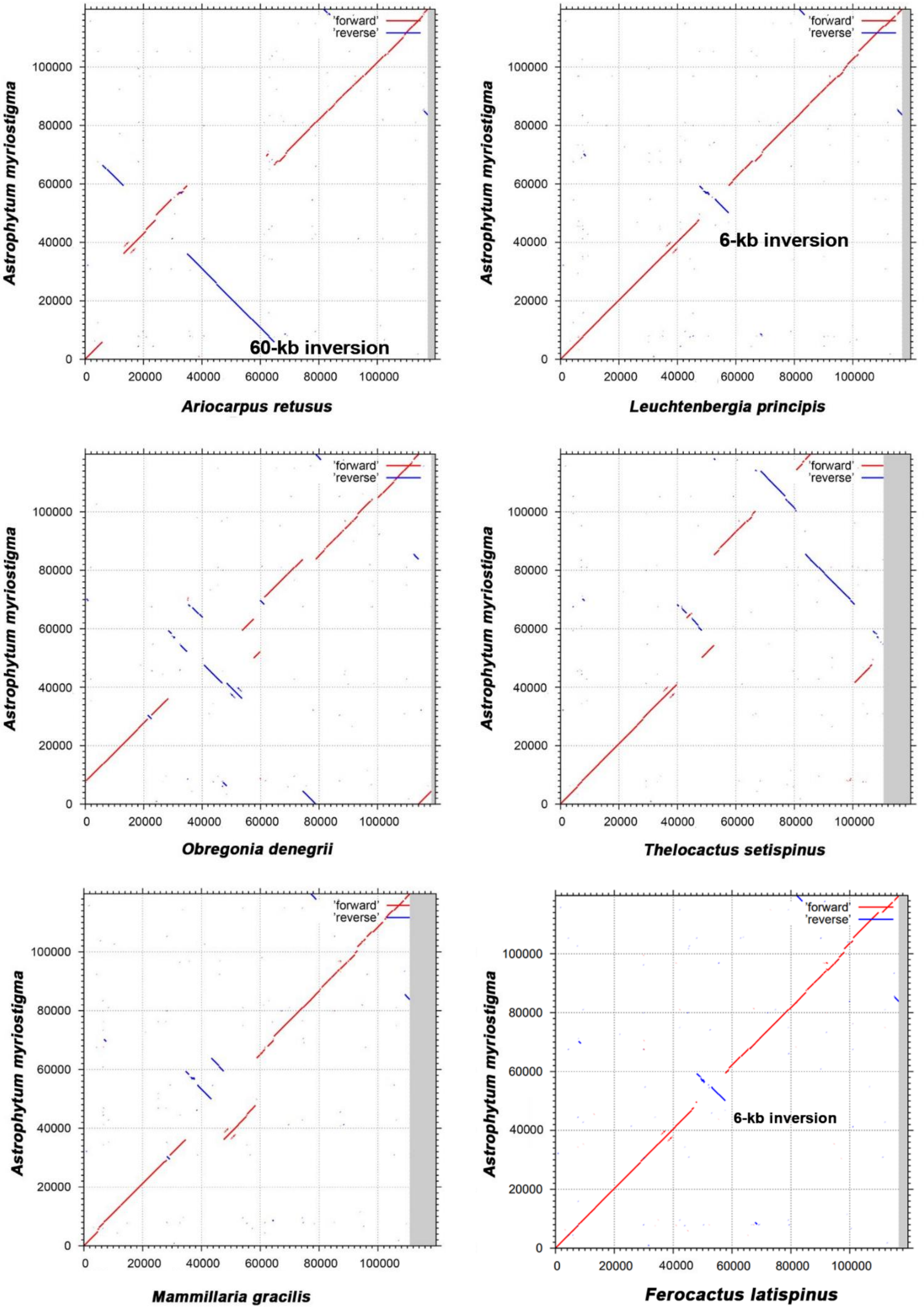

Figure S5 Dot-plots among Cacteae (*Astrophytum myriostigma*), *Copiapoa hypogaea*, *Frailea castanea var. nitens* and two Rhipsalideae plastomes (*Rhipsalis cereuscula* and *Schlumbergera truncata*). An 18 kb inversion was observed in the LSC region of *Co. hypogaea* and two Rhipsalideae species compared to the *As. myriostigma*, and the later had also observed another small-scale inversion of ~2.5 kb. Another rearrangement was observed in *Fr. castanea var. nitens*. The linear plastome maps showed the the genes associated with the above mentioned inversion and rearrangement.

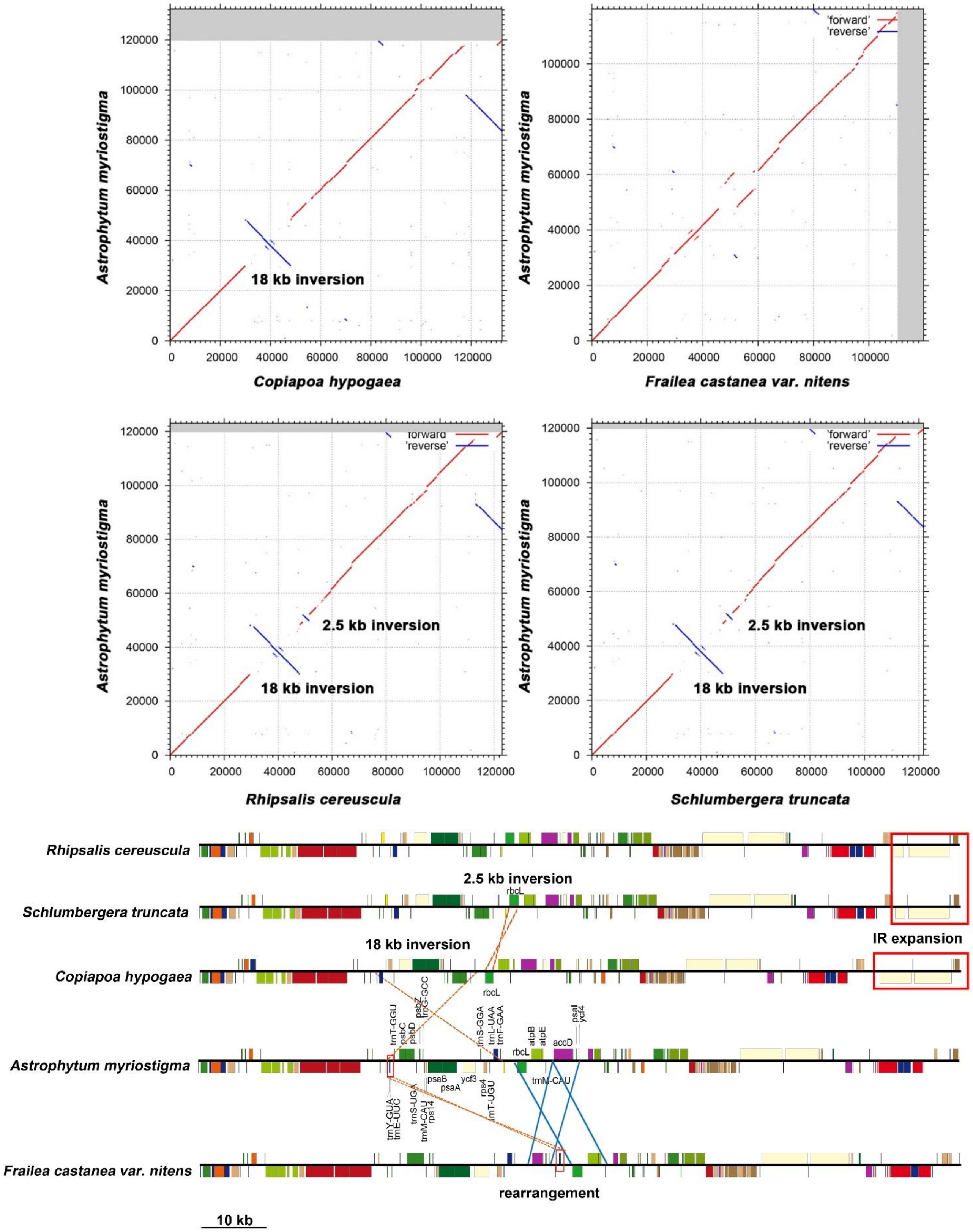

Figure S6 Another rearrangement event occurred in SSC region. a. The Dot-plots among *Pereskia aculeate* and other cacti plastomes (take *Astrophytum myriostigma* as an example). The SSC region of most Cereoideae plastomes shared a small inversion and rearrangement, the deletion of multiple fragments in SSC possibly resulted in the loss of *ndh* gene suite. **b.** The linear plastome maps. The solid lines in blue indicate rearrangement, and dotted lines in orange indicate inversion.

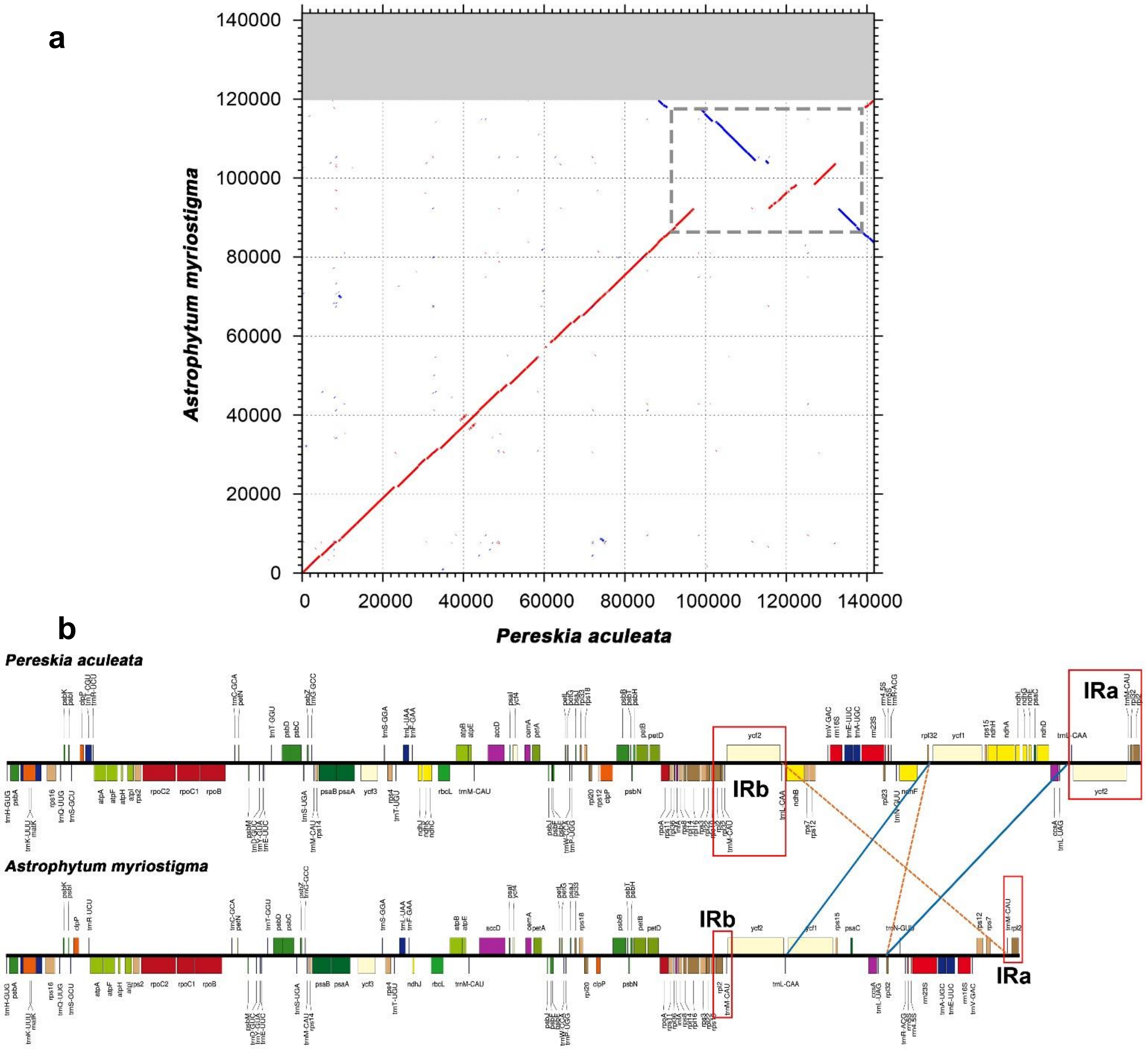

Figure S7 Sanger sequencing results. There were four different Sanger reads: the Sanger reads of F1+R1 and F2+R2 supports isomer 1, however, F1+R2 and F2 +R1 supports genome recombination (that is the isomer 2). The length of PCR products was 819 bp, 785 bp, 804 bp and 800 bp, respectively, which was slightly shorter than the estimated product lengths. Panels **a**, **b**, **c** and **d** are the alignment of the corresponding genomic regions with the PCR products. The genomic position is marked at the top of the base.

a

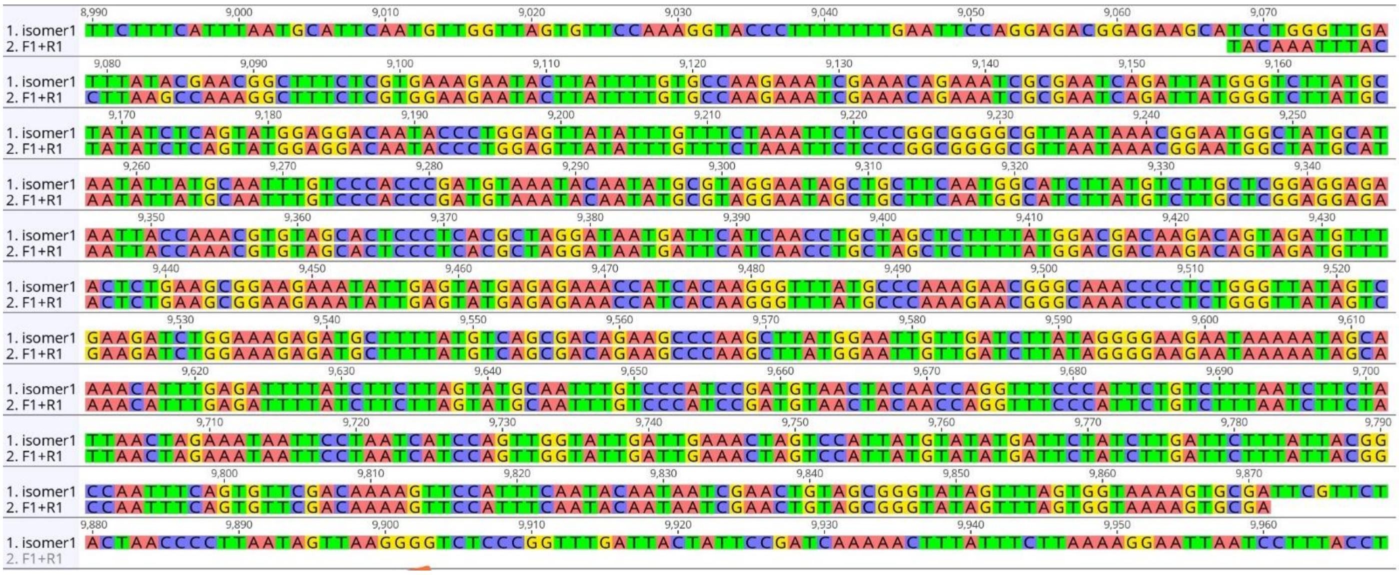

b

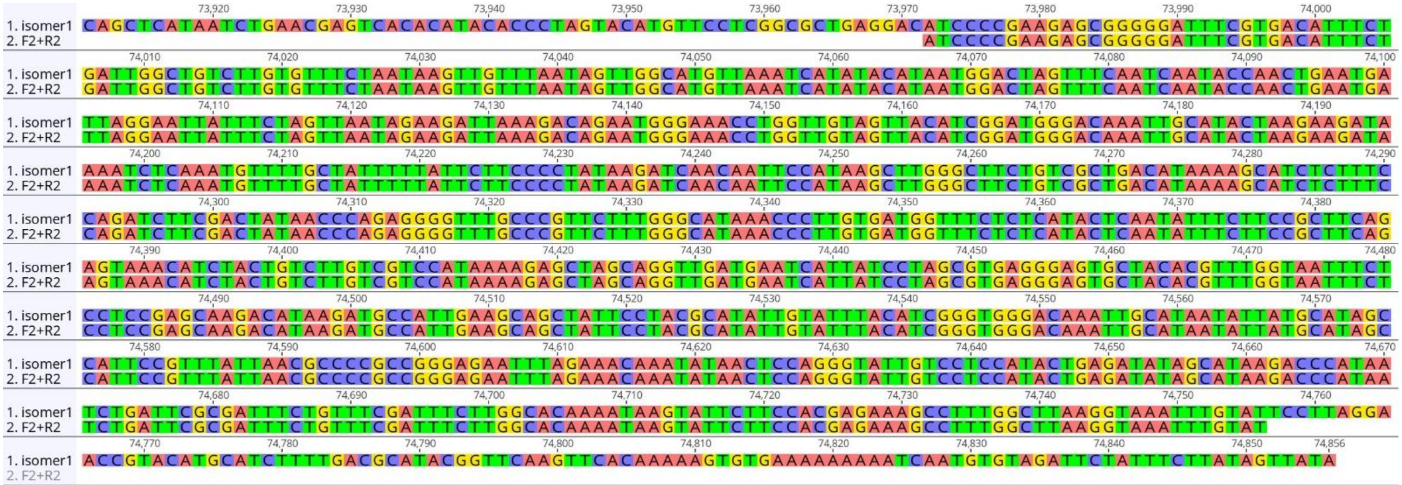

c

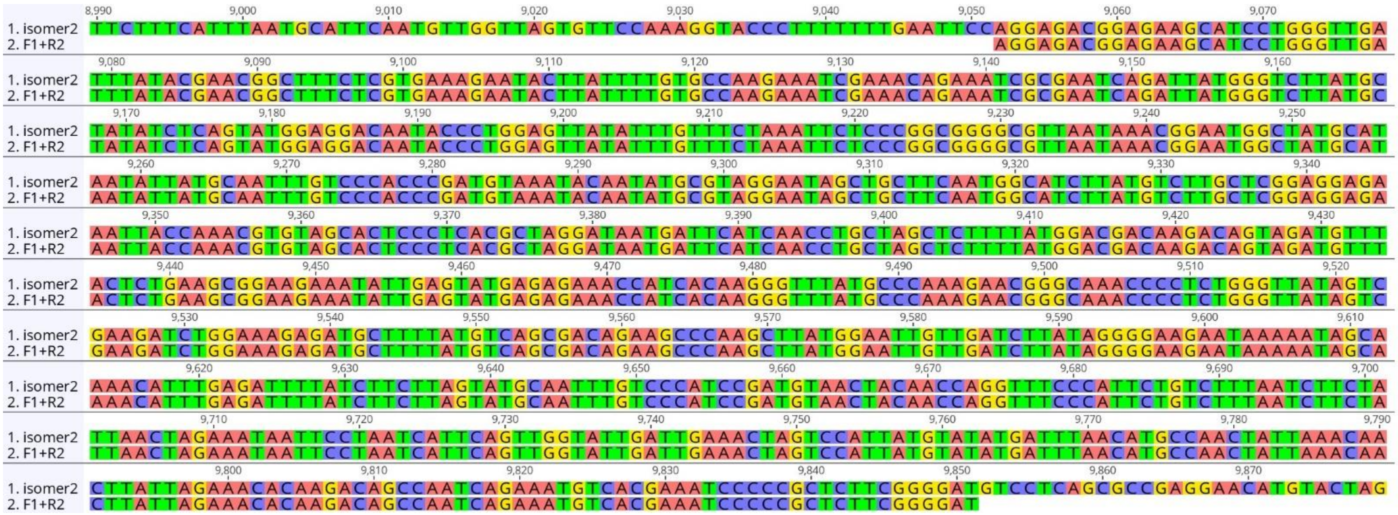

d

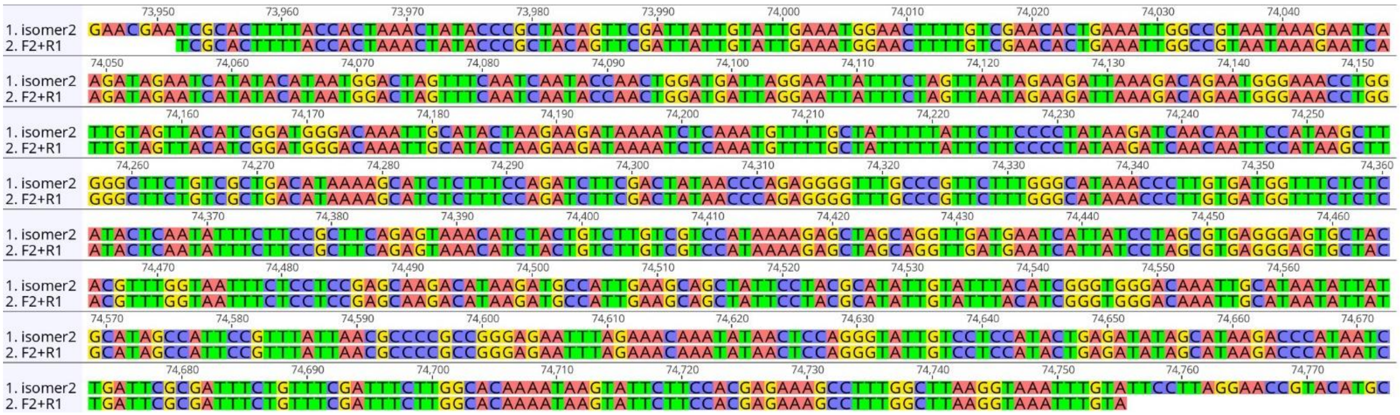
1. isomer2
2. F2+R1
