# Supplementary material for: Plastome variations reveal the distinct evolutionary scenarios of plastomes in the subfamily Cereoideae (Cactaceae)
Source: BMC Plant Biol. 2023 Mar 8;23:132. doi: 10.1186/s12870-023-04148-4 (PMC9993602; doi:10.1186/s12870-023-04148-4)
Supplement: Supplementary file 4 — Supplementary Material 4 [file 12870_2023_4148_MOESM4_ESM.docx]

**Table S4 list of the plastomes with inverted repeats which are probably associated with the ~ 60-kb inversion**

| Species | Repeat length (bp) | R1 start | R1 end | R2 start | R2 end |  |
| --- | --- | --- | --- | --- | --- | --- |
| *Schlumbergera truncata* | 67 | 8,934 | 9,000 | 66,952 | 67,018 |  |
| *Rhipsalis cereuscula* | 54 | 8,780 | 8,833 | 67,202 | 67,149 |  |
| *Frailea castanea var. nitens* | 80 | 8,343 | 8,422 | 67,407 | 67,486 |  |
| *Selenicereus undatus* | 293 | 54,574 | 54,866 | 129,282 | 129,574 | IR expansion |
| *Epiphyllum oxypetalum* | 175 | 8,799 | 8,973 | 67,445 | 67,619 |  |
| *Neobuxbaumia polylopha* | 143 | 8,738 | 8,880 | 67,165 | 67,307 |  |
| *Myrtillocactus geometrizans* | 192 | 8,652 | 8,843 | 68,406 | 68,597 |  |
| *Echinocereus pentalophus* | 213 | 9,427 | 9,639 | 67,179 | 67,391 |  |
| *Copiapoa hypogaea* | 464 | 8,205 | 8,668 | 69,569 | 70,032 |  |
| *Echinocactus grusonii* | 164 | 8,474 | 8,637 | 68,446 | 68,609 |  |
| *Leuchtenbergia principis* | 146 | 8,490 | 8,635 | 68,406 | 68,551 |  |
| *Ferocactus latispinus* | 148 | 8,457 | 8,604 | 67,722 | 67,869 |  |
| *Mammillaria gracilis* | 149 | 7,440 | 7,588 | 64,186 | 64,334 |  |
| *Astrophytum myriostigma* | 146 | 8,607 | 8,752 | 69,541 | 69,686 |  |
| *Pereskia aculeata* | 679 | 9,103 | 9,781 | 74,097 | 74,775 |  |
| *Deamia testudo* | 129 | 8,890 | 9,020 | 67,483 | 67,613 |  |
| *Cephalocereus senilis* | 175 | 8,975 | 9,151 | 67,602 | 67,778 |  |
| *Calymmanthium substerile* | 198 | 8,963 | 9,162 | 68,203 | 68,402 |  |
| *Lophocereus schottii* | 199 | 8,834 | 9,034 | 67,135 | 67,335 |  |
| *Carnegiea gigantea* | 175 | 8,858 | 9,034 | 67,769 | 67,945 |  |

**Table S5 Summary of assembled plastomes in this study**

| Species | Tribe | Subfamily | NCBI Accession No. | Voucher | Collecting locations/Source |
| --- | --- | --- | --- | --- | --- |
| *Copiapoa hypogaea* F.Ritter | / | Cactoideae | MW553047 | Yujie-001 | Beibei, Chongqing |
| *Epiphyllum oxypetalum* (DC.) Haw. | Hylocereeae | Cactoideae | MW553050 | Yujie-003 | Yulin, Guangxi |
| *Neobuxbaumia polylopha* (DC.) Backeb. | Hylocereeae | Cactoideae | MW553061 | Yujie-004 | Greenhouse of Southwest University, Chongqing |
| *Myrtillocactus geometrizans* (Mart. ex Pfeiff.) Console | Hylocereeae | Cactoideae | MW553060 | Yujie-005 | Greenhouse of Southwest University, Chongqing |
| *Echinocereus pentalophus (DC.) Lem.* | Hylocereeae | Cactoideae | MW553049 | Yujie-006 | Beibei, Chongqing |
| *Frailea castanea var. nitens* | / | Cactoideae | MW553053 | Yujie-008 | Beibei, Chongqing |
| *Obregonia denegrii* Frič | Cacteae | Cactoideae | MW553062 | Yujie-009 | Zhangzhou, Fujian |
| *Echinocactus grusonii* Hildm. | Cacteae | Cactoideae | MW553048 | Yujie-010 | Beibei, Chongqing |
| *Ferocactus latispinus* (Karw. ex Pfeiff.) N.P.Taylor | Cacteae | Cactoideae | MW553072 | Yujie-011 | Beibei, Chongqing |
| *Thelocactus setispinus* (Engelm.) E.F. Anderson | Cacteae | Cactoideae | MW553071 | Yujie-013 | Beibei, Chongqing |
| *Astrophytum myriostigma* Lem. | Cacteae | Cactoideae | MW553044 | Yujie-014 | Beibei, Chongqing |
| *Rhipsalis cereuscula* Haw. | Rhipsalideae | Cactoideae | MW553066 | Yujie-015 | Beibei, Chongqing |
| *Parodia scopa*(Spreng.) N.P. Taylor | Notocacteae | Cactoideae | MW553045 | Yujie-016 | Zhangzhou, Fujian |
| *Cleistocactus winteri D.R.Hunt* | Cereeae | Cactoideae | MW553046 | Yujie-018 | Greenhouse of Southwest University, Chongqing |
| *Espostoa lanata* (Kunth) Britton & Rose | Cereeae | Cactoideae | MW553052 | Yujie-019 | Greenhouse of Southwest University, Chongqing |
| *Pilosocereus pachycladus* F. Ritter | Cereeae | Cactoideae | MW553065 | Yujie-020 | Greenhouse of Southwest University, Chongqing |
| *Acanthocereus tetragonus (L.) Hummelinck* | Cereeae | Cactoideae | MW553073 | Yujie-021 | Beibei, Chongqing |
| *Gymnocalycium saglionis* (Cels) Britton & Rose | Cereeae | Cactoideae | MW553054 | Yujie-022 | Greenhouse of Southwest University, Chongqing |
| *Ariocarpus retusus*Scheidw. | Cacteae | Cactoideae | MW553043 | Yujie-023 | Beibei, Chongqing |
| *Selenicereus undatus* (Haw.) Britton & Rose | Hylocereeae | Cactoideae | MW553056 | Yujie-024 | Yulin, Guangxi |
| *Leuchtenbergia principis* Hook. | Cacteae | Cactoideae | MW553057 | Yujie-025 | Beibei, Chongqing |
| *Mammillaria gracilis* Pfeiff. | Cacteae | Cactoideae | MW553059 | Yujie-026 | Beibei, Chongqing |
| *Matucana haynei* (Otto ex Salm-Dyck) Britton & Rose | Cereeae | Cactoideae | MW553051 | Yujie-030 | Greenhouse of Southwest University, Chongqing |
| *Schlumbergera truncata* (Haw.) Moran | Rhipsalideae | Cactoideae | MW553067 | Yujie-031 | Beibei, Chongqing |
| *Opuntia microdasys* (Lehm.) Pfeiff. | Opuntieae | Opuntioideae | MW553063 | Yujie-032 | Beibei, Chongqing |
| *Pereskia aculeata* Mill. | Pereskieae | Pereskioideae | MW553064 | Yujie-033 | Beibei, Chongqing |
| *Carnegiea gigantea* (Engelm.) Britton & Rose | Hylocereeae | Cactoideae | NC_027618.1 | / | NCBI |
| *Lophocereus schottii* (Engelm.) Britton & Rose | Hylocereeae | Cactoideae | NC_041727.1 | / | NCBI |
| *Deamia testudo* (Karw. ex Zucc.) Britton & Rose | Hylocereeae | Cactoideae | / | / | Kew Tree of Life Project |
| *Cephalocereus senilis* (Haw.) Pfeiff. | Hylocereeae | Cactoideae | / | / | Kew Tree of Life Project |
| *Yavia cryptocarpa* R. Kiesling & Piltz | Notocacteae | Cactoideae | / | / | Kew Tree of Life Project |
| *Praecereus euchlorus* (F.A.C.Weber ex K.Schum.) N.P.Taylor | Cereeae | Cactoideae | / | / | Kew Tree of Life Project |
| *Rebutia pygmaea* (R.E. Fr.) Britton & Rose | Cereeae | Cactoideae | / | / | Kew Tree of Life Project |
| *Coleocephalocereus fluminensis* (Miq.) Backeb | Cereeae | Cactoideae | / | / | Kew Tree of Life Project |
| *Calymmanthium substerile* F.Ritter | / | Cactoideae | / | / | Kew Tree of Life Project |
| *Cereus jamacaru* DC | Cereeae | Cactoideae | / | / | Kew Tree of Life Project |
| *Echinopsis mirabilis* Speg | Cereeae | Cactoideae | / | / | Kew Tree of Life Project |

**Table S6 Summary of sequencing data quality**

| Species | Raw Reads | Clean Reads | Raw Base (G) | Clean Base (G) | Effective Rate (%) | Error Rate (%) | Q20 (%) | Q30 (%) | GC Content (%) |
| --- | --- | --- | --- | --- | --- | --- | --- | --- | --- |
| *Ariocarpus retusus* | 19,125,320 | 18,892,671 | 5.74 | 5.67 | 98.78 | 0.03 | 97.12 | 91.85 | 39.95 |
| *Astrophytum myriostigma* | 21,639,180 | 21,515,261 | 6.49 | 6.45 | 99.43 | 0.03 | 98.12 | 94.38 | 42.56 |
| *Acanthocereus tetragonus* | 19,058,912 | 18,820,876 | 5.72 | 5.65 | 98.75 | 0.03 | 97.27 | 92.25 | 42.56 |
| *Cleistocactus winteri* | 19,813,243 | 19,695,440 | 5.94 | 5.91 | 99.41 | 0.03 | 97.41 | 92.59 | 49.27 |
| *Copiapoa hypogaea* | 18,880,758 | 18,749,506 | 5.66 | 5.62 | 99.3 | 0.03 | 97.69 | 93.47 | 49.84 |
| *Echinocactus grusonii* | 18,918,925 | 18,854,306 | 5.68 | 5.66 | 99.66 | 0.03 | 98.01 | 94.15 | 46.96 |
| *Echinocereus pentalophus* | 19,133,261 | 18,964,937 | 5.74 | 5.69 | 99.12 | 0.03 | 97.31 | 92.48 | 38.45 |
| *Epiphyllum oxypetalum* | 19,450,243 | 19,049,612 | 5.84 | 5.71 | 97.94 | 0.03 | 96.78 | 91.39 | 40.19 |
| *Espostoa lanata* | 18,498,355 | 18,445,455 | 5.55 | 5.53 | 99.71 | 0.03 | 97.72 | 93.75 | 40.02 |
| *Ferocactus latispinus* | 19,710,565 | 19,595,414 | 5.91 | 5.88 | 99.42 | 0.03 | 97.68 | 93.44 | 49.62 |
| *Frailea castanea var. nitens* | 19,250,449 | 19,141,256 | 5.78 | 5.74 | 99.43 | 0.03 | 97.69 | 93.13 | 38.51 |
| *Gymnocalycium saglionis* | 19,048,792 | 18,844,156 | 5.71 | 5.65 | 98.93 | 0.03 | 97.34 | 92.4 | 41.94 |
| *Selenicereus undatus* | 19,069,755 | 18,811,901 | 5.72 | 5.64 | 98.65 | 0.03 | 96.78 | 91.34 | 39.04 |
| *Leuchtenbergia principis* | 19,049,592 | 18,801,724 | 5.71 | 5.64 | 98.7 | 0.03 | 97.34 | 92.49 | 41.13 |
| *Mammillaria gracilis* | 18,366,185 | 18,207,572 | 5.51 | 5.46 | 99.14 | 0.03 | 97.53 | 92.94 | 38.97 |
| *Matucana haynei* | 23,338,407 | 23,128,304 | 7 | 6.94 | 99.1 | 0.03 | 97.03 | 91.83 | 40.17 |
| *Myrtillocactus geometrizans* | 19,526,661 | 19,337,703 | 5.86 | 5.8 | 99.03 | 0.03 | 97.48 | 92.86 | 39.45 |
| *Neobuxbaumia polylopha* | 20,657,132 | 20,622,167 | 6.2 | 6.19 | 99.83 | 0.03 | 97.87 | 94.07 | 39.87 |
| *Obregonia denegrii* | 18,976,265 | 18,886,686 | 5.69 | 5.67 | 99.53 | 0.03 | 97.64 | 93.5 | 44.35 |
| *Opuntia microdasys* | 21,374,905 | 21,171,315 | 6.41 | 6.35 | 99.05 | 0.03 | 97.3 | 92.67 | 38.3 |
| *Parodia scopa* | 21,042,293 | 21,001,006 | 6.31 | 6.3 | 99.8 | 0.03 | 97.92 | 94.07 | 47.44 |
| *Pereskia aculeata* | 17,835,750 | 17,631,944 | 5.35 | 5.29 | 98.86 | 0.03 | 96.97 | 91.75 | 37.84 |
| *Pilosocereus pachycladus* | 19,060,478 | 18,856,073 | 5.72 | 5.66 | 98.93 | 0.03 | 97.08 | 91.75 | 39.29 |
| *Rhipsalis cereuscula* | 19,360,705 | 19,140,612 | 5.81 | 5.74 | 98.86 | 0.03 | 97.34 | 92.5 | 40.05 |
| *Selenicereus validus* | 22,696,845 | 22,482,739 | 6.81 | 6.74 | 99.06 | 0.03 | 97.25 | 92.31 | 39.09 |
| *Thelocactus setispinus* | 21,552,826 | 21,183,769 | 6.47 | 6.36 | 98.29 | 0.03 | 96.91 | 91.94 | 41.12 |

**Table S7 Assembly results of the Cactaceae plastomes**

| Species | GetOrganelle v1.7.3 | | | | NOVOPlasty v3.8.1 | | | |
| --- | --- | --- | --- | --- | --- | --- | --- | --- |
|  | Reads used for assembly | Kmer-coverage | Base-coverage | Assembly results | Totol contigs | Largest contig | Smallest contig | Merged contigs results |
| *Ariocarpus retusus* | 10,101,559 | 203.3 | 662.8 | circular genome |  |  |  |  |
| *Astrophytum myriostigma* | 19,388,501 | 154.0 | 641.8 | 4 scaffold(s) | 3 | 62,845 bp | 21,757 bp | circular genome |
| *Acanthocereus tetragonus* | 9,339,826 | 182.2 | 594.2 | circular genome |  |  |  |  |
| *Cleistocactus winteri* | 8,924,455 | 175.0 | 570.7 | circular genome |  |  |  |  |
| *Copiapoa hypogaea* | 142,679 | 168.0 | 547.7 | circular genome |  |  |  |  |
| *Echinocactus grusonii* | 50,231 | 42.2 | 137.7 | 9 scaffold(s) | 6 | 104,247 bp | 316 bp | 1 linear contig |
| *Echinocereus pentalophus* | 111,688 | 165.4 | 539.3 | 18 scaffold(s) | 5 | 55,899 bp | 571 bp | circular genome |
| *Epiphyllum oxypetalum* | 21,683,600 | 192.0 | 626.2 | circular genome |  |  |  |  |
| *Espostoa lanata* | 78,695 | 73.5 | 239.6 | circular genome |  |  |  |  |
| *Ferocactus latispinus* | 128,319 | 182.9 | 596.5 | circular genome |  |  |  |  |
| *Frailea castanea var. nitens* | 3,623,002 | 157.5 | 656.1 | circular genome |  |  |  |  |
| *Gymnocalycium saglionis* | 108,969 | 209.7 | 684.0 | 5 scaffold(s) | 9 | 52,435 bp | 392 bp | circular genome |
| *Selenicereus undatus* | 9,977,200 | 184.2 | 600.7 | circular genome |  |  |  |  |
| *Leuchtenbergia principis* | 204,416 | 184.1 | 600.3 | 1 scaffold(s) | 11 | 50,534 bp | 3,336 bp | 1 linear contig |
| *Mammillaria gracilis* | 4,650,726 | 144.3 | 601.5 | circular genome |  |  |  |  |
| *Matucana haynei* | 1,584,827 | 162.6 | 530.3 | circular genome |  |  |  |  |
| *Myrtillocactus geometrizans* | 4,494,157 | 141.2 | 588.3 | circular genome |  |  |  |  |
| *Neobuxbaumia polylopha* | 28,590 | 391.5 | 1276.7 | 3 scaffold(s) | 11 | 30,943 bp | 4,345 bp | 1 linear contig |
| *Obregonia denegrii* | 114,920 | 132.0 | 430.5 | 3 scaffold(s) | 4 | 80,537 bp | 9,065 bp | circular genome |
| *Opuntia microdasys* | 3533970 | 182.5 | 595.0 | circular genome |  |  |  |  |
| *Parodia scopa* | 41,145 | 61.6 | 200.8 | 8 scaffold(s) | 23 | 65,075 bp | 273 bp | circular genome |
| *Pereskia aculeata* | 3,551,213 | 180.1 | 587.4 | circular genome |  |  |  |  |
| *Pilosocereus pachycladus* | 100,323 | 174.5 | 569.2 | 6 scaffold(s) | 23 | 52,409 bp | 197 bp | circular genome |
| *Rhipsalis cereuscula* | 9,560,375 | 182.6 | 595.5 | circular genome |  |  |  |  |
| *Schlumbergera truncata* | 6,717,205 | 185.7 | 605.5 | circular genome |  |  |  |  |
| *Thelocactus setispinus* | 10,922,459 | 153.7 | 640.5 | circular genome |  |  |  |  |
| *Deamia testudo* | 1,948,972 | 53.5 | 172.0 | circular genome |  |  |  |  |
| *Cephalocereus senilis* | 7,082,734 | 380.7 | 1178.17 | circular genome |  |  |  |  |
| *Yavia cryptocarpa* | 1,979,727 | 123.1 | 395.6 | circular genome |  |  |  |  |
| *Praecereus euchlorus* | 3,214,339 | 137.8 | 442.6 | circular genome |  |  |  |  |
| *Rebutia pygmaea* | 2,094,120 | 203.6 | 654.3 | circular genome |  |  |  |  |
| *Coleocephalocereus fluminensis* | 5,486,854 | 292.9 | 940.9 | circular genome |  |  |  |  |
| *Calymmanthium substerile* | 7,478,671 | 200.5 | 644.0 | circular genome |  |  |  |  |
| *Cereus jamacaru* | 3,623,243 | 186.9 | 600.3 | circular genome |  |  |  |  |
| *Echinopsis mirabilis* | 4,855,716 | 377.8 | 1213.7 | circular genome |  |  |  |  |

**Table S8 The plant materials source of an additional related-species (*Blossfeldia liliputana*) used in phylogenomic study**

| NCBI Accession number | Genes | Source | NCBI Accession number | Genes | Source |
| --- | --- | --- | --- | --- | --- |
| HQ620730.1 | *atpA* | NCBI | HQ621281.1 | psbK | NCBI |
| HQ620742.1 | *atpB* | NCBI | HQ621293.1 | psbL | NCBI |
| HQ620752.1 | *atpE* | NCBI | HQ621312.1 | psbN | NCBI |
| HQ620760.1 | *atpF* | NCBI | HQ621334.1 | rbcL | NCBI |
| HQ620771.1 | *atpH* | NCBI | HQ621353.1 | rpl14 | NCBI |
| HQ620782.1 | *atpI* | NCBI | HQ621364.1 | rpl16 | NCBI |
| HQ620792.1 | *ccsA* | NCBI | HQ621343.1 | rpl2 | NCBI |
| HQ620803.1 | *cemA* | NCBI | HQ621375.1 | rpl20 | NCBI |
| HQ620820.1 | *infA* | NCBI | HQ621386.1 | rpl22 | NCBI |
| HM041655.1 | *matK* | NCBI | HQ621436.1 | rpoA | NCBI |
| HQ620994.1 | *petA* | NCBI | HQ621447.1 | rpoB | NCBI |
| HQ621004.1 | *petB* | NCBI | DQ496218.1 | rpoC1 | NCBI |
| HQ621015.1 | *petD* | NCBI | HQ621467.1 | rpoC2 | NCBI |
| HQ621150.1 | *psaA* | NCBI | HQ621530.1 | rps11 | NCBI |
| HQ621160.1 | *psaB* | NCBI | HQ621541.1 | rps12 | NCBI |
| HQ621170.1 | *psaC* | NCBI | HQ621552.1 | rps14 | NCBI |
| HQ621190.1 | *psaJ* | NCBI | HQ621562.1 | rps15 | NCBI |
| HQ621199.1 | *psbA* | NCBI | HQ621593.1 | rps19 | NCBI |
| HQ621210.1 | *psbB* | NCBI | HQ621477.1 | rps2 | NCBI |
| HQ621220.1 | *psbC* | NCBI | HQ621487.1 | rps3 | NCBI |
| HQ621687.1 | *psbD* | NCBI | HQ621497.1 | rps4 | NCBI |
| HQ621230.1 | *psbE* | NCBI | HQ621508.1 | rps7 | NCBI |
| HQ621241.1 | *psbF* | NCBI | HQ621519.1 | rps8 | NCBI |
| HQ621251.1 | *psbH* | NCBI | HQ621666.1 | ycf3 | NCBI |
| HQ621271.1 | *psbJ* | NCBI |  |  |  |

*Note. Eight gene sequences (*petG*, *petL*, *petN*, *psaI*, *psbI*, *psbM*, *psbT* and *psbZ*) could not be retrieved in NCBI for *Blossfeldia liliputana*, and they were processed as missing data in the alignment.

**Table S9** Primers used in this study.

| Primer name | Primer sequence |
| --- | --- |
| F1 | 5'-TATACATGGCTCGACTCCCA-3' |
| R1 | 5'-GCATTCAATGTTGGTTAGTGTTCC-3' |
| F2 | 5'-TGCCCTATCACTAGTGGTTACT-3' |
| R2 | 5'-CTTTTTGTGAACTTGAACCGTATG-3' |
