# Supplementary material for: Plastome variations reveal the distinct evolutionary scenarios of plastomes in the subfamily Cereoideae (Cactaceae)
Source: BMC Plant Biol. 2023 Mar 8;23:132. doi: 10.1186/s12870-023-04148-4 (PMC9993602; doi:10.1186/s12870-023-04148-4)
Supplement: Supplementary file 5 — Supplementary Material 5 [file 12870_2023_4148_MOESM5_ESM.docx]

**Supplementary Tables:**

**Table S1.** The number of repeat elements in 35 cacti plastomes.

**Table S2.** Repeated units that identified in the 35 cacti plastomes by using ROUSFinder.

**Table S3**. Statistics of the location of repeated sequences identified in the 35 cacti plastomes.

**Table S4.** list of the plastomes with inverted repeats which are probably associated with the ~ 60-kb inversion

**Table S5.** Summary of assembled plastomes in this study

**Table S6.** Summary of sequencing data quality

**Table S7.** Assembly results of the Cactaceae plastomes

**Table S8.** The plant materials source of an additional related species (*Blossfeldia liliputana*) used in the phylogenomic study

**Table S9.** Primers were used in this study.

**Supplementary Figure:**

**Figure S1. Circular genome maps of the 35 plastomes**. Plastid genome map of 35 cacti species. The genes outside the circle are transcribed in the counterclockwise direction, and the genes inside the circle are transcribed in the clockwise direction. Different colors in genes represent different functions. The dark gray area and light gray area of the inner circle represent the ratio of GC content to AT content in the genome, respectively.

**Figure S2.** **A ~60-kb inversion observed in LSC region in Cactaceae plastomes.** a. The linear maps of plastomes. Take the comparison of Astrophytum myriostigma and Ariocarpus retusus as an example, the dotted lines in orange indicate inversions, the blue lines indicate the region of changes in genome position (not inverted). b. The dot-plots of As. myriostigma with itself and Ar. retusus. A pair of short-inverted repeats (SIR) was found at the place of the ~60-kb inversion in As. myriostigma c. The remaining 19 plastomes with this SIR structure. The red circle marked the SIR structure, and the blue circle marked some of the other repeats. It is worth noting that in genus Selenicereus, due to the expansion of IR regions, SIR structures were captured by IR region and its position has changed. This SIR structure perhaps mediated intramolecular recombination. Table S7 listed the positions and lengths of this SIR structures.

**Figure S3 Dot-plots among Portulaca oleracea and Pereskia aculeate, Opuntia microdasys.** The SSC region of Pe. aculeate and Op. microdasys shared the same rearrangement event. Mark the SSC region with a gray dotted box. A small rearrangement (6-kb) was observed in the LSC region of Pe. aculeate, and a large rearrangement (60-kb) was also observed in the LSC region of Op. microdasys.

**Figure S4 Dot-plots among Cacteae plastomes.** We only exhibited the plastomes that underwent genome rearrangement events.

**Figure S5 Dot-plots among Cacteae (Astrophytum myriostigma), Copiapoa hypogaea, Frailea castanea var. nitens and two Rhipsalideae plastomes (Rhipsalis cereuscula and Schlumbergera truncata ).** An 18 kb inversion was observed in the LSC region of Co. hypogaea and two Rhipsalideae species compared to the As. myriostigma, and the later had also observed another small-scale inversion of ~2.5 kb. Another rearrangement was observed in Fr. castanea var. nitens. The linear plastome maps showed the the genes associated with the above mentioned inversion and rearrangement.

**Figure S6 Another rearrangement event occurred in SSC region. a.** The Dot-plots among Pereskia aculeate and other cacti plastomes (take Astrophytum myriostigma as an example). The SSC region of most Cereoideae plastomes shared a small inversion and rearrangement, the deletion of multiple fragments in SSC possibly resulted in the loss of ndh gene suite. **b.** The linear plastome maps. The solid lines in blue indicate

rearrangement, and dotted lines in orange indicate inversion.

**Figure S7 Sanger sequencing results.** There were four different Sanger reads: the Sanger reads of F1+R1 and F2+R2 supports isomer 1, however, F1+R2 and F2 +R1 supports genome recombination (that is the isomer 2). The length of PCR products was 819 bp, 785 bp, 804 bp and 800 bp, respectively, which was slightly shorter than the estimated product lengths. Panels **a, b, c** and **d** are the alignment of the corresponding genomic regions with the PCR products. The genomic position is marked at the top of the base.
